# Patient safety culture research within the chiropractic profession: a scoping review

**DOI:** 10.1186/s12998-025-00605-z

**Published:** 2025-10-21

**Authors:** Debbie S. Wright, Maranda Kleppe, Brian C. Coleman, Martha Funabashi, Amy G. Ferguson, Richard Brown, Sidney M. Rubinstein, Stacie A. Salsbury, Katherine A. Pohlman

**Affiliations:** 1 Parker University, 2540 Walnut Hill Ln, Dallas, TX 75229 USA; 2 Private Practice, Courtenay, BC Canada; 3 Palmer College of Chiropractic, Davenport, IA USA; 4 Yale University, New Haven, CT USA; 5 Canadian Memorial Chiropractic College, Toronto, ON Canada; 6 Evidence Search Lab, Richardson, TX USA; 7 Richard Brown Chiropractic Consultancy, London, UK; 8 Vrije Universiteit, Amsterdam, The Netherlands

**Keywords:** Chiropractic, Patient safety, Safety management, Risk management, Adverse event, Spinal manipulation, Patient harm

## Abstract

**Introduction:**

A proactive patient safety culture is crucial in healthcare to minimize preventable harm and improve patient outcomes. This scoping review explores key themes, trends, and gaps in patient safety culture research within the chiropractic profession.

**Methods:**

A comprehensive literature search was conducted across 5 databases from inception to December 2024. Peer-reviewed, English-language studies focusing on chiropractic patient safety culture were included. Following scoping review methodology, articles were screened, data were extracted, and both qualitative and quantitative analyses were conducted. External consultants from patient safety-focused chiropractic groups were sought to review findings. Trends and themes were identified, and findings were compared against established patient safety frameworks to highlight research gaps and future directions.

**Results:**

Of the 3,039 screened articles, 65 met the inclusion criteria, spanning from 1990 to 2024, with 2 identified as randomized trials. Eight major themes were organized: (1) adverse event research, (2) clinical trial safety reporting, (3) patient safety attitudes, (4) clinical decision making, (5) informed consent, (6) reporting and learning systems, (7) office sanitization, and (8) general safety topics. Mapping these studies onto the Patient Safety Culture Pyramid framework revealed that 95% addressed safety performance, 81% covered safety processes, and only 23% explored beliefs and values. Comparisons with the WHO Global Patient Safety Action Plan framework highlighted advancements in clinical process safety while revealing research gaps in patient engagement, policy development, leadership, and interprofessional collaboration. Key recommendations include standardizing adverse event reporting, improving communication strategies, and developing structured approaches to patient and provider safety. External consultation provided minimal feedback requiring modifications.

**Conclusion:**

This review underscores significant advancements and gaps in chiropractic patient safety culture research, particularly in leadership, policy, and interprofessional engagement. Future research should focus on implementing and evaluating evidence-based safety interventions to enhance transparency, improve patient outcomes, and build public trust in chiropractic care. Direct stakeholder engagement, including with patients, is necessary to determine the most effective strategies for integrating patient safety within the global chiropractic profession.

**Supplementary Information:**

The online version contains supplementary material available at 10.1186/s12998-025-00605-z.

## Introduction

Patient safety is central to all domains of healthcare [1, 2]. While adverse events and healthcare errors are dominant topics and often the focus of safety initiatives [2], the World Health Organization (WHO) defines patient safety more broadly as “the absence of preventable harm to a patient and reduction of risk of unnecessary harm associated with health care to an acceptable minimum” [3]. This broader perspective incorporates structured activities and intangible aspects, such as cultivating specific cultures, systems, and behaviors, to enhance safety in healthcare delivery. These activities and aspects together embody the concept of “patient safety culture”.

The Institute of Medicine report, *To Err is Human*, describes patient safety culture as “a healthcare organization’s values, commitment, competencies, and actions in pursuit of patient safety” [2]. This definition further highlights that shared values and beliefs (i.e. culture), when aligned with an organization’s structures and systems, foster behavioral practices that enhance patient safety [4]. Systematic, coordinated, and consistent approaches to patient safety reduce risks, as well as the frequency and impact of avoidable harm [1, 3]. Therefore, efforts to understand and improve patient safety culture may yield more sustainable benefits than focusing solely on the harms themselves.

Chiropractic care is used widely for managing musculoskeletal conditions [5], and presents unique challenges to patient safety due to the diversity of practitioner approaches and practice settings, ranging from independent ambulatory clinics to those integrated within medical multispecialty groups (including hospitals). These variabilities in practitioner approaches and practice settings can impact patient safety in chiropractic care, with available knowledge limited to sparse data collected from surveys and surveillance systems [6–8]. A comprehensive systematic examination of patient safety culture initiatives within chiropractic is timely to establish an effective safety strategy, agree on a unified framework, and map a future research agenda.

This scoping review synthesizes the extent, range, and themes of patient safety culture research activities in the chiropractic profession. Additionally, our findings are mapped against the Patient Safety Culture Pyramid [9] and the WHO *Global Patient Safety Action Plan* [1], two robust and relevant patient safety frameworks, to identify areas of alignment and gaps in patient safety culture interventions or strategies. These efforts will establish a foundation of the current evidence in response to a recent “Call to Action” issued by the World Federation of Chiropractic (WFC) Global Patient Safety Task Force (now known and subsequently referred to as the WFC Global Patient Safety initiative), advocating for the advancement of patient safety in chiropractic [10]. We aim to inform the development of a tailored guide for future chiropractic-specific patient safety research, emphasizing the evaluation of patient safety attitudes, beliefs, performance measurements, and strategic interventions to foster a robust patient safety culture within the profession. To this aim, this scoping review will explore key themes, trends, and gaps in patient safety culture research within the chiropractic profession.

## Methodology

### Design

The scoping review methodology was selected to explore and map the breadth of evidence related to patient safety culture research in chiropractic. This study adheres to the well-established, multi-staged process outlined by Arksey and O’Malley [11] and further refined by Levac et al. [12] for scoping reviews. For reporting, the study followed the Preferred Reporting Items Systematic Reviews and Meta-Analyses for Scoping Reviews (PRISMA-SCR) guidelines [13] (see Additional File 3 for checklist). The protocol was registered prospectively with the Open Science Framework (OSF) Registry [14] and updated to include more specific inclusion and exclusion criteria listed in Stage 3 below. A protocol refinement was implemented to exclude best practice documents, clinical practice guidelines, and single-case studies from the study selection process. Given their nature, these sources do not constitute primary research and therefore do not align with the definition of patient safety culture research within the chiropractic profession, as outlined in Stage 1 below. This scoping review followed this multi-stage process: (1) research objective identification, (2) relevant study identification, (3) study selection, (4) data charting, (5) data synthesis and reporting, and (6) consultation.

#### Stage 1: Research objective identification

This scoping review aimed to explore and map the extent, range, and themes of patient safety culture research conducted within the chiropractic profession. We defined “patient safety culture” as attitudes, beliefs, practices, performance, or policy/procedure pertaining to the safe delivery of, or experience with, healthcare or the healthcare system, adapting the WHO definition [3] to reflect the chiropractic setting. Relevance to the chiropractic profession was defined as research addressing patient safety within chiropractic care, including—but not limited to—the unique risks and considerations associated with spinal manipulation therapy [15]. This focus highlights the critical need to identify, understand, and mitigate potential safety concerns inherent in hands-on therapeutic interventions, a cornerstone of chiropractic practice.

#### Stage 2: Relevant study identification

*Information sources.* A medical librarian and scoping review strategist (AF) conducted a comprehensive literature search. The following electronic databases were searched from inception until March 15, 2024, with an updated search performed on December 16, 2024: (1) MEDLINE (OVID) [16], (2) Index to Chiropractic Literature [17], (3) Allied and Complementary Medicine Database (EBSCO) [18], (4) Cumulative Index to Nursing and Allied Health Literature (EBSCO) [19], and (5) Google Scholar [20].

*Search strategy.* This strategy was developed collaboratively by study investigators with domain expertise (SR, KAP, DSW) and a medical librarian (AF). Before implementation, the strategy was peer-reviewed using the Peer Review of Electronic Search Strategies (PRESS) framework [21]. National Library of Medicine Medical Subject Headings (MeSH) and relevant keywords were used to search titles and abstracts for concepts such as chiropractic, patient safety, adverse events, and safety culture [22]. Backward citation searching, which involved reviewing the references cited by known relevant articles to identify additional pertinent studies, was used to identify further eligible articles [23]. A complete search strategy for each database can be found in Additional File 1.

#### Stage 3: Study selection

*Eligibility Criteria.* Eligible studies reported on patient safety culture research conducted within, related to, or including the chiropractic profession. Studies were included if they were written in English and consisted of quantitative, qualitative, mixed methods, or quality improvement studies, review articles, and brief reports reporting original investigations on patient safety culture. Exclusion criteria were non-original research articles (e.g., conference abstracts), opinion articles (e.g., editorials, letters to the editor, commentaries), case reports of a single individual, articles with inaccessible full-text versions, general best practice documents or clinical practice guidelines related to chiropractic care, non-peer-reviewed publications, and studies reporting solely on adverse event data derived from clinical studies without a specific focus on patient safety culture.

Search results were de-duplicated and imported into Covidence [24], a web-based application for citation management for systematic and scoping reviews. Two independent reviewers (KAP, DSW) screened titles and abstracts based on the a priori eligibility criteria*.* Discrepancies were resolved through consensus discussion, with input from a third reviewer (SAS) when necessary. Full-text articles deemed potentially eligible were independently screened by two reviewers (DSW, MK) using the same criteria, with any conflicts resolved by consensus discussion.

**Fig. 1:**
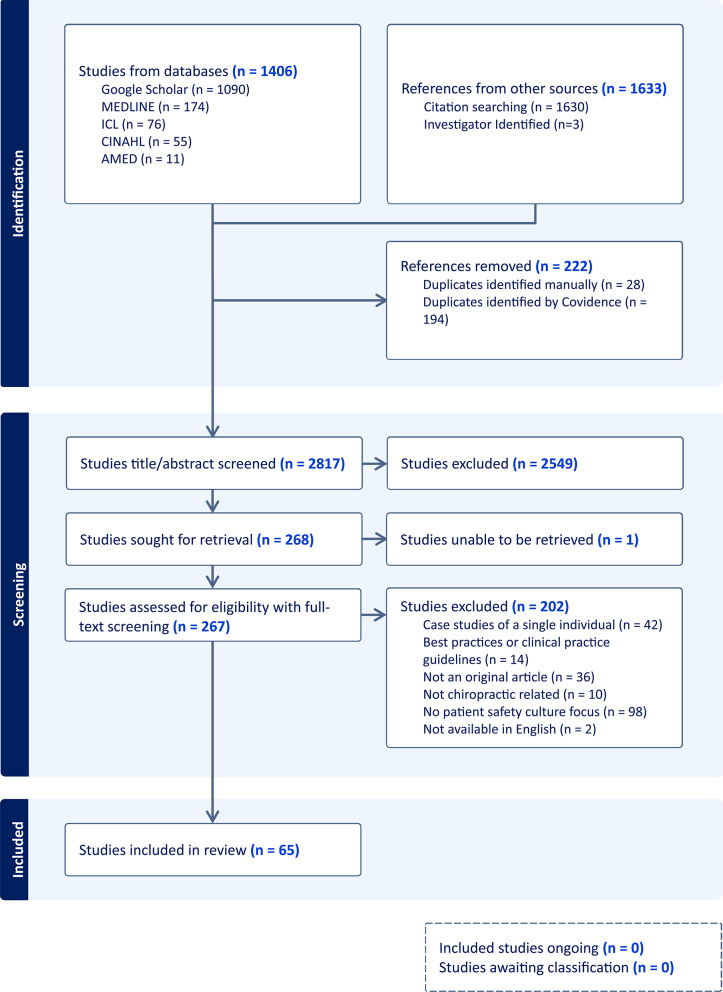
PRISMA flow diagram for patient safety culture research within the chiropractic profession: a scoping review

Backwards citation searching of included articles was used to identify additional unique studies. These were imported into Covidence and screened through the same title/abstract and full-text review process. Articles that met the inclusion criteria following full-text screening were used for data extraction. The flow of studies through the review process is detailed in a PRISMA flow diagram (Fig. 1).

#### Stage 4: Data charting

Two reviewers (DSW, MK) extracted data from the included studies using a standardized data extraction form, created a priori, pilot-tested with 5 studies, and subsequently refined. All data were extracted in batches independently by one reviewer (DSW, MK) and verified by the other, with each reviewer completing approximately half of the full extractions. Small group discussions and consensus (DSW, MK, KAP, SAS) determined the final dataset.

Extracted data included the following: study metadata and characteristics (e.g., author(s), year of publication, article title, aims and objectives, country of origin), population, methodology, intervention(s) and comparator(s) (if applicable), outcome measure(s) (if applicable), patient safety culture finding implications (most often found in the results or discussion sections), and study limitations. Additional recorded data included results related to patient *safety approaches*, patient *safety considerations*, and other noteworthy findings. *Safety approaches* encompassed findings on patient safety attitudes, opinions, and beliefs, while *safety considerations* included specific actions, tools, or systems designed to enhance patient safety culture within chiropractic practice.

In alignment with the WHO *Global Patient Safety Action Plan* [1], three reviewers (DSW, MK, KAP) independently identified data pertaining to strategic objectives. This process focused on documenting action items related to patient safety culture and identifying suggestions for future research.

#### Stage 5: Data synthesis and reporting

Data synthesis involved a qualitative analysis of the features of patient safety culture, such as attitudes and performance, extracted from the included studies. From these features, a thematic analysis was conducted by the study team (DSW, MK, KAP, SAS) to identify clusters of studies centered around similar domains within patient safety culture. Studies and themes were then mapped to two relevant patient safety frameworks: the Patient Safety Culture Pyramid [9] and the WHO *Global Patient Safety Action Plan* [1].

The Patient Safety Culture Pyramid illustrates the dynamic nature of safety culture, starting with core values and underlying assumptions at its base. These foundational elements support organizational components such as strategies, leadership, and policies. Above these, the safety climate is shaped by the attitudes, opinions, and processes of organizational members, ultimately culminating in safety performance at the peak, which is defined by behaviors and outcomes [9]. A quantitative analysis determined the percentage of studies addressing each pyramid level. Studies evaluating clinician or patient behaviors and outcomes, and adverse events themselves were classified as addressing *Performance*. Studies evaluating the values and beliefs underpinning patient safety culture were classified as addressing *Values*. To enhance clarity and practical applicability, we merged the middle two levels of the Patient Safety Culture Pyramid—climate and strategies—into a single category, *Processes*.

The *Global Patient Safety Action Plan*, developed by the WHO, comprises seven strategic objectives designed to guide stakeholders in improving patient safety and reducing preventable harm in healthcare globally. A quantitative and qualitative analysis was conducted, with each included study mapped to the seven strategic objectives and analyzed to determine whether it addressed them directly or indirectly. A study was classified as “direct” if it explicitly focused on one of the *Global Patient Safety Action Plan’s* objectives and investigated its principles as a primary focus. Alternatively, studies were categorized as “indirect” if they provided information relevant to a *Global Patient Safety Action Plan* objective but did so incidentally or as part of a broader discussion. While indirect studies did not directly address specific objectives, they provided valuable insights into understanding and implementing the *Global Patient Safety Action Plan* framework.

#### Stage 6: Consultation

Our findings were presented to three key chiropractic organizations with expertise in patient safety. First, the WFC Research Committee (n = 15) was consulted for feedback. As an official non-state actor of the WHO, the WFC represents the global chiropractic profession [25]. Second, input was sought from attendees of the Chiropractic Association of Alberta (CAA) Patient Safety Round Table (n = 26). The CAA is a member-based organization dedicated to advancing chiropractic care in Alberta, Canada [26]. Finally, the Royal College of Chiropractors (RCC) (n = 4) was engaged to provide practical insights. The RCC is a professional membership body, based in the United Kingdom, that upholds high standards of quality, safety, and professionalism in chiropractic practice, education, and research [27]. RCC also oversees the Chiropractic Patient Incident Reporting and Learning System (CPiRLS), a database enabling collaborating chiropractors to report, review, and discuss patient safety incidents. Stakeholder input focused on the study’s comprehensiveness—ensuring all relevant literature was included—the validity of the identified themes, and the appropriateness of the analytical frameworks. Additionally, feedback was requested on the clinical implications of the findings and recommendations for future research. All documentation related to the consultation process can be found in Additional File 4.

## Results

### Search results

Our search identified 3039 publications, with 222 duplicates removed (Fig. 1). An initial abstract and title screening of the remaining 2817 records yielded 268 articles for full-text review, excluding 2549 records for the following reasons: not being chiropractic-related, lacking a focus on patient safety culture, or not constituting original research. One full-text article could not be retrieved and was excluded. Following the full-text screening of the remaining 267 articles, 65 were included as relevant to the research question and meeting the inclusion criteria, with 202 publications excluded. A list of full-text excluded references and the reasons for their exclusion is provided in Additional File 2.

Table 1 describes the 65 publications included in this review [6–8, 15, 28–88]. The publication dates ranged from 1990 to 2024, with 3 articles published before 2000 [28–30], 16 between 2000 and 2009 [31–46], 32 between 2010 and 2019 (6,35–65), and 14 published in 2020 or later [7, 8, 77–88]. Among these, 16 articles described studies completed in Canada [6, 40, 47, 52, 53, 56, 59, 61, 67, 71–73, 75, 77, 80, 86], 15 in the United States [7, 28, 32, 33, 35, 36, 38, 41, 45, 50, 51, 55, 65, 76, 85], 10 in the United Kingdom [8, 15, 31, 34, 42, 43, 46, 48, 49, 58], 4 in Australia [29, 30, 57, 66], 2 in Italy [63, 64], 1 in Norway [60], and 17 were comprised of international research teams [37, 39, 44, 54, 62, 68–70, 74, 77–79, 81–83, 87, 88]. The publications included various study designs: cross-sectional studies (n = 17) [6, 8, 31–33, 37, 49, 51, 58, 70, 71, 73, 74, 76, 77, 79, 81], qualitative descriptive research (n = 13) [29, 30, 35, 42, 52, 54–56, 63, 67, 72, 75, 82], observational studies (n = 11) [7, 28, 34, 36, 39, 41, 43, 44, 60, 86, 87], systematic/scoping reviews (n = 10) [15, 40, 47, 62, 65, 68, 69, 83, 84, 88], narrative reviews (n = 6) [45, 46, 53, 63, 66, 85], case series (n = 4) [38, 50, 59, 80], randomized clinical trials (RCTs) (n = 2) [57, 78], an instrument development study (n = 1) [61], and a Delphi consensus panel (n = 1) [48].

**Table 1:** Overall summary of studies reporting on patient safety culture research in the chiropractic profession

Theme	Lead author, year (Country)	Objectives	Study design	Combined pertinent results	Implications	Patient safety pyramid levels addressed	WHO GPSAP strategic objectives addressed
Adverse event research	Terret 1992 (Australia)	To review, classify and explore the possible causes and mechanisms of injury from SMT to the low back	Qualitative descriptive research	A rationale for preventing complications from SMT could be based on knowledge of causes of complications, contraindications to SMT, diagnostic assessment of patients, and the selection and implementation of appropriate techniques. Patient-related and practitioner-related causes of complications are explored, and an algorithm for clinical decision-making is presented	A theoretical framework for preventing complications can be developed based on knowledge of the causes of reported complications and contraindications, knowledge and skills in diagnostic assessment, knowledge and skills in selecting and implementing techniques, identification strategies to prevent cauda equina syndrome, and using an algorithm in clinical decision-making relating to low back SMT	Performance Processes	Safety of Clinical Processes
Adverse event research	Rubinstein 2007 (Denmark and The Netherlands)	To describe both positive clinical outcomes and AEs following the first 3 treatments in a large cohort presenting with neck pain to 79 chiropractors	Observational study	While 50% of the study population experienced an AE, only 1% reported it much worse at the end of the study period. Therefore, these AEs should not be misconstrued as a measure or indication of harm or be confused with (the lack of) perceived recovery. AEs are most prevalent at the beginning of treatment and diminish thereafter in frequency, suggesting treatment approach modification may be beneficial at the start. It was found that symptoms often viewed as a consequence of treatment (headache, nausea, dizziness) were present in many subjects at baseline and could be erroneously attributed to the treatment	AEs following treatment are common and, in some cases, severe in intensity, but this study shows that the benefits of chiropractic care for neck pain seem to outweigh the potential risks. Many symptoms resembling an AE were present in nearly all the subjects at baseline and diminished in frequency in the population during the first three months. This demonstrates the need to record baseline status for concomitant symptoms to avoid erroneously ascribing their incidence to treatment	Performance Processes	Safety of Clinical Processes Information, Research, and Risk Management
Adverse event research	Vohra 2007 (Canada)	To systematically identify and synthesize available data on AEs associated with pediatric SMT	Systematic/ scoping review	Nine serious AEs occurred in children under 13 years of age. Practitioner surveys reported insufficient pediatric training for complementary and alternative medicine (CAM) providers. Collaborating with experts in pediatric education toward developing a standardized pediatric curriculum for CAM providers may offer a way forward. Such collaboration should involve the development of guidelines for medical referrals, joint integrative care between physicians and CAM providers, and developing a scope of practice for pediatric chiropractic and osteopathic care	SMT is common among children, and although serious AEs have been identified, their true incidence remains unknown. Patient safety demands greater collaboration between the medical community and other healthcare professionals, particularly chiropractors, so we can investigate and report SMT-related harms	Performance Processes	Safety of Clinical Processes Health Worker Education, Skills, and Safety Synergy, Partnership, and Solidarity
Adverse event research	Miller 2008 (United Kingdom)	To identify any AEs to chiropractic care occurring in the pediatric patient and to evaluate the risk of complications arising in the pediatric patient resulting from chiropractic care	Observational study	The Chiropractic Reporting and Learning System (CRLS), developed as part of clinical risk management to report all patient safety incidents, has been expanded to include parent reports of treatment side effects. This system has been instituted to prospectively collect all parental reports of negative reactions to pediatric treatment. This will result in a more accurate accounting of adverse treatment reactions in our clinic	Prospective investigations into the frequency and types of negative side effects are essential to document the safety of chiropractic treatment for pediatric patients and to learn about the types of reactions young children experience. This will allow chiropractors to inform parents of what is a normal reaction to chiropractic treatment and detect any risks of our treatment in pediatric care	Performance Processes	Safety of Clinical Processes Information, Research, and Risk Management
Adverse event research	Carnes 2010 (United Kingdom)	To seek an expert consensus definition of AEs in relation to manual therapy by exploring understanding and meaning using a modified Delphi technique	Delphi consensus panel	Major AEs are seen as medium to long-term, moderate to severe, and unacceptable; they usually require further treatment and are serious and distressing. Moderate AEs are described as major AEs but only moderate in severity. Mild and "not adverse" AEs are short-term and mild, non-serious; the patient's function remains intact and transient/reversible, and no treatment alterations are required because the consequences are short-term and contained	The definitions obtained following this Delphi study can be used to categorize or classify AEs in the context of manual therapy. Not only is a logical hierarchy presented, but also this definition allows for classifying those events that occur that may be regarded as "not adverse". The application of this definition may be helpful in both research and clinical settings for recording and documenting the nature, type, prevalence, and incidence of AEs to increase understanding and contribute to knowledge in this area	Performance Processes	Information, Research, and Risk Management
Adverse event research	Leach 2010 (United States)	To present a retrospective case series of patients with symptoms and signs suggesting a stroke and discuss the potential for education/promotion initiatives in chiropractic	Case series	Patients will seek chiropractic care when they have signs and symptoms suggestive of a stroke. Education programs for recognizing early signs of stroke in chiropractic clinics are necessary, and the use of the FAST tool may be appropriate. Chiropractors have a role in educating patients on the role of health and wellness in stroke prevention and prompting lifesaving referrals of emergency presentations	Patients with symptoms and signs of stroke may present to chiropractors. To avoid potentially catastrophic delays in receiving potentially lifesaving treatment, chiropractors must be informed regarding risk factors, prevention, screening, and early recognition of symptoms and signs. Chiropractic health education research must evaluate chiropractors' training in this area	Performance Processes	Safety of Clinical Processes Health Worker Education, Skills, and Safety
Adverse event research	Carlesso 2011 (Canada)	To describe how patients define AE associated with manual therapy techniques	Qualitative descriptive research	This exploratory study identified that patients define an AE in manual therapy through a multi-factorial process determined by antecedent, sequelae, and universal elements and that how they view an AE is, to some extent, modifiable. Trust, patient education, expectations, treatment experience, presenting with an acute or chronic condition, body awareness, and weighing benefits versus harm can influence patient perceptions of an AE. Further study of what responses patients view as acceptable or normal and not adverse is required to validate these findings. This, in turn, can influence future data collection of AEs in manual therapy studies	Standardized definitions of AE in MT can be created in a multi-stakeholder process inclusive of the patient perspective to help reconcile differences between patient and practitioner viewpoints. However, before proceeding with the standardization process, it is suggested that additional studies be conducted to inform the patient's perspective further. Areas requiring development include the perspective of patients who have sustained moderate to major AEs in relation to manual therapy treatment and the exploration of patients' opinions on what symptoms are adverse or not	Performance Processes Values	Safety of Clinical Processes Patient and Family EngagementInformation, Research, and Risk Management
Adverse event research	Walker 2013 (Australia)	To establish the frequency and severity of adverse effects from short-term usual chiropractic treatment of the spine when compared with a sham treatment group	RCT	Results suggest that many AEs experienced after chiropractic treatment result from natural history variations or nonspecific effects. Some studies demonstrated that the AEs reported by participants in either the placebo or the sham arm mirror the AEs in the active intervention arm. An expectation of AEs coupled with not wanting to experience AEs may promote nonspecific effects contributing to AEs. Framing information about AEs in positive terms rather than negative terms can lead to a lower AE rate	AEs resulting from chiropractic are common. Most AEs resulting from chiropractic are benign and transitory. A substantial proportion of AEs resulting from chiropractic seem to be due to nonspecific effects	Performance Processes	Safety of Clinical Processes Patient and Family Engagement
Adverse event research	Hebert 2015 (Australia and The Netherlands)	To systematically search the literature for studies reporting serious AEs following lumbopelvic SMT and to describe the case details	Systematic/ scoping review	Clinicians should be diligent in screening patients and maintain a high index of suspicion for cauda equina syndrome when patients present with one or more of the following signs or symptoms: (1) bladder and/or bowel dysfunction, (2) reduced sensation in the saddle area, or (3) sexual dysfunction with possible neurologic deficits of the lower limb. Accurate reporting of provider type and treatment details in research will aid in accurate estimates of incidence and the exploration of possible risk factors or predictors of serious AEs that are likely to enhance clinical decision-making for healthcare providers and help patients make informed healthcare decisions	Information such as the SMT description, the patient's pre-SMT presentation, and AE details were lacking. Additional high-quality research is needed to estimate better the incidence of AEs associated with lumbopelvic SMT and to elucidate the relationship between this therapy and the types of AEs reported	Performance	Safety of Clinical Processes Information, Research, and Risk Management
Adverse event research	Puentedura 2015 (United States)	To retrospectively analyze all available documented case reports in the literature describing patients who had experienced severe AE after receiving TJM to their thoracic spine	Systematic/ scoping review	Practitioners should use appropriate force when delivering thoracic manual therapy and screen for contraindications (though there is a lack of valid and reliable screening tools). They are cautioned to take a thorough history and look for common preexisting conditions, including osteopenia/osteoporosis. More standardized information for AE reporting in research is needed	Clinicians need to manage manipulation force and perform a thorough examination to mitigate the likelihood of AEs. Based on the results of this review, we propose that cases regarding AEs in the thoracic spine should provide more standardized information	Performance	Safety of Clinical Processes Information, Research, and Risk Management
Adverse event research	Todd 2015 (Australia)	The purpose of this study was to review the literature for cases of AEs in infants and children treated by chiropractors or other manual therapists, identifying treatment type and if a preexisting pathology was present	Narrative review	When treating the pediatric population, practitioners should consider appropriate history, screening, and examination methods, modify the level of force, and implement more comprehensive levels of AE recording. Undergraduate training requires more exposure to many patients (including children)	Serious AEs in infants and children receiving manual therapy are exceedingly rare. Thorough history and examination, appropriate technique selection/application, systematic AE reporting, and rigorous education may reduce AEs across all manual therapy professions	Performance Processes	Safety of Clinical Processes Health Worker Education, Skills, and Safety
Adverse event research	Swait 2017 (United Kingdom)	To characterize and summarize the available literature on risks and to describe implications for clinical practice and research	Systematic/ scoping review	Benign AEs are common, affecting 23–83% of adult patients. They are mostly mild-moderate and transient and commonly include musculoskeletal pain, stiffness, and headache. Patients presenting with moderate to high levels of neck disability may have an approximately three times greater likelihood of experiencing transient neurological symptoms. Cervical manipulation may carry a greater risk compared with cervical mobilization or thoracic manipulation in patients with neck pain. Non-specific effects or natural progression may also contribute to reporting benign AEs. Conducting a thorough case history and examination is essential before treating to screen for risk factors	Preexisting pathology may raise the risk of AEs. Therefore, detailed screening for known risk factors is essential before applying any manual therapy to a patient of any age. By disseminating their case details first-hand, clinicians can also help elucidate uncertainties arising around serious AEs due to inaccurate case reporting	Performance Processes	Safety of Clinical Processes Patient and Family Engagement Information, Research, and Risk Management
Adverse event research	Zorzela 2018 (Canada)	To develop and test a tool to assess the causality of direct and indirect AEs associated with therapeutic interventions	Qualitative descriptive research	A tool was created and validated to adjudicate the causality of AEs associated with a health intervention. It assesses direct and indirect harm (where the intervention caused a delay in diagnosis or treatment, such as the delay is the cause of the harm). The assessment of indirect harms is important to understand the factors leading to the event and, therefore, avoid the recurrence of similar events	A tool to assess the causality of AEs was developed and tested. Causality assessment is a critical part of assessing AEs. We proposed a novel method to assess direct and indirect harms related to the product(s), device(s), practice, or a combination thereof	Processes	Safety of Clinical Processes Information, Research, and Risk Management
Adverse event research	To 2020 (Canada)	To identify commonalities among cases of rib fractures after SMT; discuss chiropractors' case management perspectives; and propose strategies for prevention/ management	Case series	Chiropractors learned it was important to (1) verify and update factors associated with rib fractures, display updated Bone Mineral Density (BMD) information in the file, and mitigate risk by modifying treatment; (2) communicate before SMT and/or after an AE (including prior informed consent), while understanding patient's perspectives post-AE is important; and (3) enhance student education on AE management by promoting a learning vs. blame culture	Important lessons can be learned from AEs despite their infrequent occurrences. As patient safety is a global healthcare challenge, chiropractors need to be leaders in creating an open and constructive patient safety environment within their profession. Prevention and mitigation factors for rib injury include verifying and updating potential AE contributing factors, open and honest communication with the patient, and enhancing student education to improve the patient safety culture	Performance Processes Values	Safety of Clinical Processes Patient and Family Engagement Health Worker Education, Skills, and Safety Information, Research, and Risk Management
Adverse event research	Weis 2021 (Canada and United States)	To update a previous critical review of AEs in pregnant and postpartum populations	Systematic/ scoping review	AEs following SMT in pregnant/postpartum patients appear to be scarce. Future research should not only report the presence or absence of AEs but also determine the AEs that occur at each of the different pain locations. Contraindications to SMT are evident during a careful history/physical exam, and practitioners should consider prothrombin and joint laxity risk facts when determining a treatment plan. Higher-risk pregnant women should be treated with additional caution with counseling on risks and education about neurovascular complications	Although we call for improved reporting of such AEs in all papers going forward, these events appear rare. Future research should focus on properly reporting all AEs while assessing the efficacy of appropriate treatment options for these populations	Performance Processes	Safety of Clinical Processes Information, Research, and Risk Management
Adverse event research	Funabashi 2022 (Canada)	To map the scientific literature defining AEs and their respective classification systems following spinal or peripheral joint manipulation and mobilization for musculoskeletal conditions in an adult population	Systematic/ scoping review	The lack of standardization of terms, definitions, and classification systems may contribute to the lack of AE reporting systems within the professions that use joint manipulation or mobilization interventions. Determining causality between the delivery of a manual intervention and AE is challenging. A standardized operational definition of an AE is needed to facilitate this process. Developing a standardized classification for AEs following spinal and peripheral joint manipulation and mobilization could provide a common language for all professions that use these interventions and facilitate identification, reporting, and communication about AEs, promoting interprofessional learning and advancing patient safety	A consensus on standardized terms, definitions, and classification systems for AEs related to these interventions is urgently needed. It could advance strategies to enhance patient safety for all professionals who deliver these interventions	Performance Processes	Safety of Clinical Processes Information, Research, and Risk Management Synergy, Partnership, and Solidarity
Adverse event research	Dolbec 2024 (Canada)	To evaluate the feasibility to conduct a pragmatic prospective study aiming to report the frequency of immediate and delayed (48 h post-treatment) AEs associated with manual therapies in children of 5 years or less	Observational study	The results support the feasibility of conducting a large-scale study evaluating AEs reported following chiropractic care in children of 5 years or under using the electronic SafetyNET reporting system. Findings suggest that targeting clinicians who have a marked interest in the treatment of children ensures clinicians' engagement. Using a reporting system available on an electronic platform resulted in a low attrition rate. Two main challenges were identified by participating clinicians about the feasibility of participating in the study: complexity with patient consent to participation before treatment and time burden for the clinician and their clinic staff. However, these challenges were related to study procedures and not related to AE reporting processes	Further patient-safety research is necessary in the pediatric population to properly inform legal guardians and clinicians about the potential AEs associated with manual therapies and thus provide more fully informed consent to care	Performance Processes	Safety of Clinical Processes Information, Research, and Risk Management
Assessing clinical trial safety reporting	Carlesso 2010 (Canada)	To synthesize the literature that has reported AEs related to both cervical manipulation and mobilization techniques across professions at the highest possible level of evidence	Systematic/ scoping review	This review identifies the need for more stringent reporting of AEs in manual therapy efficacy trials. This also encompasses the implementation of standardized definitions of AEs. In most eligible studies, increased neck pain and headaches were the most commonly captured AEs. These are often primary outcomes for efficacy studies. Neck pain is typically measured on a continuum and is prone to fluctuation. If studies continue to report increased neck pain as an AE, there should be a consensus as to the threshold where the degree of increase in neck pain is deemed adverse. Definitions of AE need to address aspects beyond an increase in symptoms. Severity, duration, and onset will help make these differentiations possible	If trials start to incorporate the capturing of AE in their study design and adopt the CONSORT Statement extension on harm reporting guidelines, valuable information on mild to moderate AEs can be added to this area. Large-scale observational studies are the most appropriate to detect AEs and are likely the only way serious AEs will be captured. Such studies should be conducted across manual therapy professions, not just chiropractic	Performance	Safety of Clinical Processes Information, Research, and Risk Management
Assessing clinical trial safety reporting	Turner 2011 (Canada and United States)	To assess the quality of safety reporting in complementary and alternative medicine (CAM) RCTs, and to explore the influence of different trial characteristics on the quality of safety reporting	Qualitative descriptive research	Safety reporting across trials of CAM interventions is inadequate and often ignored altogether. This has implications for systematic reviewers, as the synthesis of harms from individual trials will be seriously compromised. CONSORT guidance needs to become the standard procedure for authors and editors when presenting findings for all trials	The overall conclusions of both evaluations are consistent in that the percentage of trials reporting harms and the adequacy of their reporting are largely inadequate irrespective of defined predictors. We hope that these data will impress journal editors, who, in turn, will now endorse reporting guidelines as an important way to improve the quality of reporting harms	Performance Processes	Information, Research, and Risk Management
Assessing clinical trial safety reporting	Marchand 2015a (Italy)	To perform a literature search to identify relevant studies on pediatric SMT and chiropractic manipulative therapy and to assess if safety terminology was consistent with the International Conference on Harmonization of Technical Requirements for Registration of Pharmaceuticals for Human Use (ICH)	Narrative review	The presented template for chiropractic safety incident reports could increase coherence and ease of information gathering/comparison in future research. It will improve the quality of information gathered and the ability to compare between studies. It is important to record patient diagnoses, consider using consistent terminology across authors, and prospectively gather evidence on the occurrence of AEs	Recording, classifying, and differentiating between side effects and adverse reactions will help promote safer pediatric practice. By encouraging clinicians to report safety incidents constructively, data could be gathered and analyzed. A list of normal side effects and out-of-the-norm adverse reactions could allow researchers to better search for and identify risk factors in the pediatric population	Performance	Safety of Clinical Processes Information, Research, and Risk Management
Assessing clinical trial safety reporting	Gorrell 2016 (Australia and Canada)	To describe the extent of AEs reporting in published RCTs involving SMT, and to determine whether the quality of reporting has improved since publication of the 2010 Consolidated Standards Of Reporting Trials (CONSORT) statement	Systematic/ scoping review	Our review highlights the inadequate reporting on all classifications of AEs. Quantifying the relative incidence of all AE classifications is required to accurately inform patient choice about SMT. One of the main obstacles to the adequate reporting of AEs associated with SMT is the lack of a standardized definition. Our findings support the literature that reports that while mild and moderate AEs are relatively common, major events are extremely rare. However, RCTs may not be the most accurate way to gather incidence data on major AEs, as the poor quality of their reporting has compounded the problem	Although the reporting of AEs has increased since the introduction of the 2010 CONSORT guidelines, the current level should be seen as inadequate and unacceptable. We recommend that authors adhere to the CONSORT statement when reporting AEs associated with RCTs. Standardization of the nomenclature and the development of a uniform classification system, as well as the development and validation of tools used to collect AE data, are necessary to allow the pooling of data for meta-analysis in the future	Performance	Information, Research, and Risk Management
Assessing clinical trial safety reporting	Gorrell 2023 (Australia and Switzerland)	To describe if there has been a change in the reporting of adverse events associated with spinal manipulation in randomised clinical trials (RCTs) since 2016	Systematic/ scoping review	The percentage of included studies reporting on AEs increased from 38% in 2016 to 61% in the current study. Of the 61% only 38% reported directly on AEs and only 23% provided an explicit definition of AEs—reporting of adverse events in RCTs involving spinal manipulation remains poor and is not consistent with established standards. Further complicating this issue is the vast heterogeneity of terms (ie, ‘adverse effect’, ‘side effect’, ‘harm’, etc.) used to describe adverse events. This is disappointing given that there have been many calls in the literature for the improvement of adverse events reporting in RCTs, and for the development and use of standardised definitions and classification systems	By reporting these analyses in a single manuscript, we hope it is clearer for readers to identify that the current level of reporting of adverse events associated with spinal manipulation in RCTs is both poor and not consistent with established standards	Performance	Safety of Clinical Processes Information, Research, and Risk Management
Assessing clinical trial safety reporting	Stickler 2023 (United States)	To describe variability in spinal manipulation technique details and AE documentation of SMT during pregnancy	Narrative review	There is variability in reporting SMT techniques and documenting the severity of AEs in the pregnant population, as well as inconsistency in reporting on the technical aspects of the treatment. Such variability may impact determining the appropriateness and relative risk of applying SMT in the pregnant population and make reproducing methods in future investigations difficult	Reporting of AEs after SMT should be standardized in the clinical and research settings to inform safety recommendations. Further research is needed to determine if SMT is an effective and safe treatment for pregnant women with musculoskeletal pain	Performance	Safety of Clinical Processes Information, Research, and Risk Management
Patient safety attitudes, opinions, and practice	Pohlman 2016a (Canada and United States)	To evaluate attitudes and opinions of doctors of chiropractic specializing in pediatric care toward patient safety	Cross sectional study	Pediatric chiropractor's scores were higher (suggesting more positive attitudes toward patient safety) than Agency for Healthcare Research and Quality (AHRQ) medical doctors and offices, except for information exchange with third-party payors. The patient safety items and quality issues identified as irrelevant to their practice were updating a patient's medication list and following up on critically abnormal results from a lab or imaging test within 1 day. There is value in developing a patient safety culture database for SMT providers, as it would allow more advanced quality improvement initiatives to be designed and their impact measured. We recommend that future research initiatives on patient safety include this survey and the development of such a database	This is the first survey to evaluate patient safety attitudes and opinions from the pediatric chiropractic profession. The survey revealed that respondents self-reported positively across most patient safety dimensions, leaving room for improvement in a few areas, such as medication documentation and abnormal diagnostic laboratory feedback. This population is well suited to implement a patient-safety reporting system	Performance Processes	High Reliability Systems Safety of Clinical Processes Patient and Family Engagement Information, Research, and Risk Management
Patient safety attitudes, opinions, and practice	Porcino 2017 (Canada)	To assess chiropractic and naturopathic doctors' knowledge, attitudes, and behavior with respect to the pediatric patients in their practice	Cross sectional study	Chiropractors and naturopaths rated lecture training in pediatric care as adequate, while hands-on training was rated as inadequate. Even with enhancements to pediatric education in the past five years, provider comfort (somewhat/very comfortable) was significantly lower for those trained post-2009	There is a need to enhance pediatric training to address gaps identified by practitioners; emphasis should be given to conditions that enhance patient safety. We call for greater collaboration between conventional and complementary therapy educational institutions to share core pediatric curricula about conditions that could harm children if not recognized to help future healthcare providers of all disciplines meet the needs of children in their care	Performance Processes	Health Worker Information, Skills, and Safety Synergy, Partnership, and Solidarity
Patient safety attitudes, opinions, and practice	Funabashi 2018 (Canada)	To develop or adapt, validate and implement an assessment tool to measure patient safety attitudes and opinions of community-based SMT providers	Cross sectional study	SMT providers had similar or better patient safety dimension scores compared to the Agency for Healthcare Research and Quality (AHRQ) 2016 medical offices database. Work pressure & pace dimension scored much higher than the AHRQ database, indicating that respondents often felt rushed and may have too many pts for the time available. Identified barriers to a reporting system included time pressure, patient concerns, a lack of a clear definition of a reportable event, and potential regulatory and legal implications. SMT provider respondents scored lower than medical officers in items related to regular medical list updates and abnormal lab/imaging tests not being followed up by 1 business day. There is a need for processes and systems to accommodate providers' busy workloads and reduce potential staff burnout	By understanding SMT providers' opinions and attitudes towards patient safety and identifying areas for improvement, organization-specific strategies can be developed to support a culture of patient safety and promote quality improvement	Performance Processes Values	High Reliability Systems Health Worker Education, Skills, and Safety Information, Research, and Risk Management
Patient safety attitudes, opinions, and practice	Salsbury 2019 (United States)	To describe DC attitudes toward integrative medicine and interprofessional care for older adults with back pain, and to identify DC self-reported interdisciplinary referral and co-management patterns for older patients	Cross sectional study	Chiropractors scored highly on a subscale for the perceived safety of integrative medicine, moderately for openness to interprofessional practice and readiness to refer, and low on the willingness to learn from other paradigms subscale. Chiropractors expressed some lack of confidence in managing back pain in older patients and may need additional education. Clinical experiences in which chiropractic students care for older patients alongside primary care providers, mental health specialists, and social services professionals could develop these needed skill sets and improve clinical outcomes	Doctors of chiropractic may benefit from interdisciplinary geriatric education programs that enhance their knowledge about team-based collaboration with biomedical providers and integrative medicine strategies for caring for older people with back pain. Health care delivery systems that bring together manual therapists with different expertise may be a starting point to develop effective collaboration models for doctors of chiropractic working with older adults	Processes Values	Health Worker Education, Skills, and Safety Synergy, Partnership, and Solidarity
Patient safety attitudes, opinions, and practice	Funabashi 2020 (Canada and United States)	To identify beliefs, perceptions and practices of chiropractors and patients regarding benign AEs post-SMT and potential strategies to mitigate them	Cross sectional study	Most clinicians believe benign AEs occur infrequently post-SMT, and just over half of patients reported experiencing one. Both groups did not believe they were related to specific SMT techniques or anatomical areas but thought mitigation strategies might be possible. Patient education may be the most beneficial strategy, and provider-patient communication needs to be improved concerning its rationale	Both clinicians and patients believe benign AEs occur post-SMT, with pain/soreness, headache, and stiffness being the most common benign AEs. However, clinicians' and patients' beliefs related to strategies to mitigate benign AEs post-SMT differed primarily in applying icing and stretching. Aligning beliefs and perceptions of clinicians and patients related to mitigation strategies may contribute to reducing benign AEs post-SMT	Performance Processes Values	Safety of Clinical Processes Patient and Family Engagement
Patient safety attitudes, opinions, and practice	Pohlman 2020c (Canada, United States, and United Kingdom)	To evaluate patient safety attitudes of clinic stakeholders in 5 international chiropractic teaching programs	Cross sectional study	For most Agency for Healthcare Research and Quality (AHRQ) survey domains, the chiropractic programs had significant gaps compared with the Canadian community-based providers and the medical academic programs in patient safety attitudes, with qualitative findings from the chiropractic sample accentuating specific areas for improvement. Explanations for these gaps need further exploration but may be explained by the initial focus of the patient safety culture movement encouraged on the medical systems due to the higher prevalence of severe patient conditions, shorter visit times affecting patient-provider relationships, more funds available to direct toward this initiative, and improved organizational structure/employees understanding of safety culture within most medical systems allowing for less fear of consequences for staff who identify/report errors. Our findings highlight the need for continued focus on patient safety training for chiropractic students and clinicians alike	Clinic stakeholders identified multiple areas for improvement in patient safety within chiropractic educational programs. Teamwork and information exchange were considered strengths in these settings. Respondents emphasized the need for patient-centered administrative priorities, improved work pressure/pace, standardized office processes, and enhanced communication about patient care between clinic stakeholders. Student feedback articulated the emotional side of missed opportunities in patient safety and suggested key areas for additional training for trainees and faculty alike	Performance Processes Values	High Reliability Systems Safety of Clinical Processes Information, Research, and Risk Management
Patient safety attitudes, opinions, and practice	Alcantara 2021 (United States, Australia, and Canada)	To assess the safety attitudes, safety practices and contributing factors to patient safety utilizing the SCORE instrument, and to determine variables that might contribute to an improved safety climate and lower burnout	Cross sectional study	Healthcare worker burnout and work-life balance are missing topics in safety culture assessment instruments. In addition to higher safety climate ratings and lower burnout ratings, most chiropractor responders in this study provided a positive outlook in their work environment with growth opportunities, the ability to participate in decision-making, and advancement in practice. In this study, decision-making, teamwork climate, and local leadership heavily influenced the safety climate in the workplace. Patient Safety Leadership Walkrounds were identified as a deficit among this group. Most chiropractor responders indicated a more optimal work-life balance, with the strongest association with burnout climate and personal burnout	Decision-making, teamwork, and local leadership were found to heavily influence the safety climate in the chiropractic workplace among the study's respondents. Most chiropractors could benefit from a patient safety leadership walkaround to discuss safe care delivery	Performance Processes	High Reliability Systems Safety of Clinical Processes Health Worker Education, Skills, and Safety
Patient safety attitudes, opinions, and practice	Funabashi 2021 (Canada, United States, and Denmark)	To describe perceptions of patient safety among chiropractors and physiotherapists who provide SMT	Qualitative descriptive research	Five common themes emerged in chiro/physio perceptions on patient safety: Doing Our Best for Patient Safety, Putting Patients First, Working and Learning Together, Organizing Practice Processes, and Considering Practitioners Identity. However, diversity exists in where they are on the safety culture continuum (some more advanced, others less so). SMT providers do not share a common framework about what patient safety is and struggle to identify/manage concerns. Possible difficulty in implementing/sustaining patient safety initiatives is due to cultural differences in each clinical setting	These findings add to our understanding of how manual therapists offering SMT think about patient safety in their clinical practices. Work is needed to improve patient safety culture and patient safety itself by building interprofessional teams, improving knowledge exchange, identifying AEs, and providing learning opportunities	Performance Processes Values	High Reliability Systems Safety of Clinical Processes Patient and Family Engagement Health Worker Education, Skills, and Safety Information, Research, and Risk Management Synergy, Partnership, and Solidarity
Clinical decision making	Smith 2006 (United States)	To describe the intra-professional referral patterns amongst chiropractors, describe the inter-professional referral patterns between chiropractors and conventional trained medical primary care physicians (MDPCPs), and to identify provider characteristics that may affect these referral behaviors	Cross sectional study	Chiropractors and MDPCPs tend to engage in informal practices when recommending or referring their patients to the other profession, and this lack of a direct formalized referral relationship has implications for efficiency, quality, and patient safety in the health care delivery system. This reveals concerns that need to be addressed to improve coordination and continuity of care for patients shared between these provider types. Opportunities to readily engage in informal ongoing dialogue, such as curbside consults, can implicitly standardize and improve practices of care within disciplines and between these groups	Chiropractors tend to engage in informal practices when recommending or referring their chiropractic patients to the care of an MDPCP. The lack of a direct formalized referral relationship between chiropractors and MDPCPs affects efficiency, quality, and patient safety in the health care delivery system. Future studies must identify facilitators and barriers to developing positive inter-professional referral relationships between chiropractors and MDPCPs	Performance Processes	High Reliability Systems Synergy, Partnership, and Solidarity
Clinical decision making	Rubin 2007 (United States)	To illustrate the use of triage skills in a primary care, chiropractic pediatric practice. This is examined both in the new patient setting and during visit-to-visit protocol	Case series	Triage in chiropractic practices can aid in a clinician's differential diagnostic abilities, either at the initial or regular office visits. Most patients triaged to emergency referral were regular, presenting to see if an emergency room (ER) visit was necessary. Referral and co-management of pediatric patients should be expected regularly	Triage can increase the likelihood that patients receive an optimal, safe, and effective care. It can also strengthen the chiropractor's abilities to manage patients with a variety of challenges and aid the chiropractor in identifying patients who require co-management or emergency care	Performance Processes	Safety of Clinical Processes
Clinical decision making	Rubinstein 2008 (Denmark and The Netherlands)	To examine which variables may predict AEs in subjects undergoing chiropractic treatment for neck pain	Observational study	Even though we examined a large number of independent variables characterizing the patient, chiropractor, and treatment delivered, only three variables were found to be predictive of AEs, namely, the reported use of rotation by the chiropractor, working status of the patient (sick leave or workers comp), and longer duration with neck pain in the preceding year. A fourth variable was protective: those patients who had visited the general practitioner (GP) in the 6 months before the first visit were less likely to have an event	Although we examined many independent variables characterizing the patient, chiropractor, and treatment delivered, only three variables were found to predict AEs, and a fourth variable was found to be protective. The fact that our models were highly predictive suggests that only a few select variables are necessary to predict who will have an AE	Performance	Safety of Clinical Processes
Clinical decision making	Miller 2009 (United Kingdom)	To review the literature that investigated AEs of chiropractic care for the pediatric patient and reflect upon risk reducing behaviors in our offices to improve safety for children under our care	Narrative review	Few RCTs address chiropractic treatment of the pediatric patient, and only rarely do they address safety issues. Virtually all of the severe sequelae from misdiagnosis or treatment stemmed from the lack of recognition of occult pathology. Chiropractors should look at practice through a risk-reducing lens and adopt behaviors that help to minimize risk, including prospective reporting of all patient safety incidents	Based on the published literature, manipulation, when given by a skilled chiropractor with years of training carried out with low forces recommended for pediatric care, has few side effects in healthy infants and children, and their recorded incidence is exceedingly low. Nothing is of greater importance in our pediatric practice than taking a proactive stance to incorporate safe practice strategies into daily practice and to report any incidents with the goal of safety and protection for all patients	Performance	Safety of Clinical Processes Health Worker Education, Skills, and Safety Information, Research, and Risk Management
Clinical decision making	Smith 2010 (United States)	To explore whether chiropractors may contribute to advancing drug safety initiatives by identifying potential adverse drug events in their chiropractic patients, and by bringing suspected adverse drug events to the attention of the prescribing clinicians	Cross sectional study	There is a potential role for non-prescribing clinicians such as chiropractors to identify instances of suspected adverse drug reactions or medication intolerance, nonadherence, medication errors, or other problems with prescribed drug regimens. We recommend advancing a multidisciplinary consensus-based approach to improving the integration and coordination of care for patients with suspected adverse drug reactions in the community. Prescribing clinicians, nonprescribing clinicians, and informed others, such as pharmacists, should jointly develop and disseminate appropriate standards for intra-disciplinary and inter-disciplinary clinical case management of suspected adverse drug reactions in their shared patients. Optimal collaborative care should include appropriate documentation and communication of useful information, timely notification, and diligent follow-up	Our findings suggest that chiropractors or other non-prescribing clinicians can detect potential adverse drug events in the community. These detection and reporting mechanisms should be standardized, and policies related to clinical case management of suspected adverse drug events in chiropractic patients should be developed. More scholarly attention is warranted to inform further expert consensus about what constitutes a useful and necessary skillset (and requisite preparatory training) of nonprescribing clinicians to detect adverse drug events and to ensure that suspect cases are brought to the attention of the prescribing clinician in a timely and useful manner	Performance Processes	Safety of Clinical Processes Health Worker Education, Skills, and Safety Synergy, Partnership, and Solidarity
Clinical decision making	Sadr 2012 (Canada)	To explore the experience of chiropractic treatment for pregnant women who have LBP, as well as their chiropractors in providing care for such patients	Qualitative descriptive research	All the treating chiropractors directly stated in their interviews that they believed chiropractic treatment for their pregnant patients was safe and that they had seen no AEs. All the patients stated in their interviews that they believed chiropractic treatment was safe and had not experienced any AEs after any treatment. Patients also described their comfort levels changing with particular treatments throughout the pregnancy, while their chiropractors generally modified the treatments to make their patients feel safe and comfortable	Pregnant patients appear to have benefited from chiropractic treatment, including SMT, soft tissue therapy, and exercise therapy. The pregnant patients or their chiropractors reported no AEs, and the patients involved reported being generally satisfied with their care. They had positive outcomes in reducing their low back pain symptoms and improved range of motion and overall function	Performance Processes	Safety of Clinical Processes Patient and Family Engagement
Clinical decision making	Wangler 2013 (United Kingdom)	To investigate how chiropractors manage potentially risky clinical scenarios, and how chiropractors perceive the safety climate in their workplace	Cross sectional study	Swiss/UK chiropractors were moderately to highly positive about teamwork, work pressure, staff training, process and standardization, communication openness, and patient tracking/following-up regarding safety culture. With clinical scenarios, chiropractors are generally unlikely to stop treatment but likely to re-evaluate, reflect on diagnosis, and alter the treatment approach. Incident reporting was found to be an unlikely option and comments revealed that this may be due to a perceived connection of reporting with guilt and error	Swiss and UK chiropractors tend to manage potentially risky clinical scenarios by re-evaluating their care and changing their approach. Safety incident reporting to an online system is currently an unlikely course of action due to previously recognized barriers that need to be addressed to encourage wider use	Performance Processes Values	Health Worker Information, Skills, and Safety Information, Research, and Risk Management
Clinical decision making	Innes 2018 (Australia and France)	To explore chiropractic students' abilities to correctly identify proper management of case scenarios, and understand when treatment is indicated	Cross sectional study	Students were good at identifying indications to continue care, and the results generally improved with each year of study. However, the scenarios that reflected non-indication for continued care had much worse results and did not improve in higher years. Encouragingly, for an obvious contraindicated neck scenario, the results were good from the beginning and got better, but for a contraindicated LBP scenario, the results started rather badly in year 3, then improved over the program years. Students from all years were not good at stopping non-indicated care. The educative process has been unable to prepare approximately half of the students	Students generally made appropriate clinical choices for when to treat, especially for contraindications, especially when there were obvious pathological findings. These skills were more apparent in the higher years of study. However, the concept of non-indication may not have been as well understood and did not differ between the years	Performance Processes	Safety of Clinical Processes Health Worker Education, Skills, and Safety
Informed consent	Jamison 1998 (Australia)	To explore, by means of a collective case study, informed consent as practiced in Australian chiropractic practice	Qualitative descriptive research	Practice observation suggested that chiropractors did obtain informal but seldom secured formally structured consent to examine and treat a patient. This was integrated into total patient management, where it served both to inform patients about their conditions and the proposed intervention and to involve patients in decision-making. There was a marked reluctance to initiate discussion about the potentially serious side effects of chiropractic adjustment	In this study, chiropractors tended to subordinate the legal imperative to provide complete consent to the moral imperative of helping the patient make the best decision. Sensitivity to individuals as patients and people may prove to be chiropractors' best strategy for providing good health care and avoiding litigation	Performance Processes	Patient and Family Engagement
Informed consent	Langworthy 2005 (United Kingdom)	To investigate approaches to consent among a small (n = 150) sample of practicing UK chiropractors	Cross sectional study	UK practitioners demonstrate inconsistency and non-compliance with many informed consent components, including discussing AEs and obtaining consent prior to treatment. This represents a serious breach of both ethical and legal responsibility for these practitioners. Most practitioners surveyed felt they had not received adequate guidance in informed consent	Valid informed consent is somewhat poorly understood or implemented by members of the UK chiropractic profession. An increased awareness of the need to obtain valid consent has not been matched by sufficient support and guidance from UK chiropractic statutory, professional, or educational bodies	Performance Processes	Policies to Eliminate Avoidable Harm in Healthcare Patient and Family Engagement Health Worker Education, Skills, and Safety
Informed consent	Langworthy 2007 (United States and United Kingdom)	To investigate attitudes toward and implementation of consent procedures in a sample of UK and US chiropractors and how well these practices satisfy the core ethical principles of autonomy, veracity, justice, nonmaleficence, and beneficence	Cross sectional study	Barriers and difficulties exist among US/UK chiropractors when discussing minor and major risks associated with treatment and obtaining informed consent. Awareness of legal or professional indemnity requirements around informed consent varied among respondents, but the majority perceived the process as both part of treatment and a legal protective mechanism for themselves	These suggest that a patient's autonomy and right to self-determination may be diminished when seeking chiropractic care. Our findings further suggest that problems may also be experienced by the chiropractor in relation to the principles of justice (equality) and veracity (openness) and highlight the potential for conflict between beneficence and paternalism	Performance Processes Values	Patient and Family Engagement Health Worker Education, Skills, and Safety
Informed consent	Langworthy 2010 (United Kingdom)	To investigate the reality of risk disclosure and consequent withdrawal from manipulative treatment and to obtain insight into the attitudes of chiropractors towards informed consent and disclosure	Cross sectional study	88% considered the explanation of risk associated with treatment important, but only 45% reported they always discuss this with patients, 41% reported they sometimes do, and 5% said they never do. 46% believed disclosing risk could increase patient anxiety to the extent they withdraw from treatment, 79% believed chiros have a moral/ethical obligation to disclose risk with cervical SMT, 80% believe they have a moral/ethical obligation to disclose risk with cervical SMT despite concerns of patient withdrawal from treatment (but only 45% report they always do so)	Fears about increased patient anxiety leading to withdrawal from care as a direct consequence of the disclosure of risks associated with cervical manipulation may be unfounded. Inconsistency and non-compliance with the valid informed consent process remain a feature in some areas of UK chiropractic practice, despite acknowledgment of moral and ethical responsibility	Performance Processes Values	Patient and Family Engagement Health Worker Education, Skills, and Safety
Informed consent	Dagenais 2012 (United States)	To propose questions that may be helpful to educate patients considering treatment approaches to manage their LBP so that they may participate in shared and fully informed decision making	Qualitative descriptive research	There is a growing realization that patients with LBP would benefit greatly from a clinician who cannot only offer some of the interventions recommended by clinical practice guidelines (CPGs) but also give advice on the indications, benefits, harms, and costs of all other common treatment options. Chiropractors are well-positioned to do this. The informed consent process should ideally be centered on a clinician and patient discussion to reduce the knowledge gap and increase their ability to make an informed decision	Informed consent is a tool that can be used to achieve shared decision-making before initiating a treatment regimen. An ideal informed consent process includes information on the condition being treated, the nature and purpose of the intervention, its expected benefits, harms, and available alternatives. If doctors of chiropractic are to assume a more substantial role in spine care, they will be expected to provide information to help patients make informed decisions about their health, even if this discussion results in the choice of an intervention other than SMT	Performance Processes	Patient and Family Engagement Health Worker Education, Skills, and Safety
Informed consent	Winterbottom 2015 (Canada)	To explore chiropractic patients' perceptions of exchanging risk information during informed consent and compare them with the legal perspective of the informed consent process	Qualitative descriptive research	Participants perceived informed consent as a social process consisting of ongoing information exchange with their practitioners and informed by interactions with family, friends, and the media. They described a process that consisted of four stages where risk information was incorporated into their decision-making: (1) preconceived ideas about safety/risk; (2) perceived practitioner competence; (3) risk discussion & consent form; and (4) patient-practitioner feedback loop. These contextual factors influenced participants' perceptions of risk and informed their decisions to receive treatment	These findings suggest that patients perceive informed consent as a process rather than a static event and that educating chiropractic patients about the risks associated with treatment while satisfying the legal requirements of informed consent is possible. However, the informed consent form does not appear to be the most appropriate tool for patient education and may be more useful as a legal waiver	Performance Processes Values	Patient and Family Engagement
Reporting and learning systems	Thiel 2006 (United Kingdom)	To design and test a reporting format for patient safety incidents (PSIs) related to chiropractic practice	Observational study	The rate of return of incident reports by field chiropractors was disappointing, as only eight reporting forms were received from seven individual chiropractors. For Anglo-European Chiropractic College (AECC) interns, 225 PSIs were reported for 19,1008 patient contacts. Either misuse of therapeutic equipment (32%) or the treatment intervention itself (31%) were most frequently reported in association with the occurrence of a PSI. In 64% of the PSIs, the students felt no harm had occurred to the patient. A likely relationship between suspected cause and incident was thought to have existed in 80% of the cases	These results demonstrate the importance of implementing the attributes of a safe and informed culture within the chiropractic profession by introducing patient safety incident reporting systems. A reporting system will provide an important opportunity to learn from the experiences of one patient or chiropractor and reduce the risk of something similar happening to others	Performance Processes Values	High Reliability Systems Safety of Clinical Processes Information, Research, and Risk Management
Reporting and learning systems	Gunn 2008 (United Kingdom)	To identify levels of awareness and understanding of a patient safety incident (PSI) reporting system by members of the British Chiropractic Association (BCA), their attitudes to reporting PSIs, and barriers to incident reporting and use of the Chiropractic Reporting and Learning System (CRLS) by BCA members	Qualitative descriptive research	Uncertainty and misunderstandings exist among practitioners concerning the CRLS—considerable education of the chiropractic profession in needed regarding the purpose and potential outcomes of reporting PSIs. Suggestions include providing regular information and feedback, a network system of chiropractors who actively promote a culture of safety and the use of the CRLS, and greater clarity and examples of what to report for the different categories on the CRLS form	More needs to be done to quell the fears and uncertainty of these chiropractors and improve patient safety incident reporting to the CRLS. The chiropractors interviewed for this study may have had poor awareness and understanding of the CRLS, which may have contributed to its scant use. Without the ongoing utilization of a reporting system, further development of safer clinical practice could be difficult and impossible	Performance Processes Values	High Reliability Systems Information, Research, and Risk Management
Reporting and learning systems	Pohlman 2014 (Canada)	To describe the development and validation of provider and patient measurement instruments to identify potential SMT AE in provider offices	Instrument development study	A feasible instrument was developed and tested that measured seriousness, causality, preventability, and patient disposition when assessing AEs. The reporting system should be active rather than passive, and reports should go directly to a third party to remove patient fears of reporting. Patient perspective is important because providers have demonstrated poor reporting of suspect AEs	The development and validation of instruments to evaluate SMT AEs may benefit SMT research by providing the opportunity for rigorous prospective assessment of potential SMT-related AEs and their risk factors. Future efforts with these instruments include putting them into providers' offices for use on consecutive patients to assess AE after SMT	Performance Processes	Patient and Family Engagement Information, Research, and Risk Management
Reporting and learning systems	Pohlman 2016b (Canada)	To describe factors that may inhibit pediatric chiropractors' participation in a patient safety reporting and learning system	Cross sectional study	Barriers to participating in a reporting system include time pressure, patient-related concerns, fear of blame, and feeling it was unnecessary. Patients who have participated in a pilot SMT reporting system reported that instead of developing a negative impression of their provider, they were pleased that their provider was willing to participate in a study looking directly at patient safety	Ensuring patient safety is part of their regulatory mandate for self-regulated professions, including chiropractic. This survey has identified potential barriers to participation in a reporting and learning system for the pediatric chiropractic profession, with the largest barriers identified being time pressure and the potential for patient concerns	Processes Values	Information, Research, and Risk Management
Reporting and learning systems	Rozmovits 2016 (Canada)	To gain insight into the current safety culture around the use of SMT by regulated health professionals in Canada and to explore perceptions of readiness for implementing formal mechanisms for tracking associated AEs	Qualitative descriptive research	Inter- and intra-professional disagreements concerning SMT safety were evident with chiropractors, physiotherapists, and naturopaths. While participants broadly welcomed the prospect of having better data to support better practice, the perceived barriers to implementing an incident reporting system for SMT were plentiful, including practical barriers linked to SMT delivery's unbounded and multi-professional nature	The established approaches to patient safety derived from high-risk industry and commonly used in acute hospital settings are difficult to apply to non-medical primary care. Collaboration across professions on models that allow practitioners to share information anonymously and help practitioners learn from the reported incidents is needed	Performance Processes Values	Safety of Clinical Processes Information, Research, and Risk Management Synergy, Partnership, and Solidarity
Reporting and learning systems	Pohlman 2020a (Canada and United States)	To compare the quantity and quality of AE reports after chiropractic manual therapy in children less than 14 years of age, using active versus passive surveillance reporting systems	RCT	Monitoring of AEs after treatment needs to occur to make healthcare safer. Practitioners and patients/caregivers need to know the safety profile of the treatments they are considering to make informed decisions about treatment options and set appropriate expectations. Active surveillance is more effective in identifying AEs and can be successfully conducted in ambulatory care settings. Barriers to implementation include time and resources. The key to implementing is patient involvement	Active surveillance collected more AE reports than passive surveillance (8.8% vs 0.1%). It is more effective in identifying AEs than passive surveillance and may provide more accurate estimates of AE incidence, including serious AEs, which is necessary when appraising overall risk	Performance	Safety of Clinical Processes Patient and Family Engagement Information, Research, and Risk Management
Reporting and learning systems	Pohlman 2020b (United States)	To assess the feasibility of implementing an active-surveillance reporting system of AEs within a chiropractic teaching clinic using paper-based data collection and to determine the preliminary frequency of AEs after a chiropractic clinical encounter administered by chiropractic interns	Observational study	Barriers to using active surveillance reporting system in teaching clinics include paper forms, extra work for students, added time to patient visits, and lack of intern adherence to the study protocol regarding symptom severity. Facilitators included the opportunity for students to be involved in research and practical opportunities to talk openly about patient safety. Ten percent of patients in this study had an AE, with 80% of these reported by patients	We found that it is feasible to implement the collection of patient safety data at a chiropractic teaching clinic using an active surveillance reporting system. Patient safety research must continue within this population to enhance informed consent and potential mitigation options. Active surveillance reporting systems can generate high-quality AE data, allowing for the identification of potential risk factors and the exploration of mitigation options in future studies	Performance Processes	Safety of Clinical Processes Information, Research, and Risk Management
Reporting and learning systems	Thomas 2023 (United Kingdom)	To analyze the safety incidents submitted to CPiRLS over a 10-year period (2009 to 2019) in order to enhance patient safety	Cross sectional study	268 safety incidents were reported to the chiropractic patient incident reporting and learning system (CPiRLS) over the ten-year period, with an average 30.5% increase over whole years (2010–2018), demonstrating an upward trend over time. Patients should be adequately informed about the currently established risks associated with manual therapy to ensure informed consent is gained and shared decision-making can occur	This detailed review of ten years of patient safety incidents reported by the chiropractic profession demonstrates that, while significant under-reporting is highly suspected, there has been an upward trend in the frequency of safety incidents reported to CPiRLS during the period from 2009 to 2019, providing a sizeable database for helpful analysis. Patient harm was reported in 30% of safety incidents. However, the level of harm is unclear. CPiRLS has an important role in improving patient safety in the chiropractic profession	Performance Processes	Safety of Clinical Processes Information, Research, and Risk Management
Reporting and learning systems	Pohlman 2024	To report the incidence of AE after SMT, collected using the SafetyNet Active Surveillance Reporting System	Observational study	The study found an overall adverse event (AE) incidence of 21.3% per patient visit, decreasing to 6.3% in patients with no prior symptoms. Most AEs were mild or moderate and commonly involved worsening pre-existing symptoms like pain and stiffness, while serious events, such as dizziness requiring hospitalization, were rare. The findings highlight the need for standardized AE reporting systems, enhanced data collection through electronic health records, and community-based surveillance to improve safety in chiropractic and physiotherapy care	This study found the incidence of AEs following chiropractic or physiotherapy patient encounters to be 21.3%. Of these AE reports, the severity classifications were noted as: mild (7.9%), moderate (6.2%), severe (3.7%), serious (1.5%), and missing severity responses (2.0%). This study provides information for clinicians and patients and serves as a framework to more fully understand post-visits AEs more fully and potential strategies to mitigate them	Performance	Safety of Clinical Processes Patient and Family Engagement Information, Research, and Risk Management Synergy, Partnership, and Solidarity
Office sanitization	Pokras 1990 (United States)	To examine the efficiency of using paper as a physical barrier to prevent infection arising from contamination of the adjusting table headrests	Observational study	The clean headrest paper has a negligible bacterial count and a statistically significant elevation in bacterial colony count following patient contact. There was no statistically significant difference in the colony density from swabs collected from the headrest before and after patient contact; thus, the paper covering the headrest must have acted as an acceptable physical barrier to bacterial transmission between the patient and the headrest	Clean headrest paper acts as an adequate physical barrier to protect the sanitary status of the headrest surface. However, this paper barrier may not be adequate for protecting the total body surface of the supine patient in terms of the potential for the paper to tear and the limited area that is covered around the headrest	Performance Processes	Safety of Clinical Processes
Office sanitization	Bifero 2006 (United States)	To enumerate the microbial flora on the headrest, armrest, and thoracic portion of chiropractic adjusting tables to determine the presence of pathogenic microorganisms and identify the potential for nosocomial transmission	Cross sectional study	Chiropractic table surfaces that come into skin contact with patients harbor many organisms, including coagulase-positive staphylococci, gram-negative bacilli, and MRSA. Non-pathogenic environmental fungi were also found, but not enough to warrant significant overgrowth. Headrests and armrests had a higher percentage of organisms compared to the thoracic portion, supporting the conclusion that skin contact was the main source	All of the surfaces sampled on the chiropractic adjusting tables carried microorganisms. This study supports and emphasizes the need for an effective disinfection protocol to prevent bacterial and fungal buildup that may pose a direct threat to the patient and possibly the community	Performance Processes	Safety of Clinical Processes
Office sanitization	Evans 2007a (United States)	To make an initial assessment of chiropractic students' attitudes, current behaviors, and practices regarding hand washing, hand sanitizing, and disinfection of treatment tables in one chiropractic college	Qualitative descriptive research	68.8% of students wash their hands frequently, 28% carry hand sanitizer, 95% always change face paper, but 80% never/rarely wipe off the table with sanitizer. They support hand washing and sanitizing tables but are concerned about the safety of agents, the recommended cleaning frequency, and negative patient perceptions. Without a disinfectant, students will most likely continue to do nothing to treatment table surfaces unless a protocol is established	This chiropractic teaching institution needs to adopt reasonable infection control measures, including sinks in common areas, hand sanitizer dispensers, and more education for students and faculty. Table disinfection should be adopted as a routine procedure	Performance Processes	Safety of Clinical Processes Synergy, Partnership, and Solidarity
Office sanitization	Evans 2007b (United States)	To assess the presence of pathogenic microbes on treatment tables in one outpatient teaching clinic and determine a simple behavioral model for infection control including table disinfection and accepted hand washing and sanitizing protocols	Observational study	Two treatment tables contained (g-) organisms, and all tables contained at least some (g +) organisms, including S. epidermidis, S. saprophyticus, and S. aureus. Post-sanitizing testing demonstrated no pathogenic microbes present on tested tables after the use of either of the disinfecting agents. It is proposed that treatment table surfaces be sanitized at the start of the day, mid-day, at the close of the day and any time clinical judgment warrants additional disinfecting	A systematic infection control protocol may not be in place for the chiropractic profession, but it is needed. Future research should determine the relative risk associated with treatment on tables where inadequate infection control measures are an issue. Studies that quantify the amount of pathogenic microbes on treatment surfaces should be considered as risk may hinge on quantity	Performance Processes	Safety of Clinical Processes
Office sanitization	Evans 2008 (United States)	To assess the presence of microbes and other allergens or pathogens on cloth chiropractic tables	Observational study	A sampling of tables found mold spores, candida colonies, and gram-positive bacteria, but no MRSA. The infection control program should include the need to wash hands or sanitize them between every patient contact, wipe tables with a suitable disinfection agent between every patient, and consider gloved hands anytime these appear clinically indicated. Other protocols, such as offering workers mandated sick time when sick and appropriate vaccine options, may also be needed	Pathogens and allergens are present on cloth chiropractic treatment tables and benches. Currently, the chiropractic profession in the United States does not have a suggested guideline that has been adopted for disinfecting treatment tables. Teaching institutions need comprehensive infection control programs, which should subsequently be shared with field practitioners	Performance Processes	Safety of Clinical Processes
Office sanitization	Evans 2009 (United States)	To present a proposed guideline for hand and treatment table surface sanitizing for the chiropractic profession that is evidence-based and can easily be adopted by teaching institutions and doctors in the field	Narrative review	Compliance with hand hygiene and table disinfection is poor, with reasons given as time factors, lack of hand sanitizer, lack of sinks near treatment areas, or even failure to understand when there is an appropriate moment for hand hygiene outside the most obvious opportunities. The lack of chiropractic-specific guidelines on hand hygiene and treatment table disinfection needs to be addressed with future research testing education and compliance programs in the profession	Microbes that are potentially harmful to both doctor and patient are known to be present on chiropractic treatment tables. In the absence of any standardized hand and table sanitizing protocols, it is suggested that hand sanitizing with alcohol-based gel be recommended, appropriate wipes or solutions should be used for table sanitizing, cloth tables should be abandoned, and a national guideline on hand sanitizing be adopted	Performance Processes	Policies to Eliminate Avoidable Harm in Healthcare Safety of Clinical Processes Synergy, Partnership, and Solidarity
General patient safety topics	Gleberzon 2011 (Canada)	To perform a narrative review of the chiropractic literature regarding older patients between 2001 and 2010	Narrative review	SMT can be safely provided for older patients. Older patients do not experience more injuries than younger patients; they may actually experience fewer due to technique and force modification by the practitioner or greater joint stiffness. Modifications recommended include non-HVLA techniques, increased surface area contact, alternate positioning for adjustments, and using drop pieces	Modifications can be made to increase patient safety when considering chiropractic care for older patients	Performance	Safety of Clinical Processes
General patient safety topics	Boucher 2014 (Canada)	To expand practitioners' knowledge on areas of liability when treating low back pain patients	Case series	Failures that led to a verdict of negligence have included informed consent, diagnosis and choice or application of manipulative technique. The chiropractor must obtain informed consent and include a discussion with the patient, including advising them of the risks of SMT and other alternative treatments. The chiropractor must constantly re-evaluate their diagnosis as the patient progresses and document this progress and their counseling on a procedure. Case law has established that a conservative course of treatment should be established for the first 2–3 days before commencing SMT	According to case law, SMT conducted to the thoracic spine, lumbar spine, or sacroiliac joints constitutes a risk of aggravating a pre-existent disc injury. With acute nonspecific low back pain patients, practitioners should consider disc herniation as a differential diagnosis when risk factors are documented in the patient's history, despite the absence of objective neurological signs during physical examination	Performance Processes	Safety of Clinical Processes
General patient safety topics	Jevne 2014 (Norway)	To describe claims reported to the Danish Patient Compensation Association and the Norwegian System of Compensation to Patients related to chiropractic from 2004 to 2012	Observational study	The most frequent complaint categories were 91 (30.3%) cases of worsening symptoms following treatment, 57 (19%) cases of alleged disk herniations, and 46 (15.3%) cases of delayed referral. Many complaints were filed because of unrealistic expectations of treatment effects or because the clinicians did not inform the patients about commonly occurring benign reactions to treatment, suggesting these may be preventable if adequate information is given prior to treatment	Clinicians need to stay vigilant and prioritize thorough and reasoned clinical examinations. They should also devote time to explaining the large incidence of minor AEs to patients and emphasizing that these are not AEs but normal, benign reactions to manual treatment. We strongly support the implementation of incident reporting in chiropractic practice, as more knowledge can be gained through a more systematic collection of reported AEs	Performance Processes	Safety of Clinical Processes Information, Research, and Risk Management
General patient safety topics	Innes 2016 (Australia and France)	To review CCE definitions of competence, domains of educational competencies, components of the domains of competencies as represented by assessment and diagnosis, ethics, and intellectual development, and to make recommendations, if significant deficiencies were found	Systematic/ scoping review	The Council on Chiropractic Education (CCE) should consider the evidence for a more prescriptive approach to physical examination components to reduce the possibility of errors. An internationally uniform definition of competence for chiropractic education and assessment is required, and identifying opportunities for improving the enforcement of standards may result in a uniform quality international standard of patient care and practice safety	The main similarities between international CCEs were found in relation to the structure and terms describing the domain level of competencies. Differences were noted in the interpretation of those terms. Recommendations were made, and adopting these could create homogenized, internationally consistent, and high-quality graduating standards	Performance Processes	Policies to Eliminate Avoidable Harm in Healthcare Health Worker Education, Skills, and Safety Synergy, Partnership, and Solidarity
General patient safety topics	Marchand 2015b (Italy)	To identify the amount of force necessary to create damage in the pediatric spine, which could be used as a limit of force that should never be exceeded during pediatric SMT. Adult data were identified to compare with pediatric data to evaluate differences between pediatric and adult spines so that scaling models could be proposed. A model of care is discussed to address the gap of knowledge concerning pediatric SMT technique adaptations to prevent the occurrence of safety incidents	Qualitative descriptive research	Results showed a nonlinear increase in the cervical tensile strength in relation to the increasing age of the specimens. The progressive increase in forces used during pediatric SMT and technique adaptations progressively changing according to age reflect the biomechanical findings of increased tensile strength in relation to specimen age. The reference of 50 newtons (N) of force may help quantify the limit of the sub-catastrophic clinical level, help practitioners remain within the paraphysiologic zone during pediatric SMT, and prevent the occurrence of safety incidents related to incorrect technique	This review suggests a nonlinear increase in the tensile strength of the cervical spine according to the age of human specimens. Based on the reported tensile strengths, a preliminary model of care combining the scaling ratios and the reported technique adaptations used during pediatric SMT has been proposed. The safety and clinical implications of the preliminary model of care may impact the practice of SMT for infants and children	Performance Processes	Safety of Clinical Processes

LEGEND: AE—adverse event; CONSORT—Consolidated Standards of Reporting Trials; MRSA—Methicillin-resistant Staphylococcus aureus; RCT—Randomized Clinical Trial; SMT—spinal manipulation therapy; UK—United Kingdom; US—United States of America

### Thematic analysis

**Fig. 2:**
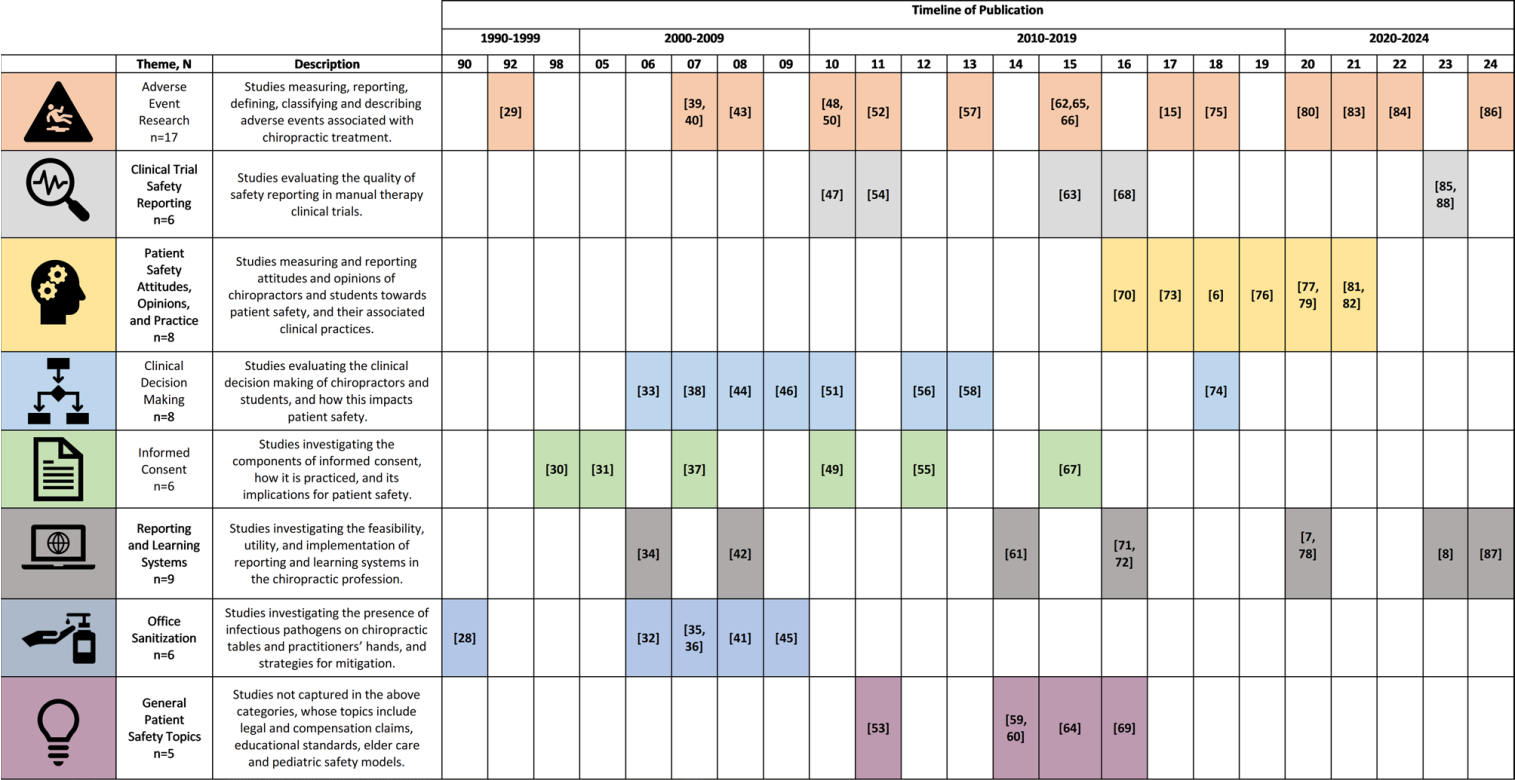
Study references organized by patient safety culture themes and publication timeline

Figure 2 presents the thematic results by the number of studies and years of publication, in which 8 patient safety culture themes were generated: *Adverse Event Research* (n = 17), *Clinical Trial Safety Reporting* (n = 6), *Patient Safety Attitudes, Opinions, and Practice* (n = 8), *Clinical Decision Making* (n = 8), *Informed Consent* (n = 6), *Reporting and Learning Systems* (n = 9), *Office Sanitization* (n = 6), and *General Patient Safety Topics* (n = 5). Below is a summary of the key findings for each theme.

#### Theme 1: Adverse event research (n = 17)

Seventeen studies (1992 – 2024) examined the measurement, reporting, classification, and description of adverse events following chiropractic treatment [15, 29, 39, 40, 43, 48, 50, 52, 57, 62, 65, 66, 75, 80, 83, 84, 86]. While serious adverse events were rare, benign and transient adverse events were common, often attributable to nonspecific effects [15, 39, 57, 83]. The included studies highlight ongoing challenges in standardizing adverse event terminology and classification [48, 52, 84]. Recent research has built upon earlier findings, exploring outcomes in diverse settings, including pediatric and teaching clinics [80, 86].

#### Theme 2: Clinical trial safety reporting (n = 6)

Six studies (2010–2023) evaluated adverse event and safety reporting in manual therapy clinical trials [47, 54, 63, 68, 85, 89] revealing inconsistencies and inadequacies, a challenge not unique to the chiropractic field [88, 89]. Findings reinforced the need for standardized adverse event reporting in both clinical and research settings [63, 85]. While the 2010 Consolidated Standards of Reporting Trials (CONSORT) guideline included a basic reporting statement [47, 54, 68], a 2022 extension introduced a more structured approach [89]. The rise in publications after 2010 could indicate an initial attempt to evaluate whether the research community began following these guidelines [90].

#### Theme 3: Patient safety attitudes, opinions, and practice (n = 8)

Eight studies (2016–2021) explored chiropractors’ and students’ perspectives on patient safety and their clinical practices [6, 70, 73, 76, 77, 79, 81, 82], many led by the interprofessional, international SafetyNET team [6, 7, 61, 70, 71, 78, 79, 82, 87, 91]. Findings highlight the importance of strengthening patient safety culture by addressing training gaps [6, 73, 79, 82]. Recent trends emphasize evaluating safety dimensions—performance, processes, and values [92]—rather than solely documenting harm incidence.

#### Theme 4: Clinical decision making (n = 8)

Eight studies (2006–2018) investigated chiropractic clinical decision making with an emphasis on patient safety [33, 38, 44, 46, 51, 56, 58, 74]. Findings suggest that refined clinical assessment skills—such as appropriate referral, triage, and re-evaluation—enhance safety [33, 38, 58]. Most studies were published between 2006 and 2013, with limited exploration in the past decade.

#### Theme 5: Informed consent (n = 6)

Six studies (1998–2015) analyzed the components of informed consent, its practice, and implications for patient safety [30, 31, 37, 49, 55, 67]. Research, particularly active between 2007 and 2015, revealed inconsistencies and non-compliance in informed consent processes [30, 31, 49]; however, research in this area has not been updated in recent years.

#### Theme 6: Reporting and learning systems (n = 9)

Nine studies (2006–2024) explored chiropractic reporting and learning systems [7, 8, 34, 42, 61, 71, 72, 78, 87], highlighting low awareness and uptake of CPiRLS [8, 42]. While CPiRLS has expanded in Europe, use of comparable systems is lacking in the United States, Canada, Australia, and New Zealand. Similarly, assessment of chiropractic care related safety data is absent from healthcare systems offering chiropractic services with more general patient safety incident reporting and learning systems. Active surveillance is more effective than passive surveillance in adverse event detection, though passive surveillance remains valuable for broad coverage, cost-effectiveness, and early signal detection [7–9, 71, 78, 87]. Research in this area has grown since 2008.

#### Theme 7: Office sanitization (n = 6)

Six studies (1990–2009) examined microbial contamination on chiropractic tables and practitioners’ hands, highlighting the need for systematic disinfection protocols [28, 32, 35, 36, 41, 45]. Despite its relevance, office sanitization research was absent in recent years, including during the COVID-19 pandemic.

#### Theme 8: General patient safety topics (n = 5)

Five studies (2011–2016) emphasized the need for age-specific treatment modifications [53, 64], improved communication and differential diagnosis to better mitigate legal claims [59, 60], and the need for educational competencies to enhance patient safety [69].

### Framework mapping

**Fig. 3:**
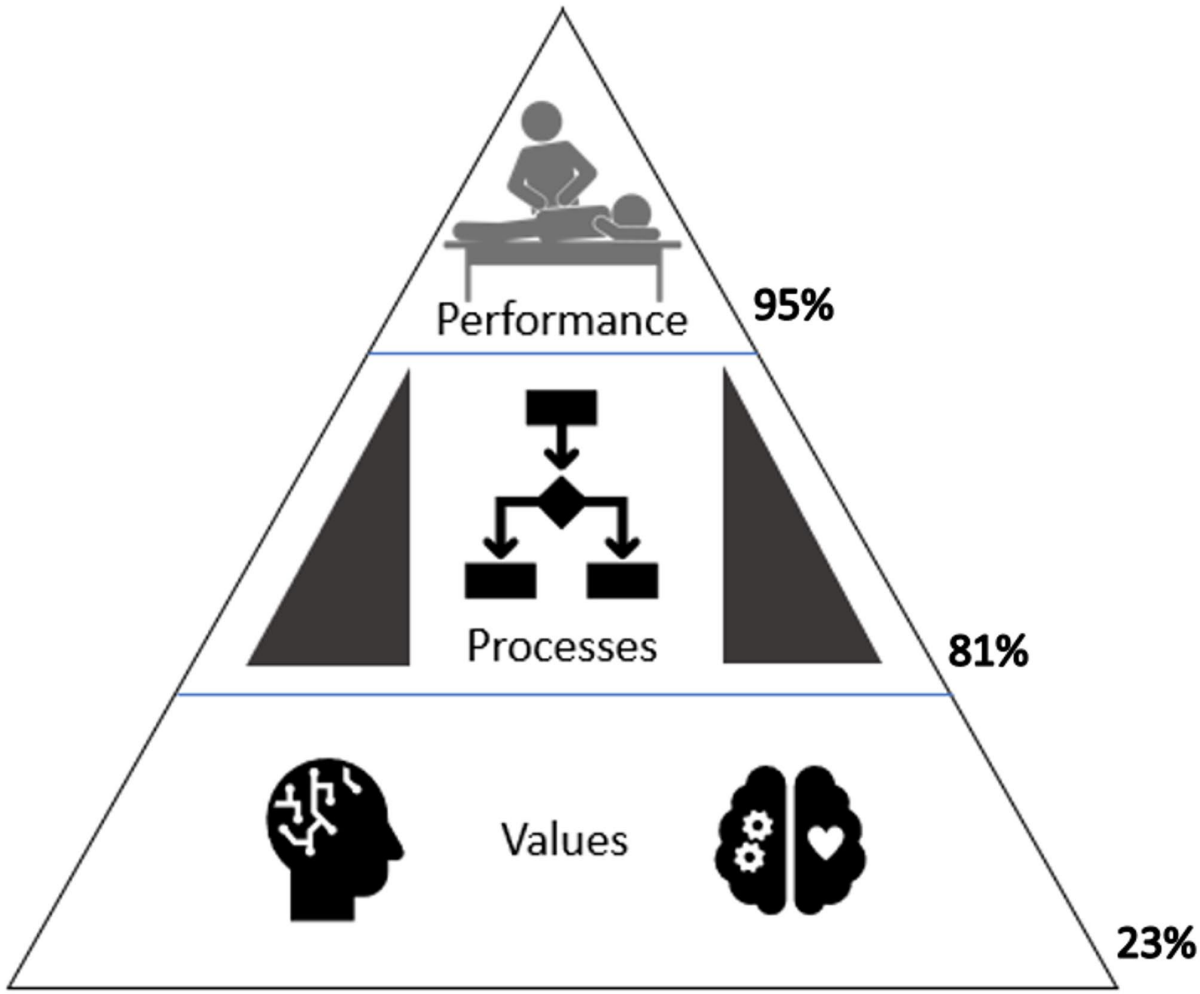
The patient safety culture pyramid [9] and reported percentages (see Table 1 for individual study categorization)

**Fig. 4:**
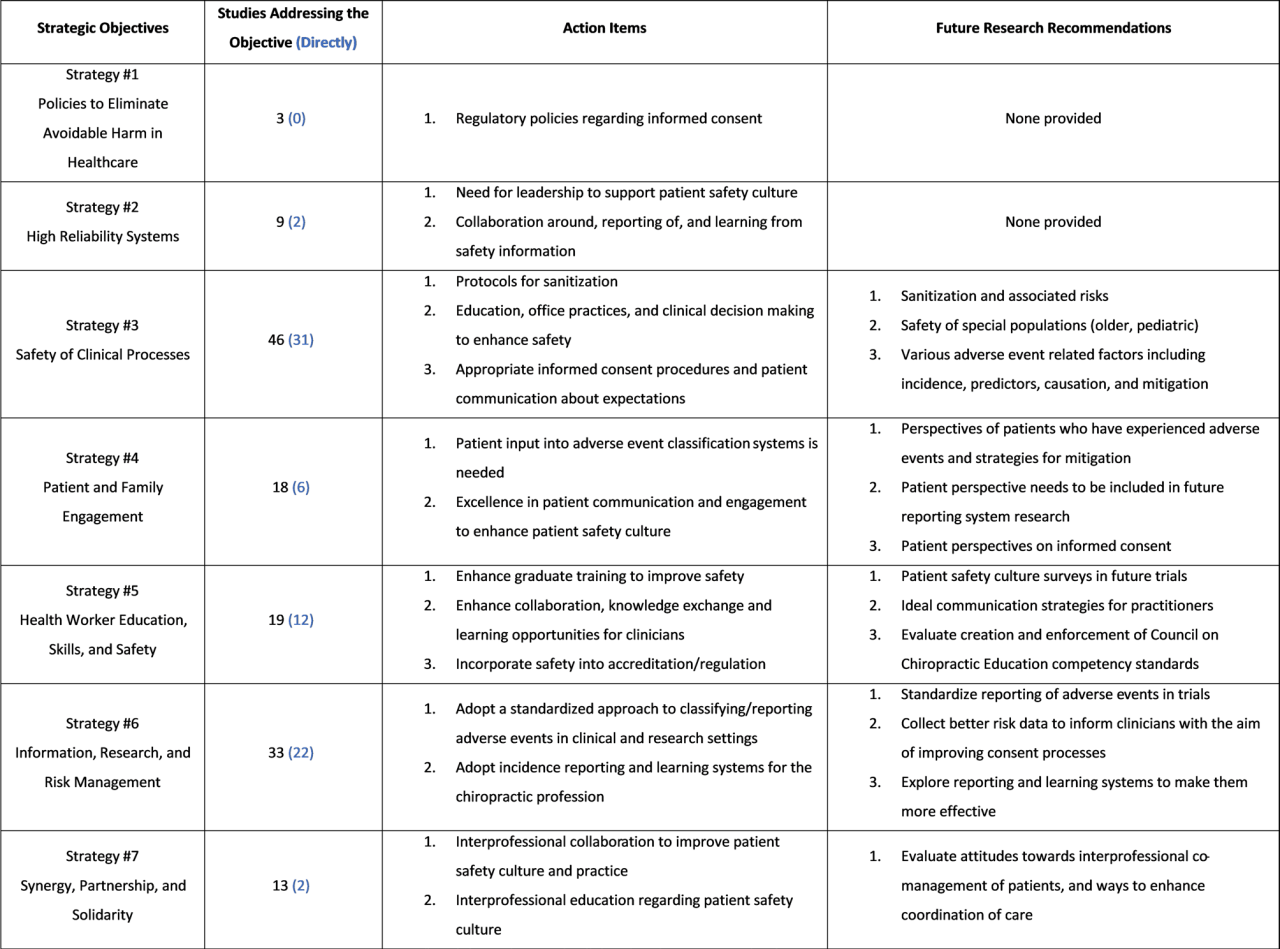
Mapping of studies to WHO GPSAP strategic objectives

Figures 3 and 4 illustrate key trends in chiropractic patient safety research mapped to relevant patient safety frameworks. Figure 3 maps studies onto the Patient Safety Culture Pyramid, showing that most research addressed multiple safety culture levels: 95% examined performance, 81% addressed processes, and only 23% explored core safety values—a critical gap because values shape attitudes, behaviors, and decision-making [9]. To strengthen safety culture, recommendations include integrating safety climate surveys into research initiatives and establishing a patient safety culture database for spinal manipulation therapy providers, enabling long term quality improvement [6, 77, 81].

Figure 4 compares the included studies with the WHO *Global Patient Safety Action Plan* [1] while highlighting research gaps. While many studies (n = 46) focused on clinical safety processes, far fewer examined policy (n = 3), high reliability systems (n = 9), or interprofessional collaboration (n = 13). Few studies directly addressed patient safety policies, though some indirectly recommended improvements in sanitization, informed consent, and competency standards [31, 45, 69]. Strengthening policy efforts is essential to advance chiropractic patient safety culture and align with the WHO goal of zero avoidable harm in healthcare.

Research on high reliability systems emphasized leadership in safety culture [7, 34] and intra-organizational collaboration in safety reporting [82], but key areas – human factors, ergonomics, and governance – remain unaddressed. Similarly, studies on synergy and partnerships highlighted interprofessional collaboration [33, 51, 72, 82, 84] and education [40, 73, 76] but lacked focus on developing global patient safety networks. Patient engagement also was underrepresented. Only one-third of the studies engaging patients involved direct patient input, with most discussing engagement conceptually. Patient perspectives were examined in adverse event definitions [52], adverse event mitigation [77], and informed consent [67]. Future research should prioritize direct patient involvement to strengthen patient safety culture across all levels [70, 80].

### Consultation

Finally, for Stage 6 – Consultation, the research team received feedback from key partner representatives regarding the comprehensiveness of the scoping review (see Additional File 4 for all results). Suggestions were primarily related to semantic changes for clarity, and included adding a specific example of the current inconsistency in adverse event definitions. Additional comments highlighted important perspectives on topics related to patient safety (e.g., safety risk in special populations, health system complexities, safety measurement criteria) but were outside of the primary focus of this review on patient safety culture.

## Discussion

This scoping review identified 65 articles that collectively outline the breadth of patient safety culture research in chiropractic while revealing critical gaps in policy development, high reliability systems, interprofessional collaboration, and direct patient engagement. Most of the research centered on performance (95%) and processes (81%), with core safety values (23%) remaining underexplored. Strengthening safety climate assessments, regulatory policies, and patient engagement strategies is necessary to foster a more robust and evidence-based patient safety culture in chiropractic.

The findings indicate that the chiropractic profession's patient safety culture is evolving, with the identified themes highlighting both advancements and ongoing challenges. The frequency of benign adverse events associated with chiropractic treatment underscores the need for standardized reporting and terminology [48, 52, 84]. Despite efforts to enhance harm reporting through guidelines like the CONSORT, inconsistencies persist in clinical trials [88]. Informed consent remains another critical yet underexplored aspect of patient safety, with past research revealing significant variability and non-compliance in its implementation across different settings [55, 67]. The decline in recent studies on informed consent, particularly as new methods of delivering, discussing, and documenting consent are being developed, suggests a gap in understanding how to ensure patients receive clear, consistent, and comprehensive information about potential risks and benefits of chiropractic care.

Emerging research increasingly emphasizes patient safety culture dimensions —performance, processes, and values—shifting the focus from documenting harms to driving proactive improvements [6, 91]. Despite this progress, research on patient safety education and training remains limited within the chiropractic profession. Strengthening interprofessional collaboration, enhancing safety-focused curricula at both the undergraduate and postgraduate levels, and fostering a culture of shared responsibility were identified as essential next steps in advancing patient safety within the chiropractic profession.

To align chiropractic patient safety research with global health priorities, future studies should expand beyond adverse event reporting and address patient-centered safety culture, as outlined in the WHO *Global Patient Safety Action Plan* [1], and the WFC Global Patient Safety Initiative’s “Call to Action” [10]. Several key areas require further investigation.

### Regulatory frameworks and policy development

There is a need for regulatory policies and competency standards addressing standardized informed consent, surveillance systems for adverse events, and patient safety training. Policymakers should prioritize developing and adopting structured reporting and learning systems that support high reliability systems and cross-disciplinary collaboration, as recommended by the WHO *Global Patient Safety Action Plan* [7, 8, 34, 42, 55, 61, 67, 87].

### Standardizing adverse event reporting and safety data collection

The absence of standardized adverse event definitions and classification systems remains a major obstacle to effective reporting and meta-analysis in chiropractic research [42, 49, 52, 63, 68, 84, 85, 88]. While adverse events dominate patient safety discussions in professional discourses and social media posts alike, future efforts should prioritize establishing consistent terminology and reporting structures to enhance data accuracy and comparability. In addition to the widespread inconsistency in terminology used to report adverse events, further complications arise from variability in the populations studied—a large proportion of research focuses on pediatric patients [40, 43, 63, 70, 71], while significantly less attention given to older populations [53, 76], limiting the generalizability of findings across age groups. Additionally, foregrounding patient perspectives in adverse event definitions, reporting structures, and mitigation strategies will ensure a more patient-centered approach. Expanding and systematically integrating safety values assessments into chiropractic research and practice is essential for fostering a stronger patient safety culture [4, 6, 9].

### Enhancing patient engagement in safety research

Although patient engagement is a critical component of WHO *Global Patient Safety Action Plan* [1], few chiropractic studies have directly involved patients in safety discussions or decision-making processes. Patient engagement refers to the meaningful involvement of patients and their families as equal partners in all aspects of health care—ranging from bedside decisions to national policy—by ensuring their voices, experiences, and rights are integrated into governance, strategy, safety reporting, and care delivery, with full transparency, access to information, and opportunities to influence and co-lead improvements in patient safety [1]. Engaging patients in safety initiatives enhances transparency, improves communication, and reduces preventable harm [93, 94]. Future research should actively include patients in defining safety priorities, reporting experiences, and shaping care improvements [52, 67, 70, 77].

### Building high reliability systems and interprofessional collaboration

Chiropractic lacks well-established and broadly adopted high reliability systems—critical for minimizing errors and ensuring continuous learning from safety incidents [95]. Future research should explore how safety culture is influenced by practitioner approaches and practice settings, in addition to governance structures, leadership involvement, and interdisciplinary collaboration. Integrating patient safety research across professions could yield synergistic solutions, benefiting both chiropractic and the broader healthcare community [96, 97]. This presents an opportunity for future research to analyze patient safety data from healthcare systems that have integrated chiropractic care, such as the United States Veterans Health Administration [98, 99]. Comparing this data with other health professions can provide valuable insights. Additionally, studies to examine the impact of system dynamics and operational workflows in chiropractic clinics within high reliability organizations on patient safety outcomes can offer a model for chiropractic practice across diverse healthcare settings [100].

While chiropractors play a critical role in patient safety, there is a disconnect between clinical practice, education, and research in addressing system-wide safety concerns. Research must move beyond documenting individual safety events and include the development and evaluation of interventions that enhance safety culture. This could include the advancement of the use of CPiRLS, or similar reporting systems on a global scale. The CPiRLS system is a voluntary, anonymous reporting tool for documenting adverse events, near misses, and safety concerns in chiropractic care, aimed at promoting a culture of learning. However, its effectiveness is limited by challenges such as underreporting and low engagement, highlighting the need for improved reporting methods and greater education on patient safety [8, 42].

Expanding active surveillance initiatives will improve adverse event detection, risk mitigation, and safety policy development. Active surveillance initiatives use structured methods—such as follow-up interviews, electronic monitoring, and direct data collection—to more accurately identify and report adverse events in healthcare. These systems generate standardized, reliable data and detect significantly more AEs than passive reporting [78]. However, they are resource-intensive, requiring greater time, cost, and clinician involvement, and face similar barriers to engagement as passive systems, including time constraints and fear of blame [7, 78]. While underutilized, especially in ambulatory care, active surveillance is recognized as a valuable tool for improving patient safety.

Chiropractic education programs should also integrate competency-based patient safety training, ensuring that future practitioners are equipped with the skills to proactively prevent and manage safety risks and incidents. Lastly, research on synergy and partnerships is underdeveloped, despite the WHO emphasis on collaborative patient safety efforts. Future research should explore how chiropractic can contribute to global patient safety networks and participate in interdisciplinary safety initiatives. Additionally, meta-analyses and longitudinal studies evaluating the effectiveness of patient safety interventions on chiropractic are essential to drive evidence-based improvements.

### Near misses

Many studies excluded during the screening process were “near misses” – relevant to patient safety but lacking explicit discussion of patient safety culture. Research on regulatory complaints [101, 102], culturally sensitive care [103], and interprofessional referral patterns [104] provided valuable insights, but did not specifically analyze or discuss study results through a patient safety culture lens. Additionally, chiropractic best practices and clinical guidelines (see Additional File 2 for the 14 identified best practice and clinical practice guidelines excluded in this review), often overlook patient safety implications, warranting future investigation. Future research should systematically analyze case reports, clinical guidelines, and best practice recommendations to assess their impact on patient safety.

## Strengths and limitations

This review followed a rigorous, stepwise methodology, ensuring comprehensive data extraction and analysis [11, 12], with the protocol pre-registered on OSF. An international, interprofessional research team contributed expertise in scoping reviews and patient safety, while a medical librarian oversaw the search strategy, including a PRESS peer-review [21], enhancing study reliability. We did not include EMBASE in our search, nor grey literature, meaning potentially relevant studies may have been omitted; however, we used backward citation searching as an additional step in order to minimize missing studies. Additionally, some studies presented ambiguous patient safety information, offering indirect rather than explicit findings on safety culture, which may have affected the assessment of relevance and strength. Lastly, the inclusion of only English-language publications limits generalizability, potentially overlooking important studies from non-English speaking regions. This said, only 2 studies were excluded for non-English language of publication, suggesting that patient safety culture research may be limited outside the geographic regions identified in our analysis.

## Conclusion

This scoping review highlights the breadth of patient safety culture research in chiropractic while identifying key gaps in adverse event reporting, informed consent, and incident reporting systems. Despite progress in adverse event research and clinical safety, challenges remain, including inconsistent reporting, lack of standardized terminology, and limited patient engagement. To align with global safety standards, future research should focus on regulatory frameworks, standardized adverse event reporting, patient engagement, and high reliability systems. Strengthening safety values in chiropractic practice and education is essential for fostering a sustainable patient safety culture. By maintaining a focus on continuous advancements in chiropractic safety research, the profession can enhance transparency, accountability, and alignment with WHO *Global Patient Safety Action Plan* priorities while ensuring safer and more effective chiropractic care.

## Supplementary Information

Supplementary Material 1

Supplementary Material 2

Supplementary Material 3

Supplementary Material 4

## Data Availability

The datasets used and/or analysed during the current study are housed in the Parker University Data Repository (https://my.parker.edu/ICS/Research/Research_Data_Repository.jnz) and are available from the corresponding author on reasonable request.

## Abbreviations

WHO: World Health Organization
WFC: World Federation of Chiropractic
PRISMA-SCR: Preferred Reporting Items Systematic Reviews and Meta-Analyses for Scoping Reviews
OSF: Open Science Framework
PRESS: Peer Review of Electronic Search Strategies
MeSH: Medical Subject Headings
CAA: Chiropractic Association of Alberta
RCC: Royal College of Chiropractors
CPiRLS: Chiropractic Patient Incident Reporting and Learning System
RCT: Randomized Clinical Trial
CONSORT: Consolidated Standards of Reporting Trials

## Declarations

### Awards

This manuscript was awarded the NCMIC-JMPT Research Award at the 18th World Federation of Chiropractic Biennial Congress in Copenhagen, Denmark.
