# Supplementary material for: Patient safety culture research within the chiropractic profession: a scoping review
Source: Chiropr Man Therap. 2025 Oct 21;33:46. doi: 10.1186/s12998-025-00605-z (PMC12538883; doi:10.1186/s12998-025-00605-z)
Supplement: Supplementary file 1 — Supplementary Material 1 [file 12998_2025_605_MOESM1_ESM.docx]

**Additional File 1 – Search Strategies for all Databases**

## **MEDLINE (OVID)**

Original Search: Ovid MEDLINE(R) and Epub Ahead of Print, In-Process, In-Data-Review & Other Non-Indexed Citations, Daily and Versions <1946 to March 15, 2024>

1 Manipulation, Chiropractic/ OR Chiropractic/ 4414

2 Manipulation, Spinal/ 1850

3 Musculoskeletal Manipulations/ 2302

4 Manipulation, Orthopedic/ OR manipulation, osteopathic/ OR Kinesiology, Applied/ 5495

5 chiropr*.af. 11052

6 (manual adj3 therap*).ab,kf,kw,ti. 4593

7 (spin* adj3 manipul*).ab,kf,kw,ti. 3683

8 (cervical adj3 manipul*).ab,kf,kw,ti. 806

9 (thoracic adj3 manipul*).ab,kf,kw,ti. 254

10 (lumbar adj3 manipul*).ab,kf,kw,ti. 200

11 (mobili* adj techni*).ab,kf,kw,ti. 535

12 (manipul* adj3 therap*).ab,kf,kw,ti. 4861

13 (spin* adj3 adjust*).ab,kf,kw,ti. 723

14 (cervical adj3 adjust*).ab,kf,kw,ti. 355

15 (lumbar adj3 adjust*).ab,kf,kw,ti. 198

16 (thoracic adj3 adjust*).ab,kf,kw,ti. 78

17 OR/1-16 28548

18 exp Patients/ 86190

19 (patient* OR client* OR inpatient* OR outpatient*).ab,kf,kw,ti. 8596992

20 18 OR 19 8613431

21 Safety Management/ OR Safety/ 63317

22 Patient Safety/ 25988

23 accidents/ OR accident prevention/ OR accidental falls/ 56071

24 exp Medical Errors/ 122747

25 (safe* OR accident* OR fall OR falls OR err* OR mistake* OR adverse OR harm*).ab,kf,kw,ti. 2693586

26 Patient Harm/ 231

27 adverse effects.fx. 2044306

28 exp risk/ 1407305

29 OR/21-28 5388252

30 20 and 29 2392431

31 Culture/ 34332

32 organizational culture/ 19117

33 "Attitude of Health Personnel"/ OR Attitude/ 186234

34 Health Knowledge, Attitudes, Practice/ 128199

35 crew resource management, healthcare/ OR learning health system/ OR patient care team/ 70163

36 (teamwork OR rounds).ab,hw,kf,ti. 46797

37 (learn* adj5 cultur*).ab,hw,kf,ti. 3473

38 (learn* adj5 organiz*).ab,hw,kf,ti. 4068

39 (learn* adj5 organis*).ab,hw,kf,ti. 1289

40 (safe* adj3 cultur*).ab,kf,kw,ti. 6706

41 (safe* adj3 attitud*).ab,kf,kw,ti. 1825

42 (safe* adj3 climat*).ab,kf,kw,ti. 1753

43 (safe* adj3 environ*).ab,kf,kw,ti. 14759

44 (safe* adj3 belie*).ab,kf,kw,ti. 1140

45 (safe* adj3 practic*).ab,kf,kw,ti. 16240

46 (safe* adj3 behavior*).ab,kf,kw,ti. 3887

47 (safe* adj3 behaviour*).ab,kf,kw,ti. 1346

48 (safe* adj3 perform*).ab,kf,kw,ti. 28216

49 (safe* adj3 polic*).ab,kf,kw,ti. 2694

50 (safe* adj3 procedur*).ab,kf,kw,ti. 31974

51 OR/31-50 549835

52 17 and 30 and 51 168

Search Update: Ovid MEDLINE(R) and Epub Ahead of Print, In-Process, In-Data-Review & Other Non-Indexed Citations, Daily and Versions <1946 to December 13, 2024>

1 Manipulation, Chiropractic/ OR Chiropractic/ 4463

2 Manipulation, Spinal/ 1894

3 Musculoskeletal Manipulations/ 2395

4 Manipulation, Orthopedic/ OR manipulation, osteopathic/ OR Kinesiology, Applied/ 5545

5 chiropr*.af. 11376

6 (manual adj3 therap*).ab,kf,kw,ti. 4907

7 (spin* adj3 manipul*).ab,kf,kw,ti. 3858

8 (cervical adj3 manipul*).ab,kf,kw,ti. 825

9 (thoracic adj3 manipul*).ab,kf,kw,ti. 269

10 (lumbar adj3 manipul*).ab,kf,kw,ti. 212

11 (mobili* adj techni*).ab,kf,kw,ti. 578

12 (manipul* adj3 therap*).ab,kf,kw,ti. 5028

13 (spin* adj3 adjust*).ab,kf,kw,ti. 762

14 (cervical adj3 adjust*).ab,kf,kw,ti. 375

15 (lumbar adj3 adjust*).ab,kf,kw,ti. 204

16 (thoracic adj3 adjust*).ab,kf,kw,ti. 79

17 OR/1-16 29575

18 exp Patients/ 87947

19 (patient* OR client* OR inpatient* OR outpatient*).ab,kf,kw,ti. 8929478

20 18 OR 19 8945977

21 Safety Management/ OR Safety/ 63852

22 Patient Safety/ 26925

23 accidents/ OR accident prevention/ OR accidental falls/ 57047

24 exp Medical Errors/ 124550

25 (safe* OR accident* OR fall OR falls OR err* OR mistake* OR adverse OR harm*).ab,kf,kw,ti. 2839510

26 Patient Harm/ 241

27 adverse effects.fx. 2092059

28 exp risk/ 1448864

29 OR/21-28 5591949

30 20 and 29 2487670

31 Culture/ 34590

32 organizational culture/ 19563

33 "Attitude of Health Personnel"/ OR Attitude/ 190597

34 Health Knowledge, Attitudes, Practice/ 133392

35 crew resource management, healthcare/ OR learning health system/ OR patient care team/ 71466

36 (teamwork OR rounds).ab,hw,kf,ti. 49715

37 (learn* adj5 cultur*).ab,hw,kf,ti. 3715

38 (learn* adj5 organiz*).ab,hw,kf,ti. 4274

39 (learn* adj5 organis*).ab,hw,kf,ti. 1350

40 (safe* adj3 cultur*).ab,kf,kw,ti. 7265

41 (safe* adj3 attitud*).ab,kf,kw,ti. 1941

42 (safe* adj3 climat*).ab,kf,kw,ti. 1863

43 (safe* adj3 environ*).ab,kf,kw,ti. 16344

44 (safe* adj3 belie*).ab,kf,kw,ti. 1206

45 (safe* adj3 practic*).ab,kf,kw,ti. 17163

46 (safe* adj3 behavior*).ab,kf,kw,ti. 4106

47 (safe* adj3 behaviour*).ab,kf,kw,ti. 1420

48 (safe* adj3 perform*).ab,kf,kw,ti. 29445

49 (safe* adj3 polic*).ab,kf,kw,ti. 2893

50 (safe* adj3 procedur*).ab,kf,kw,ti. 33136

51 OR/31-50 568868

52 17 and 30 and 51 175

53 limit 52 to dt=20240315-20241213 8

## **CINAHL Complete (EBSCO)**

Original Search: Friday, April 12, 2024

S1 (MH "Manipulation, Chiropractic") OR (MH "Chiropractic") OR (MH "Chiropractic Assessment") OR (MH "Chiropractic Practice") OR (MH "Chiropractors") 29,933

S2 (MH "Manual Therapy") OR (MH "Applied Kinesiology") OR (MH "Manipulation, Osteopathic") OR (MH "Manipulation, Orthopedic") 10,265

S3 chiropr* 34,228

S4 TI "manual therapy" OR AB "manual therapy" 2,687

S5 TI spin* W3 manipul* OR AB spin* W3 manipul* 2,126

S6 TI cervical* W3 manipul* OR AB cervical* W3 manipul* 481

S7 TI thoracic* W3 manipul* OR AB thoracic* W3 manipul* 213

S8 TI lumbar* W3 manipul* OR AB lumbar* W3 manipul* 127

S9 TI mobili* W3 techni* OR AB mobili* W3 techni* 558

S10 TI manipul* W3 therap* OR AB manipul* W3 therap* 1,505

S11 TI spin* W3 adjust* OR AB spin* W3 adjust* 263

S12 TI cervical W3 adjust* OR AB cervical W3 adjust* 155

S13 TI lumbar W3 adjust* OR AB lumbar W3 adjust* 69

S14 TI thoracic W3 adjust* OR AB thoracic W3 adjust* 41

S15 (S1 OR S2 OR S3 OR S4 OR S5 OR S6 OR S7 OR S8 OR S9 OR S10 OR S11 OR S12 OR S13 OR S14) 45,520

S16 (MH "Patients+") OR (MH "Patient Care+") 1,165,672

S17 TI ( patient* OR client* OR inpatient* OR outpatient* ) AND AB ( patient* OR client* OR inpatient* OR outpatient* ) 465,789

S18 S16 OR S17 1,490,395

S19 (MH "Safety+") OR (MH "Harm Reduction") OR (MH "Accidents") OR (MH "Accidental Falls") OR (MH "Risk Management") 266,257

S20 TI ( safe* OR accident* OR fall OR falls OR err* OR mistake* OR adverse OR harm* ) OR AB ( safe* OR accident* OR fall OR falls OR err* OR mistake* OR adverse OR harm* ) 701,214

S21 S19 OR S20 837,032

S22 S18 AND S21 205,351

S23 (MH "Culture") OR (MH "Cultural Values") OR (MH "Organizational Culture+") OR (MH "Attitude") OR (MH "Attitude of Health Personnel") OR (MH "Management of Labor") OR (MH "Learning Health System") OR (MH "Teamwork") 158,991

S24 TI (teamwork OR rounds) OR AB (teamwork OR rounds) 33,138

S25 TI learn* W5 cultur* OR AB learn* W5 cultur* 1,497

S26 TI learn* W5 organiz* OR AB learn* W5 organiz* 1,096

S27 TI learn* W5 organis* OR AB learn* W5 organis* 383

S28 TI safe* W5 cultur* OR AB safe* W5 cultur* 3,041

S29 TI safe* W5 attitud* OR AB safe* W5 attitud* 848

S30 TI safe* W5 climat* OR AB safe* W5 climat* 984

S31 TI safe* W5 environ* OR AB safe* W5 environ* 5,072

S32 TI safe* W5 belie* OR AB safe* W5 belie* 386

S33 TI safe* W5 practic* OR AB safe* W5 practic* 8,875

S34 TI safe* W5 behav* OR AB safe* W5 behav* 2,624

S35 TI safe* W5 perform* OR AB safe* W5 perform* 5,005

S36 TI safe* W5 polic* OR AB safe* W5 polic* 1,470

S37 TI safe* W5 proced* OR AB safe* W5 proced* 6,409

S38 S23 OR S24 OR S25 OR S26 OR S27 OR S28 OR S29 OR S30 OR S31 OR S32 OR S33 OR S34 OR S35 OR S36 OR S37 215,979

S39 S15 AND S22 AND S38 51

Search Update: Mon, December 16, 2024

S1 (MH "Manipulation, Chiropractic") OR (MH "Chiropractic") OR (MH "Chiropractic Assessment") OR (MH "Chiropractic Practice") OR (MH "Chiropractors")

S2 (MH "Manual Therapy") OR (MH "Applied Kinesiology") OR (MH "Manipulation, Osteopathic") OR (MH "Manipulation, Orthopedic")

S3 chiropr*

S4 TI "manual therapy" OR AB "manual therapy"

S5 TI spin* W3 manipul* OR AB spin* W3 manipul*

S6 TI cervical* W3 manipul* OR AB cervical* W3 manipul*

S7 TI thoracic* W3 manipul* OR AB thoracic* W3 manipul*

S8 TI lumbar* W3 manipul* OR AB lumbar* W3 manipul*

S9 TI mobili* W3 techni* OR AB mobili* W3 techni* 579

S10 TI manipul* W3 therap* OR AB manipul* W3 therap* 1,520

S11 TI spin* W3 adjust* OR AB spin* W3 adjust* 258

S12 TI cervical W3 adjust* OR AB cervical W3 adjust* 147

S13 TI lumbar W3 adjust* OR AB lumbar W3 adjust* 67

S14 TI thoracic W3 adjust* OR AB thoracic W3 adjust* 39

S15 S1 OR S2 OR S3 OR S4 OR S5 OR S6 OR S7 OR S8 OR S9 OR S10 OR S11 OR S12 OR S13 OR S14) 46,033

S16 (MH "Patients+") OR (MH "Patient Care+") 1,199,434

S17 TI ( patient* OR client* OR inpatient* OR outpatient* ) AND AB ( patient* OR client* OR inpatient* OR outpatient* ) 474,175

S18 S16 OR S17 1,527,599

S19 (MH "Safety+") OR (MH "Harm Reduction") OR (MH "Accidents") OR (MH "Accidental Falls") OR (MH "Risk Management") 275,022

S20 TI ( safe* OR accident* OR fall OR falls OR err* OR mistake* OR adverse OR harm* ) OR AB ( safe* OR accident* OR fall OR falls OR err* OR mistake* OR adverse OR harm* ) 717,614

S21 S19 OR S20 857,538

S22 S18 AND S21 212,618

S23 (MH "Culture") OR (MH "Cultural Values") OR (MH "Organizational Culture+") OR (MH "Attitude") OR (MH "Attitude of Health Personnel") OR (MH "Management of Labor") OR (MH "Learning Health System") OR (MH "Teamwork") 163,016

S24 TI (teamwork OR rounds) OR AB (teamwork OR rounds) 34,175

S25 TI learn* W5 cultur* OR AB learn* W5 cultur* 1,562

S26 TI learn* W5 organiz* OR AB learn* W5 organiz* 1,109

S27 TI learn* W5 organis* OR AB learn* W5 organis* 390

S28 TI safe* W5 cultur* OR AB safe* W5 cultur* 3,205

S29 TI safe* W5 attitud* OR AB safe* W5 attitud* 886

S30 TI safe* W5 climat* OR AB safe* W5 climat* 1,008

S31 TI safe* W5 environ* OR AB safe* W5 environ* 5,303

S32 TI safe* W5 belie* OR AB safe* W5 belie* 395

S33 TI safe* W5 practic* OR AB safe* W5 practic* 9,178

S34 TI safe* W5 behav* OR AB safe* W5 behav* 2,662

S35 TI safe* W5 perform* OR AB safe* W5 perform* 5,015

S36 TI safe* W5 polic* OR AB safe* W5 polic* 1,507

S37 TI safe* W5 proced* OR AB safe* W5 proced* 6,486

S38 S23 OR S24 OR S25 OR S26 OR S27 OR S28 OR S29 OR S30 OR S31 OR S32 OR S33 OR S34 OR S35 OR S36 OR S37 221,541

S39 S15 AND S22 AND S38 55

S40 ( S15 AND S22 AND S38) AND EM 20240315-20241213 4

## **AMED (EBSCO)**

Original Search: Friday, April 12, 2024

S1 (ZU "chiropractic") OR (ZU "manipulation") OR (ZU "manipulation chiropractic") OR (ZU "manipulation orthopedic") OR (ZU "manipulation osteopathic") OR (ZU "manipulative therapies") OR (ZU "musculoskeletal manipulation") OR (ZU "musculoskeletal manipulations") 9,901

S2 chiropr* 10,230

S3 TI manual n3 therap* OR AB manual n3 therap* OR KW manual n3 therap* 1,199

S4 TI spin* N3 manipul* OR AB spin* N3 manipul* OR KW spin* N3 manipul* 1,387

S5 TI cervical N3 manipul* OR AB cervical N3 manipul* OR KW cervical N3 manipul* 326

S6 TI thoracic N3 manipul* OR AB thoracic N3 manipul* OR KW thoracic N3 manipul* 111

S7 TI lumbar N3 manipul* OR AB lumbar N3 manipul* OR KW lumbar N3 manipul* 83

S8 TI mobili* N3 techni* OR AB mobili* N3 techni* OR KW mobili* N3 techni* 227

S9 TI manipul* N3 therap* OR AB manipul* N3 therap* OR KW manipul* N3 therap* 756

S10 TI spin* N3 adjust* OR AB spin* N3 adjust* OR KW spin* N3 adjust* 169

S11 TI cervical N3 adjust* OR AB cervical N3 adjust* OR KW cervical N3 adjust* 50

S12 TI lumbar N3 adjust* OR AB lumbar N3 adjust* OR KW lumbar N3 adjust* 20

S13 TI thoracic N3 adjust* OR AB thoracic N3 adjust* OR KW thoracic N3 adjust* 21

S14 S1 OR S2 OR S3 OR S4 OR S5 OR S6 OR S7 OR S8 OR S9 OR S10 OR S11 OR S12 OR S13 13,762

S15 (ZU "patient advocacy") OR (ZU "patient care") OR (ZU "patient centered care") OR (ZU "patients") OR (ZU "inpatients") OR (ZU "outpatients") 5,026

S16 TI ( patient* OR client* OR inpatient* OR outpatient* ) OR AB ( patient* OR client* OR inpatient* OR outpatient* ) OR KW (patient* OR client* OR inpatient* OR outpatient* ) 100,304

S17 S15 OR S16 100,774

S18 (ZU "safety") OR (ZU "patient safety") 2,095

S19 (ZU "accident prevention") OR (ZU "accidental falls") OR (ZU "accidents") OR (ZU "patient harm") OR (ZU "medical errors") OR (ZU "adverse effects") OR (ZU "risk") 12,130

S20 TI ( safe* OR accident* OR fall OR falls OR err* OR mistake* OR adverse OR harm* ) AND AB ( safe* OR accident* OR fall OR falls OR err* OR mistake* OR adverse OR harm* ) AND KW ( safe* OR accident* OR fall OR falls OR err* OR mistake* OR adverse OR harm* ) 1,410

S21 S18 OR S19 OR S20 13,725

S22 S17 AND S21 4,482

S23 (ZU "culture") OR (ZU "organizational culture") OR (ZU "attitude") OR (ZU "attitude of health personnel") 7,764

S24 TX ( teamwork OR rounds ) OR AB ( teamwork OR rounds ) OR KW ( teamwork OR rounds ) 936

S25 TX learn* N5 cultur* OR AB learn* N5 cultur* OR KW learn* N5 cultur* 72

S26 TI learn* N5 organiz* OR AB learn* N5 organiz* OR KW learn* N5 organiz* 62

S27 TI learn* N5 organis* OR AB learn* N5 organis* OR KW learn* N5 organis* 23

S28 TI safe* N3 cultur* OR AB safe* N3 cultur* OR KW safe* N3 cultur* 52

S29 TI safe* N3 attitud* OR AB safe* N3 attitud* OR KW safe* N3 attitud* 10

S30 TI safe* N3 climat* OR AB safe* N3 climat* OR KW safe* N3 climat* 18

S31 TI safe* N3 environ* OR AB safe* N3 environ* OR KW safe* N3 environ* 148

S32 TI safe* N3 belie* OR AB safe* N3 belie* OR KW safe* N3 belie* 22

S33 TI safe* N3 practic* OR AB safe* N3 practic* OR KW safe* N3 practic* 199

S34 TI safe* N3 behavio* OR AB safe* N3 behavio* OR KW safe* N3 behavio* 65

S35 TI safe* N3 perform* OR AB safe* N3 perform* OR KW safe* N3 perform* 204

S36 TI safe* N3 polic* OR AB safe* N3 polic* OR KW safe* N3 polic* 11

S37 TI safe* N3 procedur* OR AB safe* N3 procedur* OR KW safe* N3 procedur* 152

S38 S23 OR S24 OR S25 OR S26 OR S27 OR S28 OR S29 OR S30 OR S31 OR S32 OR S33 OR S34 OR S35 OR S36 OR S37 9,520

S39 S14 AND S22 AND S38 11

Search Update: Mon, December 16, 2024

No new results.

## **Index to Chiropractic Literature**

Original Search: Friday, April 12, 2024

| **Search #** | **Query** | **Items found** | **Date & Time** |
| --- | --- | --- | --- |
| [S1](https://www.chiroindex.org/?action=set&setId=10245220) | Subject:\"Patients\" OR Subject:\"Patient Care\" OR All Fields:patient OR All Fields:patients OR All Fields:client OR All Fields:clients OR All Fields:inpatient OR All Fields:inpatients OR All Fields:outpatient OR All Fields:outpatients | 6171 | 2024-04-12 14:03:27 |
| [S2](https://www.chiroindex.org/?action=set&setId=10245228) | Subject:\"Safety\" OR Subject:\"Patient Safety\" OR Subject:\"Safety Management\" OR Subject:\"Accidents\" OR Subject:\"Accidental Falls\" OR Subject:\"Accident Prevention\" OR Subject:\"Medical Errors\" OR Subject:\"Risk\" OR Subject:\"Chiropractic / adverse effects\" | 433 | 2024-04-12 14:07:09 |
| [S3](https://www.chiroindex.org/?action=set&setId=10245239) | All Fields:safe OR All Fields:safety OR All Fields:accident OR All Fields:accidents OR All Fields:accidental OR All Fields:fall OR All Fields:falls OR All Fields:mistake OR All Fields:mistakes OR All Fields:mistaken OR All Fields:adverse OR All Fields:harm OR All Fields:harms OR All Fields:harmful OR All Fields:error OR All Fields:errors | 1683 | 2024-04-12 14:10:17 |
| [S4](https://www.chiroindex.org/?action=set&setId=10245241) | Subject:\"Safety\" OR Subject:\"Patient Safety\" OR Subject:\"Safety Management\" OR Subject:\"Accidents\" OR Subject:\"Accidental Falls\" OR Subject:\"Accident Prevention\" OR Subject:\"Medical Errors\" OR Subject:\"Risk\" OR Subject:\"Chiropractic / adverse effects\" OR All Fields:safe OR All Fields:safety OR All Fields:accident OR All Fields:accidents OR All Fields:accidental OR All Fields:fall OR All Fields:falls OR All Fields:mistake OR All Fields:mistakes OR All Fields:mistaken OR All Fields:adverse OR All Fields:harm OR All Fields:harms OR All Fields:harmful OR All Fields:error OR All Fields:errors | 1780 | 2024-04-12 14:10:45 |
| [S5](https://www.chiroindex.org/?action=set&setId=10245245) | Subject:\"Patients\" OR Subject:\"Patient Care\" OR All Fields:patient OR All Fields:patients OR All Fields:client OR All Fields:clients OR All Fields:inpatient OR All Fields:inpatients OR All Fields:outpatient OR All Fields:outpatients AND Subject:\"Safety\" OR Subject:\"Patient Safety\" OR Subject:\"Safety Management\" OR Subject:\"Accidents\" OR Subject:\"Accidental Falls\" OR Subject:\"Accident Prevention\" OR Subject:\"Medical Errors\" OR Subject:\"Risk\" OR Subject:\"Chiropractic / adverse effects\" OR All Fields:safe OR All Fields:safety OR All Fields:accident OR All Fields:accidents OR All Fields:accidental OR All Fields:fall OR All Fields:falls OR All Fields:mistake OR All Fields:mistakes OR All Fields:mistaken OR All Fields:adverse OR All Fields:harm OR All Fields:harms OR All Fields:harmful OR All Fields:error OR All Fields:errors | 826 | 2024-04-12 14:11:31 |
| [S6](https://www.chiroindex.org/?action=set&setId=10245256) | Subject:\"Culture\" OR Subject:\"Organizational Culture\" OR Subject:\"Attitude\" OR Subject:\"Attitude of Health Personnel\" OR Subject:\"Health Knowledge, Attitudes, Practice\" OR Subject:\"Patient Care Team\" | 160 | 2024-04-12 14:15:31 |
| [S7](https://www.chiroindex.org/?action=set&setId=10245285) | All Fields:teamwork OR All Fields:rounds OR All Fields:learning AND organization OR All Fields:learning AND culture OR All Fields:safety AND culture OR All Fields:safety AND climate OR All Fields:safety AND environment OR All Fields:safety AND beliefs OR All Fields:safety AND performance OR All Fields:safety AND practices OR All Fields:safety AND behaviors OR All Fields:safety AND behaviours OR All Fields:safety AND policy OR All Fields:safety AND procedure | 182 | 2024-04-12 14:28:37 |
| [S8](https://www.chiroindex.org/?action=set&setId=10245286) | Subject:\"Culture\" OR Subject:\"Organizational Culture\" OR Subject:\"Attitude\" OR Subject:\"Attitude of Health Personnel\" OR Subject:\"Health Knowledge, Attitudes, Practice\" OR Subject:\"Patient Care Team\" OR All Fields:teamwork OR All Fields:rounds OR All Fields:learning AND organization OR All Fields:learning AND culture OR All Fields:safety AND culture OR All Fields:safety AND climate OR All Fields:safety AND environment OR All Fields:safety AND beliefs OR All Fields:safety AND performance OR All Fields:safety AND practices OR All Fields:safety AND behaviors OR All Fields:safety AND behaviours OR All Fields:safety AND policy OR All Fields:safety AND procedure | 333 | 2024-04-12 14:30:05 |
| [S9](https://www.chiroindex.org/?action=set&setId=10245289) | Subject:\"Patients\" OR Subject:\"Patient Care\" OR All Fields:patient OR All Fields:patients OR All Fields:client OR All Fields:clients OR All Fields:inpatient OR All Fields:inpatients OR All Fields:outpatient OR All Fields:outpatients AND Subject:\"Safety\" OR Subject:\"Patient Safety\" OR Subject:\"Safety Management\" OR Subject:\"Accidents\" OR Subject:\"Accidental Falls\" OR Subject:\"Accident Prevention\" OR Subject:\"Medical Errors\" OR Subject:\"Risk\" OR Subject:\"Chiropractic / adverse effects\" OR All Fields:safe OR All Fields:safety OR All Fields:accident OR All Fields:accidents OR All Fields:accidental OR All Fields:fall OR All Fields:falls OR All Fields:mistake OR All Fields:mistakes OR All Fields:mistaken OR All Fields:adverse OR All Fields:harm OR All Fields:harms OR All Fields:harmful OR All Fields:error OR All Fields:errors AND Subject:\"Culture\" OR Subject:\"Organizational Culture\" OR Subject:\"Attitude\" OR Subject:\"Attitude of Health Personnel\" OR Subject:\"Health Knowledge, Attitudes, Practice\" OR Subject:\"Patient Care Team\" OR All Fields:teamwork OR All Fields:rounds OR All Fields:learning AND organization OR All Fields:learning AND culture OR All Fields:safety AND culture OR All Fields:safety AND climate OR All Fields:safety AND environment OR All Fields:safety AND beliefs OR All Fields:safety AND performance OR All Fields:safety AND practices OR All Fields:safety AND behaviors OR All Fields:safety AND behaviours OR All Fields:safety AND policy OR All Fields:safety AND procedure | 74 | 2024-04-12 14:30:42 |

Search Update: Mon, December 16, 2024

|  | Search # | Query | Items found | Date & Time |
| --- | --- | --- | --- | --- |
|  | [S1](https://chiroindex.org/?action=set&setId=11514539) | Subject:\"Patients\" OR Subject:\"Patient Care\" OR All Fields:patient OR All Fields:patients OR All Fields:client OR All Fields:clients OR All Fields:inpatient OR All Fields:inpatients OR All Fields:outpatient OR All Fields:outpatients, Year: from 2024 to 2024 | 111 | 2024-12-13 09:22:00 |
|  | [S2](https://chiroindex.org/?action=set&setId=11514598) | Subject:\"Safety\" OR Subject:\"Safety Management\" OR Subject:\"Patient Safety\" OR Subject:\"Accidents\" OR Subject:\"Accidental Falls\" OR Subject:\"Accident Prevention\" OR Subject:\"Medical Errors\" OR Subject:\"Risk\" OR Subject:\"Chiropractic / adverse effects\", Year: from 2024 to 2024 | 3 | 2024-12-13 09:50:28 |
|  | [S3](https://chiroindex.org/?action=set&setId=11514638) | All Fields:safe OR All Fields:safety OR All Fields:accident OR All Fields:accidents OR All Fields:accidental OR All Fields:fall OR All Fields:falls OR All Fields:mistake OR All Fields:mistakes OR All Fields:mistaken OR All Fields:adverse OR All Fields:harm OR All Fields:harms OR All Fields:harmful OR All Fields:error OR All Fields:errors, Year: from 2024 to 2024 | 26 | 2024-12-13 09:55:00 |
|  | [S4](https://chiroindex.org/?action=set&setId=11514765) | Subject:\"Safety\" OR Subject:\"Safety Management\" OR Subject:\"Patient Safety\" OR Subject:\"Accidents\" OR Subject:\"Accidental Falls\" OR Subject:\"Accident Prevention\" OR Subject:\"Medical Errors\" OR Subject:\"Risk\" OR Subject:\"Chiropractic / adverse effects\", Year: from 2024 to 2024 OR All Fields:safe OR All Fields:safety OR All Fields:accident OR All Fields:accidents OR All Fields:accidental OR All Fields:fall OR All Fields:falls OR All Fields:mistake OR All Fields:mistakes OR All Fields:mistaken OR All Fields:adverse OR All Fields:harm OR All Fields:harms OR All Fields:harmful OR All Fields:error OR All Fields:errors, Year: from 2024 to 2024 | 26 | 2024-12-13 10:25:54 |
|  | [S5](https://chiroindex.org/?action=set&setId=11515402) | Subject:\"Culture\" OR Subject:\"Organizational Culture\" OR Subject:\"Attitude\" OR Subject:\"Attitude of Health Personnel\" OR Subject:\"Health Knowledge, Attitudes, Practice\" OR Subject:\"Patient Care Team\", Year: from 2024 to 2024 | 2 | 2024-12-13 13:29:58 |
|  | [S6](https://chiroindex.org/?action=set&setId=11515472) | All Fields:teamwork OR All Fields:rounds OR All Fields:learning AND organization OR All Fields:learning AND culture OR All Fields:safety AND culture OR All Fields:safety AND climate OR All Fields:safety AND environment OR All Fields:safety AND beliefs OR All Fields:safety AND performance OR All Fields:safety AND practices OR All Fields:safety AND behaviors OR All Fields:safety AND behaviours OR All Fields:safety AND policy OR All Fields:safety AND procedure, Year: from 2024 to 2024 | 4 | 2024-12-13 13:55:31 |
|  | [S7](https://chiroindex.org/?action=set&setId=11515607) | Subject:\"Culture\" OR Subject:\"Organizational Culture\" OR Subject:\"Attitude\" OR Subject:\"Attitude of Health Personnel\" OR Subject:\"Health Knowledge, Attitudes, Practice\" OR Subject:\"Patient Care Team\", Year: from 2024 to 2024 OR All Fields:teamwork OR All Fields:rounds OR All Fields:learning AND organization OR All Fields:learning AND culture OR All Fields:safety AND culture OR All Fields:safety AND climate OR All Fields:safety AND environment OR All Fields:safety AND beliefs OR All Fields:safety AND performance OR All Fields:safety AND practices OR All Fields:safety AND behaviors OR All Fields:safety AND behaviours OR All Fields:safety AND policy OR All Fields:safety AND procedure, Year: from 2024 to 2024 | 6 | 2024-12-13 15:47:07 |
|  | [S8](https://chiroindex.org/?action=set&setId=11515612) | Subject:\"Patients\" OR Subject:\"Patient Care\" OR All Fields:patient OR All Fields:patients OR All Fields:client OR All Fields:clients OR All Fields:inpatient OR All Fields:inpatients OR All Fields:outpatient OR All Fields:outpatients, Year: from 2024 to 2024 AND Subject:\"Safety\" OR Subject:\"Safety Management\" OR Subject:\"Patient Safety\" OR Subject:\"Accidents\" OR Subject:\"Accidental Falls\" OR Subject:\"Accident Prevention\" OR Subject:\"Medical Errors\" OR Subject:\"Risk\" OR Subject:\"Chiropractic / adverse effects\", Year: from 2024 to 2024 OR All Fields:safe OR All Fields:safety OR All Fields:accident OR All Fields:accidents OR All Fields:accidental OR All Fields:fall OR All Fields:falls OR All Fields:mistake OR All Fields:mistakes OR All Fields:mistaken OR All Fields:adverse OR All Fields:harm OR All Fields:harms OR All Fields:harmful OR All Fields:error OR All Fields:errors, Year: from 2024 to 2024 AND Subject:\"Culture\" OR Subject:\"Organizational Culture\" OR Subject:\"Attitude\" OR Subject:\"Attitude of Health Personnel\" OR Subject:\"Health Knowledge, Attitudes, Practice\" OR Subject:\"Patient Care Team\", Year: from 2024 to 2024 OR All Fields:teamwork OR All Fields:rounds OR All Fields:learning AND organization OR All Fields:learning AND culture OR All Fields:safety AND culture OR All Fields:safety AND climate OR All Fields:safety AND environment OR All Fields:safety AND beliefs OR All Fields:safety AND performance OR All Fields:safety AND practices OR All Fields:safety AND behaviors OR All Fields:safety AND behaviours OR All Fields:safety AND policy OR All Fields:safety AND procedure, Year: from 2024 to 2024 | 4 | 2024-12-13 15:48:20 |

**Google Scholar**

Original Search: Friday, April 12, 2024

chiropractic|manipulation|adjustment patient|client|inpatient|outpatient safety|accident|fall|harm|error|adverse culture|climate|organization|beliefs|practices|attitude|teamwork|rounds|policies|environment

About 3,670,000 results

990 downloaded

Search Update: Mon, December 16, 2024

About 4,000,000 results

100 downloaded
