# Supplementary material for: Patient safety culture research within the chiropractic profession: a scoping review
Source: Chiropr Man Therap. 2025 Oct 21;33:46. doi: 10.1186/s12998-025-00605-z (PMC12538883; doi:10.1186/s12998-025-00605-z)
Supplement: Supplementary file 2 — Supplementary Material 2 [file 12998_2025_605_MOESM2_ESM.docx]

**Additional File 2 – Reasons for Article Exclusion During Full Text Screening**

***Best Practice Documents (14 full-text articles)***

1. Blanchette MA, Mior S, Thistle S, Stuber K. Developing key performance indicators for the Canadian chiropractic profession: a modified Delphi study. Chiropr Man Therap. 2022 Dec;30(1):31.

2. Brouwers M, Charette M. Evaluation of clinical practice guidelines in chiropractic care: a comparison of North American guideline reports. J Can Chiropr Assoc. 2001 Sep;45(3):141–53.

3. Bryans R, Descarreaux M, Duranleau M, Marcoux H, Potter B, Ruegg R, et al. Evidence-based guidelines for the chiropractic treatment of adults with headache. J Manipulative Physiol Ther. 2011 Jun;34(5):274–89.

4. Globe G, Farabaugh RJ, Hawk C, Morris CE, Baker G, Whalen WM, et al. Clinical practice guideline: chiropractic care for low back pain. J Manipulative Physiol Ther. 2016 Jan;39(1):1–22.

5. Hawk C, Amorin-Woods L, Evans MW, Whedon JM, Daniels CJ, Williams RD, et al. The role of chiropractic care in providing health promotion and clinical preventive services for adult patients with musculoskeletal pain: a clinical practice guideline. J Altern Complement Med. 2021 Oct 1;27(10):850–67.

6. Hawk C, Schneider M, Dougherty P, Gleberzon BJ, Killinger LZ. Best practices recommendations for chiropractic care for older adults: results of a consensus process. J Manipulative Physiol Ther. 2010 Jul;33(6):464–73.

7. Hawk C, Schneider M, Ferrance RJ, Hewitt E, Van Loon M, Tanis L. Best practices recommendations for chiropractic care for infants, children, and adolescents: results of a consensus process. J Manipulative Physiol Ther. 2009 Oct;32(8):639–47.

8. Hawk C, Schneider MJ, Haas M, Katz P, Dougherty P, Gleberzon B, et al. Best practices for chiropractic care for older adults: a systematic review and consensus update. J Manipulative Physiol Ther. 2017 May;40(4):217–29.

9. Hawk C, Schneider MJ, Vallone S, Hewitt EG. Best practices for chiropractic care of children: a consensus update. J Manipulative Physiol Ther. 2016 Mar;39(3):158–68.

10. Keating G, Hawk C, Amorin-Woods L, Amorin-Woods D, Vallone S, Farabaugh R, et al. Clinical practice guideline for best practice management of pediatric patients by chiropractors: results of a Delphi consensus process. J Integr Complement Med. 2024 Mar 1;30(3):216–32.

11. Lisi AJ, Salsbury SA, Hawk C, Vining RD, Wallace RB, Branson R, et al. Chiropractic integrated care pathway for low back pain in veterans: results of a Delphi consensus process. J Manipulative Physiol Ther. 2018 Feb;41(2):137–48.

12. Weis CA, Pohlman K, Barrett J, Clinton S, Da Silva-Oolup S, Draper C, et al. Best-practice recommendations for chiropractic care for pregnant and postpartum patients: results of a consensus process. J Manipulative Physiol Ther. 2022 Sep;45(7):469–89.

13. Whalen W, Farabaugh RJ, Hawk C, Minkalis AL, Lauretti W, Crivelli LS, et al. Best-practice recommendations for chiropractic management of patients with neck pain. J Manipulative Physiol Ther. 2019 Nov;42(9):635–50.

14. Whalen WM, Hawk C, Farabaugh RJ, Daniels CJ, Taylor DN, Anderson KR, et al. Best practices for chiropractic management of adult patients with mechanical low back pain: a clinical practice guideline for chiropractors in the United States. J Manipulative Physiol Ther. 2022 Oct;45(8):551–65.

***Case Studies of a Single Individual (42 studies)***

1. Brynin R, Yomtob C. Missed cervical spine fracture: chiropractic implications. J Manipulative Physiol Ther. 1999 Nov;22(9):610–4.

2. Chen Q, Feng J fei, Tang X, Li Y ling, Chen L, Chen G. Cervical epidural hematoma after spinal manipulation therapy: a case report. BMC Musculoskelet Disord. 2019 Dec;20(1):461.

3. Davis MA, Taylor JAM. A case of vertebral metastasis with pathologic C2 fracture. J Manipulative Physiol Ther. 2007 Jul;30(6):466–71.

4. de Boer NJ, Knaap SFC, de Zoete A. Clinical detection of abdominal aortic aneurysm in a 74-year-old man in chiropractic practice. J Chiropr Med. 2010 Mar;9(1):38–41.

5. Ea HK, Weber AJ, Yon F, Lioté F. Osteoporotic fracture of the dens revealed by cervical manipulation. Joint Bone Spine. 2004 May;71(3):246–50.

6. Fallon J. Chiropractic manipulation in the treatment of costovertebral joint dysfunction with resultant intercostal neuralgia during pregnancy. J Neuromusculoskelet Syst. 1996;4:73–5.

7. Gleberzon B, Hyde D. Hip fracture presenting as mechanical low back pain subsequent to a fall: a case study. J Can Chiropr Assoc. 2006 Dec;50(4):255–62.

8. Guebert G, Ryan T, Rehberger J. Missed diagnosis, manipulative nightmare: a case report and literature review. J Neuromusculoskelet Syst. 1995;3:92–5.

9. Hadida C, Lemire J. Missed upper cervical spine fracture: clinical and radiological considerations. J Can Chiropr Assoc. 1997 Jun;41(2):77–85.

10. Horn SW. The “locked-in” syndrome following chiropractic manipulation of the cervical spine. Ann Emerg Med. 1983 Oct;12(10):648–50.

11. Horsburgh J. Case report of a deep vein thrombosis in the femoral vein, an atypical presentation. Clin Chiropr. 2004 Sep;7(3):120–6.

12. Jacobs CL, Stern PJ. An unusual case of gout in the wrist: the importance of monitoring medication dosage and interaction. A case report. Chiropr Man Therap. 2007 Dec;15(1):16.

13. Johnson TL. Abdominal and back pain in a 65-year-old patient with metastatic prostate cancer. J Chiropr Med. 2010 Mar;9(1):11–6.

14. Kaur RA. Co-morbidity of low back pain and abdominal aortic aneurysm: a case report. Clin Chiropr. 2004 Jun;7(2):67–72.

15. Kavanagh KJ, Parrilla D. The role of a chiropractor upon presentation of dermatomyositis to a chiropractic clinic: a case report. J Int Acad Neuromusculoskel Med. 2024;21(2):11–8.

16. Kurbanyan K, Lessell S. Intracranial hypotension and abducens palsy following upper spinal manipulation. Br J Ophthalmol. 2008 Jan 1;92(1):153–5.

17. Liao CC, Chen LR. Anterior and posterior fixation of a cervical fracture induced by chiropractic spinal manipulation in ankylosing spondylitis: a case report. J Trauma. 2007 Oct;63(4):E90–4.

18. Lishchyna N, Henderson S. Acute onset-low back pain and hip pain secondary to metastatic prostate cancer: a case report. J Can Chiropr Assoc. 2004 Mar;48(1):5–12.

19. Morton A. Internal carotid artery dissection following chiropractic treatment in a pregnant woman with Systemic Lupus Erythematosus. Chiropr Man Therap. 2012 Dec;20(1):38.

20. Muligano D, Serlin A, Sidden T, Cramer GD. Self-administered traction as an adjunct in the chiropractic treatment of low back pain: a case report. J Chiropr Med. 2024 Dec;23(4):205–14.

21. Munro D. Missed C1 posterior arch fracture: a case report. J Can Chiropr Assoc. 1990 Mar;34(1):27–9.

22. Murphy DR, Beres JL. Cervical myelopathy: a case report of a “near-miss” complication to cervical manipulation. J Manipulative Physiol Ther. 2008 Sep;31(7):553–7.

23. Nadgir RN, Loevner LA, Ahmed T, Moonis G, Chalela J, Slawek K, et al. Simultaneous bilateral internal carotid and vertebral artery dissection following chiropractic manipulation: case report and review of the literature. Neuroradiology. 2003 May;45(5):311–4.

24. Ng K, Doube A. Stroke after neck manipulation in the post partum period. N Z Med J. 2001 Nov;114(1143):498.

25. Oakley PA, Gage WH, Harrison DE, Mochizuki G. Non-surgical reduction in thoracolumbar kyphosis and sagittal vertical axis corresponding with improved sensorimotor control in an older adult with spinal deformity: a Chiropractic Biophysics case report. J Phys Ther Sci. 2024;36(11):756–64.

26. Patel SN, Kettner NW. Abdominal aortic aneurysm presenting as back pain to a chiropractic clinic: a case report. J Manipulative Physiol Ther. 2006 Jun;29(5):409.e1-409.e7.

27. Plezbert JA, Oestreich AT. Fracture of a lamina in the cervical spine. J Manipulative Physiol Ther. 1994 Oct;17(8):552–7.

28. Price M, Ravanpay A, Daniels C. Recognition of prodromal cervical spondylotic myelopathy presenting in a U.S. Veteran referred to chiropractic for acute thoracic pain: a case report. J Bodyw Mov Ther. 2021 Oct;28:13–7.

29. Rectenwald R. A case study of back pain and renal cell carcinoma. J Chiropr Med. 2008 Mar;7(1):24–7.

30. Sayed AG, Aboloyoun H, Makarem YS, Elnaggar A. Internal carotid artery pseudoaneurysm after neck manipulation in a patient with Eagle syndrome. Clin Case Rep. 2024 May;12(5):e8814.

31. Schram DJ, Vosik W, Cantral D. Diaphragmatic paralysis following cervical chiropractic manipulation. Chest. 2001 Feb;119(2):638–9.

32. Shafrir Y, Kaufman BA. Quadriplegia after chiropractic manipulation in an infant with congenital torticollis caused by a spinal cord astrocytoma. J Pediatr. 1992 Feb;120(2):266–9.

33. Sherman P, Lehman J. Abdominal aortic aneurysm (AAA): evaluation and management by a doctor of chiropractic. JACA Online. 2008 May;45(4):12–8.

34. Troyanovich S. C1 burst fracture. J Manipulative Physiol Ther. 1994 Oct;17(8):558–61.

35. Tseng S, Chen Y, Lin S, Wang C. Cervical epidural hematoma after spinal manipulation therapy: case report. J Trauma. 2002 Mar;52(3):585–6.

36. Turan B, Sonel TB. Rehabilitation in a case with cauda equina syndrome after spinal manipulation: a long and difficult process. Turk J Phys Med Rehab. 2025;71(x):i–v.

37. Ursprung WM, Kettner NW, Boesch R. Vertebral osteomyelitis: a case report of a patient presenting with acute low back pain. J Manipulative Physiol Ther. 2005 Nov;28(9):713–8.

38. Wang CC, Kuo JR, Chio CC, Tsai TC. Acute paraplegia following chiropractic therapy. J Clin Neurosci. 2006 Jun;13(5):578–81.

39. Whedon JM, Quebada PB, Roberts DW, Radwan TA. Spinal epidural hematoma after spinal manipulative therapy in a patient undergoing anticoagulant therapy: a case report. J Manipulative Physiol Ther. 2006 Sep;29(7):582–5.

40. Winter A. Atypical polymyalgia rheumatica in a 70-year-old male chiropractic patient: a case report. Clin Chiropr. 2004 Dec;7(4):174–9.

41. Woodham TJ, Fortner MO, Haas JW, Oakley PA, Harrison DE. The reduction of cervical hyperlordosis and resolution of craniocervical symptoms in an adolescent female: a Chiropractic Biophysics case report with long-term follow-up. Cureus. 2024 Sep;16(9):e69913.

42. Woodhouse D. Post-traumatic compression fracture. Clin Chiropr. 2003 Feb;6:67–72.

***No Patient Safety Culture Focus (98 studies)***

1. Alcantara J, Ohm J, Kunz D. The safety and effectiveness of pediatric chiropractic: a survey of chiropractors and parents in a practice-based research network. EXPLORE. 2009 Sep;5(5):290–5.

2. Alcantara J, Whetten A, Ohm J, Alcantara J. Ratings of perceived effectiveness, patient satisfaction and adverse events experienced by wellness chiropractic patients in a practice-based research network. Complement Ther Clin Pract. 2019 Aug;36:82–7.

3. Beattie PF, Nelson R, Murphy DR. Development and preliminary validation of the MedRisk Instrument to measure patient satisfaction with chiropractic care. J Manipulative Physiol Ther. 2011 Jan;34(1):23–9.

4. Bussières AE, Al Zoubi F, Stuber K, French SD, Boruff J, Corrigan J, et al. Evidence-based practice, research utilization, and knowledge translation in chiropractic: a scoping review. BMC Complement Altern Med. 2016 Dec;16(1):216.

5. Cagnie B, Vinck E, Beernaert A, Cambier D. How common are side effects of spinal manipulation and can these side effects be predicted? Man Ther. 2004 Aug;9(3):151–6.

6. Carnes D, Mars TS, Mullinger B, Froud R, Underwood M. Adverse events and manual therapy: A systematic review. Man Ther. 2010 Aug;15(4):355–63.

7. Cassidy JD, Boyle E, Côté P, He Y, Hogg-Johnson S, Silver FL, et al. Risk of vertebrobasilar stroke and chiropractic care: results of a population-based case-control and case-crossover Study. Spine. 2008 Feb;33(Supplement):S176–83.

8. Cassidy JD, Boyle E, Côté P, Hogg-Johnson S, Bondy SJ, Haldeman S. Risk of carotid stroke after chiropractic care: a population-based case-crossover study. J Stroke Cerebrovasc Dis. 2017 Apr;26(4):842–50.

9. Chu ECP, Trager RJ, Lee LYK, Niazi IK. A retrospective analysis of the incidence of severe adverse events among recipients of chiropractic spinal manipulative therapy. Sci Rep. 2023 Jan 23;13(1):1254.

10. Chung CLR, Côté P, Stern P, L’Espérance G. The association between cervical spine manipulation and carotid artery dissection: a systematic review of the literature. J Manipulative Physiol Ther. 2015 Nov;38(9):672–6.

11. Corso M, Cancelliere C, Mior S, Taylor-Vaisey A, Côté P. The safety of spinal manipulative therapy in children under 10 years: a rapid review. Chiropr Man Therap. 2020 Dec;28(1):12.

12. Coulter ID, Singh BB, Riley D, Der-Martirosian C. Interprofessional referral patterns in an integrated medical system. J Manipulative Physiol Ther. 2005 Mar;28(3):170–4.

13. Daniel DM, Ndetan H, Rupert RL, Martinez D. Self-reported recognition of undiagnosed life threatening conditions in chiropractic practice: a random survey. Chiropr Man Therap. 2012 Jul 5;20:21.

14. Debarle M, Aigron R, Depernet L, Guillemard A, Véron T, Leboeuf-Yde C. Management of patients with low back pain: a survey of French chiropractors. Chiropr Man Therap. 2014 Mar 28;22(1):13.

15. Domenicucci M, Ramieri A, Salvati M, Brogna C, Raco A. Cervicothoracic epidural hematoma after chiropractic spinal manipulation therapy: case report and review of the literature. J Neurosurg Spine. 2007 Nov;7(5):571–4.

16. Dunn AS, Green BN, Gilford S. An analysis of the integration of chiropractic services within the United States Military and veterans’ health care systems. J Manipulative Physiol Ther. 2009 Nov;32(9):749–57.

17. Dunn AS, Towle JJ, McBrearty P, Fleeson SM. Chiropractic consultation requests in the Veterans Affairs health care system: demographic characteristics of the initial 100 patients at the Western New York Medical Center. J Manipulative Physiol Ther. 2006 Jul;29(6):448–54.

18. Eriksen K, Rochester RP, Hurwitz EL. Symptomatic reactions, clinical outcomes and patient satisfaction associated with upper cervical chiropractic care: A prospective, multicenter, cohort study. BMC Musculoskelet Disord. 2011 Dec;12(1):219.

19. Ernst E. Prospective investigations into the safety of spinal manipulation. J Pain Symptom Manage. 2001 Mar;21(3):238–42.

20. Gouveia LO, Castanho P, Ferreira JJ. Safety of chiropractic interventions: a systematic review. Spine. 2009 May;34(11):E405–13.

21. Greene BR, Smith M, Haas M, Allareddy V. How often are physicians and chiropractors provided with patient information when accepting referrals? J Ambul Care Manage. 2007 Oct;30(4):344–6.

22. Guenoun O, Debarle M, Garnesson C, Proisl S, Ray D, Leboeuf-Yde C. Case management of chiropractic patients with cervical brachialgia: A survey of French chiropractors. Chiropr Man Therap. 2011 Dec;19(1):23.

23. Haldeman S, Kohlbeck FJ, McGregor M. Risk factors and precipitating neck movements causing vertebrobasilar artery dissection after cervical trauma and spinal manipulation: Spine. 1999 Apr;24(8):785–94.

24. Haldeman S, Kohlbeck FJ, McGregor M. Unpredictability of cerebrovascular ischemia associated with cervical spine manipulation therapy: a review of sixty-four cases after cervical spine manipulation. Spine. 2002 Jan;27(1):49–55.

25. Haldeman S, Kohlbeck FJ, McGregor M. Stroke, cerebral artery dissection, and cervical spine manipulation therapy. J Neurol. 2002 Jul 1;249(8):1098–104.

26. Haldeman S, Rubinstein SM. Cauda equina syndrome in patients undergoing manipulation of the lumbar spine: Spine. 1992 Dec;17(12):1469–73.

27. Hawk C, Dusio ME. A survey of 492 U.S. chiropractors on primary care and prevention-related issues. J Manipulative Physiol Ther. 1995 Feb;18(2):57–64.

28. Haworth NG, Horstmanshof L, Moore KM. Chiropractic and osteopathic students’ perceptions of readiness for transition to practice: *the educational value of university clinic vs community and private clinics*. J Chiropr Educ. 2021 Mar 1;35(1):38–49.

29. Heneghan NR, Pup C, Koulidis K, Rushton A. Thoracic adverse events following spinal manipulative therapy: a systematic review and narrative synthesis. J Man Manip Ther. 2020 Oct 19;28(5):275–86.

30. Hincapié CA, Cassidy JD, Côté P, Rampersaud YR, Jadad AR, Tomlinson GA. Chiropractic spinal manipulation and the risk for acute lumbar disc herniation: a belief elicitation study. Eur Spine J. 2018 Jul;27(7):1517–25.

31. Humphreys BK. Possible adverse events in children treated by manual therapy: a review. Chiropr Man Therap. 2010 Dec;18(1):12.

32. Hurwitz EL, Morgenstern H, Vassilaki M, Chiang LM. Adverse reactions to chiropractic treatment and their effects on satisfaction and clinical outcomes among patients enrolled in the UCLA Neck Pain Study. J Manipulative Physiol Ther. 2004 Jan;27(1):16–25.

33. Hurwitz EL, Morgenstern H, Vassilaki M, Chiang LM. Frequency and clinical predictors of adverse reactions to chiropractic care in the UCLA Neck Pain Study: Spine. 2005 Jul;30(13):1477–84.

34. Innes SI, Cope V, Leboeuf-Yde C, Walker BF. A perspective on Chiropractic Councils on Education accreditation standards and processes from the inside: a narrative description of expert opinion, part 1: Themes. Chiropr Man Therap. 2019 Dec;27(1):57.

35. Innes SI, Cope V, Leboeuf-Yde C, Walker BF. A perspective on Councils on Chiropractic Education accreditation standards and processes from the inside: a narrative description of expert opinion, part 2: Analyses of particular responses to research findings. Chiropr Man Therap. 2019 Dec;27(1):56.

36. Innes SI, Kimpton A. Are Councils on Chiropractic Education expectations of chiropractic graduates changing for the better: a comparison of similarities and differences of the graduate competencies of the Chiropractic Council on Education-Australasia from 2009 to 2017. Chiropr Man Therap. 2020 Dec;28(1):30.

37. Innes SI, Leboeuf-Yde C, Walker BF. Similarities and differences of a selection of key accreditation standards between chiropractic councils on education: a systematic review. Chiropr Man Therap. 2016 Dec;24(1):46.

38. Innes SI, Leboeuf-Yde C, Walker BF. The relationship between intolerance of uncertainty in chiropractic students and their treatment intervention choices. Chiropr Man Therap. 2017;25:20.

39. Innes SI, Leboeuf-Yde C, Walker BF. Comparing the old to the new: a comparison of similarities and differences of the accreditation standards of the chiropractic council on education-international from 2010 to 2016. Chiropr Man Therap. 2018 Dec;26(1):25.

40. Innes SI, Leboeuf-Yde C, Walker BF. Attempting to explore chiropractors and their clinical choices: an examination of a failed study. Chiropr Man Therap. 2019 Dec;27(1):15.

41. Jenkins H, Zheng X, Bull P. Prevalence of congenital anomalies contraindicating spinal manipulative therapy within a chiropractic patient population. Chiropr J Aust. 2010;40(2):69–76.

42. Johnson CD, Green BN, Konarski-Hart KK, Hewitt EG, Napuli JG, Foshee WK, et al. Response of practicing chiropractors during the early phase of the COVID-19 pandemic: a descriptive report. J Manipulative Physiol Ther. 2020 Jun;43(5):403.e1-403.e21.

43. Keter DL, Bent JA, Bialosky JE, Courtney CA, Esteves JE, Funabashi M, et al. An international consensus on gaps in mechanisms of forced-based manipulation research: findings from a nominal group technique. J Man Manip Ther. 2024 Jan 2;32(1):111–7.

44. King SW, Hosler BK, King MA, Eiselt EW. Missed cervical spine fracture-dislocations: the importance of clinical and radiographic assessment. J Manipulative Physiol Ther. 2002 May;25(4):263–9.

45. Kitchiner R. The role of the personal digital assistant (PDA) in chiropractic practice. Clin Chiropr. 2006 Sep;9(3):119–28.

46. Klougart N, Leboeuf-Yde C, Rasmussen LR. Safety in chiropractic practice, Part I; The occurrence of cerebrovascular accidents after manipulation to the neck in Denmark from 1978-1988. J Manipulative Physiol Ther. 1996;19(6):371–7.

47. Klougart N, Leboeuf-Yde C, Rasmussen LR. Safety in chiropractic practice. Part II: Treatment to the upper neck and the rate of cerebrovascular incidents. J Manipulative Physiol Ther. 1996;19(9):563–9.

48. Lawrence D. Designing core clinical bioethics training for Master’s level students in America. Chiropr J Aust. 2008;38(2):57–68.

49. Leboeuf-Yde C, Hennius B, Rudberg E, Leufvenmark P, Thunman M. Side effects of chiropractic treatment: a prospective study. J Manipulative Physiol Ther. 1997 Oct;20(8):511–5.

50. Maiers M, Evans R, Hartvigsen J, Schulz C, Bronfort G. Adverse events among seniors receiving spinal manipulation and exercise in a randomized clinical trial. Man Ther. 2015 Apr;20(2):335–41.

51. Maiers MJ, Foshee WK, Henson Dunlap H. Culturally sensitive chiropractic care of the transgender community: a narrative review of the literature. J Chiropr Humanit. 2017 Dec;24(1):24–30.

52. Mainous Iii AG. Fragmentation of patient care between chiropractors and family physicians. Arch Fam Med. 2000 May 1;9(5):446–50.

53. Malone DG, Baldwin NG, Tomecek FJ, Boxell CM, Gaede SE, Covington CG, et al. Complications of cervical spine manipulation therapy: 5-year retrospective study in a single-group practice. Neurosurg Focus. 2002 Dec;13(6):1–8.

54. Marcon AR, Klostermann P, Caulfield T. Chiropractic and spinal manipulation therapy on Twitter: case study examining the presence of critiques and debates. JMIR Public Health Surveill. 2016 Sep 16;2(2):e153.

55. Milne N, Longeri L, Patel A, Pool J, Olson K, Basson A, et al. Spinal manipulation and mobilisation in the treatment of infants, children, and adolescents: a systematic scoping review. BMC Pediatr. 2022 Dec 19;22(1):721.

56. Miraglia CM, March Mistler J, Baird GL. Hand hygiene practices, attitudes, and microbial hand populations of practicing Doctors of Chiropractic.  J Chiropr Med. 2020 Mar;19(1):9–20.

57. Murphy DR. Herniated disc with radiculopathy following cervical manipulation: nonsurgical management. Spine J. 2006 Jul;6(4):459–63.

58. Murphy DR, Hurwitz EL, Gregory AA. Manipulation in the presence of cervical spinal cord compression: a case series. J Manipulative Physiol Ther. 2006 Mar;29(3):236–44.

59. Myers CA, Enebo BA, Davidson BS. Optimized prediction of contact force application during side-lying lumbar manipulation. J Manipulative Physiol Ther. 2012 Nov;35(9):669–77.

60. Nielsen SM, Tarp S, Christensen R, Bliddal H, Klokker L, Henriksen M. The risk associated with spinal manipulation: an overview of reviews. Syst Rev. 2017 Dec;6(1):64.

61. Odierna DH, Smith M. Education and patient care in a chiropractic teaching clinic: An organizational approach to health and safety during the COVID-19 pandemic. J Chiropr Educ. 2022 Oct 1;36(2):103–9.

62. Oliphant D. Safety of spinal manipulation in the treatment of lumbar disk herniations: a systematic review and risk assessment. J Manipulative Physiol Ther. 2004 Mar;27(3):197–210.

63. Oppenheim JS, Spitzer DE, Segal DH. Nonvascular complications following spinal manipulation. Spine J. 2005 Nov;5(6):660–6.

64. Orlandi G, Parenti G, Murri L, Bianchi M, Renna M, Martini A. Vertebral and carotid artery dissection following chiropractic cervical manipulation. Neurosurg Rev. 1999 Oct 8;22(2–3):127–9.

65. Paige NM, Miake-Lye IM, Booth MS, Beroes JM, Mardian AS, Dougherty P, et al. Association of spinal manipulative therapy with clinical benefit and harm for acute low back pain: systematic review and meta-analysis. JAMA. 2017 Apr 11;317(14):1451.

66. Patel A, Lee R, Fritz W, Matos E, Freeman JW. Vertebral artery dissection from cervical spine manipulation: case reports and analysis. S D Med. 2008 Mar;61(3):95, 97–9.

67. Penney LS, Ritenbaugh C, Elder C, Schneider J, Deyo RA, DeBar LL. Primary care physicians, acupuncture and chiropractic clinicians, and chronic pain patients: a qualitative analysis of communication and care coordination patterns. BMC Complement Altern Med. 2015 Dec;16(1):30.

68. Pizzi L, Goldfarb N, Nash D. Promoting a culture of safety. In: Making Health Care Safer: A Critical Analysis of Patient Safety Practices. Rockville (MD): Agency for Healthcare Research and Quality (US); 2013. Report No.: 2nd ed.

69. Plener J, Assimakopoulos D, Chung C, Hains F, Mior S. Exploring strategies to improve clinical decision making in a chiropractic office: a case series. J Can Chiropr Assoc. 2024;68(2):113–21.

70. Powell FC, Hanigan WC, Olivero WC. A risk/benefit analysis of spinal manipulation therapy for relief of lumbar or cervical pain. Neurosurgery. 1993 Jul;33(1):73–9.

71. Rubinstein SM, De Zoete A, Van Middelkoop M, Assendelft WJJ, De Boer MR, Van Tulder MW. Benefits and harms of spinal manipulative therapy for the treatment of chronic low back pain: systematic review and meta-analysis of randomised controlled trials. BMJ. 2019 Mar 13;1689.

72. Rubinstein SM, Knol DL, Leboeuf-Yde C, Van Tulder MW. Benign adverse events following chiropractic care for neck pain are associated with worse short-term outcomes but not worse outcomes at three months: Spine. 2008 Dec;33(25):E950–6.

73. Ryan AT, Too LS, Bismark MM. Complaints about chiropractors, osteopaths, and physiotherapists: a retrospective cohort study of health, performance, and conduct concerns. Chiropr Man Therap. 2018 Dec;26(1):12.

74. Salsbury SA, Goertz CM, Twist EJ, Lisi AJ. Integration of Doctors of Chiropractic into private sector health care facilities in the United States: a descriptive survey. J Manipulative Physiol Ther. 2018 Feb;41(2):149–55.

75. Salsbury SA, Vining RD, Gosselin D, Goertz CM. Be good, communicate, and collaborate: a qualitative analysis of stakeholder perspectives on adding a chiropractor to the multidisciplinary rehabilitation team. Chiropr Man Therap. 2018 Dec;26(1):29.

76. Senstad O, Leboeuf-Yde C, Borchgrevink C. Predictors of side effects to spinal manipulative therapy. J Manipulative Physiol Ther. 1996 Sep;19(7):441–5.

77. Senstad O, Leboeuf-Yde C, Borchgrevink C. Frequency and characteristics of side effects of spinal manipulative therapy: Spine. 1997 Feb;22(4):435–40.

78. Senstad O, Leboeuf-Yde C, Borchgrevink CF. Side-effects of chiropractic spinal manipulation: types, frequency, discomfort and course. Scand J Prim Health Care. 1996 Jan;14(1):50–3.

79. Shekelle PG, Hurwitz EL, Coulter I, Adams AH, Genovese B, Brook RH. The appropriateness of chiropractic spinal manipulation for low back pain: a pilot study. J Manipulative Physiol Ther. 1995 Jun;18(5):265–70.

80. Skappak C, Saude EJ. Back pain in the emergency department: pathological fracture following spinal manipulation. CJEM. 2018 Mar;20(2):307–12.

81. Smith M, Carber LA. Chiropractors as safety net providers: first report of findings and methods from a US survey of chiropractors. J Manipulative Physiol Ther. 2007 Nov;30(9):718–28.

82. Smith RA. Neurologic complications of head and neck manipulations: report of two cases. JAMA. 1962 Nov 3;182(5):528.

83. Song Y ping, Liu J li, Zong C zhong, Zhang F shuo, Ren Y feng, Ching YL, et al. A bibliometric study on trends in chiropractic research from 1920 to 2023. Complement Ther Med. 2024 Jun;82:103038.

84. Strunk RG, Hawk C. Effects of chiropractic care on dizziness, neck pain, and balance: a single-group, preexperimental, feasibility study. J Chiropr Med. 2009 Dec;8(4):156–64.

85. Stuber K. The safety of chiropractic during pregnancy: a pilot e-mail survey of chiropractors’ opinions. Clin Chiropr. 2007 Mar;10(1):24–35.

86. Stuber KJ, Wynd S, Weis CA. Adverse events from spinal manipulation in the pregnant and postpartum periods: a critical review of the literature. Chiropr Man Therap. 2012 Dec;20(1):8.

87. Terrett A. Vascular accidents from cervical spine manipulation: report on 107 cases. J Aust Chiropr Assoc. 1987 Mar;17(1):15–24.

88. Thiel HW, Bolton JE, Docherty S, Portlock JC. Safety of chiropractic manipulation of the cervical spine: a prospective national survey. Spine. 2007 Oct;32(21):2375–8.

89. Toth E, Lawson D, Nykoliation J. Chiropractic complaints and disciplinary cases in Canada. J Can Chiropr Assoc. 1998;42(4):229–42.

90. Trager RJ, Burton WM, Loewenthal JV, Perez JA, Lisi AJ, Kowalski MH, et al. Chiropractic spinal manipulation and fall risk in older adults with spinal pain: Observational findings from a matched retrospective cohort study. Cureus. 2024 Oct;16(10):e72330

91. Triano JJ, Goertz C, Weeks J, Murphy DR, Kranz KC, McClelland GC, et al. Chiropractic in North America: toward a strategic plan for professional renewal—outcomes from the 2006 Chiropractic Strategic Planning Conference. J Manipulative Physiol Ther. 2010 Jun;33(5):395–405.

92. Vohra S, Kawchuk GN, Boon H, Caulfield T, Pohlman KA, O’Beirne M. SafetyNET: An interdisciplinary research program to support a safety culture for spinal manipulation therapy. Eur J Integr Med. 2014 Aug;6(4):473–7.

93. Whedon JM, Toler AWJ, Goehl JM, Kazal LA. Association between utilization of chiropractic services for treatment of low back pain and risk of adverse drug events. J Manipulative Physiol Ther. 2018 Jun;41(5):383–8.

94. Wiesener S, Salamonsen A, Fønnebø V. Which risk understandings can be derived from the current disharmonized regulation of complementary and alternative medicine in Europe? BMC Complement Altern Med. 2018 Dec;18(1):11.

95. Wiggins D, Downie A, Engel R, Brown BT. Factors that influence scope of practice of the chiropractic profession in Australia: a scoping review. Chiropr Man Therap. 2022 Dec;30(1):19.

96. Woggon AJ, Woggon DA. Patient-reported side effects immediately after chiropractic scoliosis treatment: a cross-sectional survey utilizing a practice-based research network. Scoliosis. 2015 Dec;10(1):29.

97. Young KJ, Young HC, Field J. A qualitative analysis of free-text patient satisfaction responses in Care Response, a database of patient-reported outcome and experience measures. Chiropr Man Therap. 2024 Jan 29;32(1):2.

98. Zaugg B, Wangler M. A model framework for patient safety training in chiropractic: a literature synthesis. J Manipulative Physiol Ther. 2009 Jul;32(6):493–9.

***Not Available In English (2 studies)***

1. Krieger D, Leibold M, Brückmann H. Dissektionen der arteria vertebralis nach zervikalen chiropraktischen manipulationen. Dtsch med Wochenschr. 2008 Mar 25;115(15):580–3.

2. Oehler J, Gandjour J, Fiebach J, Schwab S. Beidseitge A. Vertebralis dissektion nach chiropraktischer behandlungn konservativer behandlungsmethoden bei patienten mit claudicatio spinalis. Der Orthopade. 2003 Oct 1;32(10):911–3.

***Not Chiropractic Related (10 studies)***

1. Bernetti A, La Russa R, De Sire A, Agostini F, De Simone S, Farì G, et al. Cervical spine manipulations: role of diagnostic procedures, effectiveness, and safety from a rehabilitation and forensic medicine perspective: a systematic review. Diagnostics. 2022 Apr 23;12(5):1056.

2. Carroll L, Cassidy JD, Peloso P, Borg J, Von Holst H, Holm L, et al. Prognosis for mild traumatic brain injury: results of the who collaborating centre task force on mild traumatic brain injury. J Rehabil Med. 2004 Feb 1;36(0):84–105.

3. Caspi O, Holexa J. Lack of standards in informed consent in complementary and alternative medicine. Complement Ther Med. 2005 Jun;13(2):123–30.

4. Funabashi M, Carlesso LC. Symptoms patients receiving manual therapy experienced and perceived as adverse: a secondary analysis of a survey of patients’ perceptions of what constitutes an adverse response. J Manipulative Physiol Ther. 2021 Jan 2;29(1):51–8.

5. Gibbons P, Tehan P. Spinal manipulation: Indications, risks and benefits. J Bodyw Mov Ther. 2001 Apr;5(2):110–9.

6. Hufnagel A, Hammers A, Schanle PW, Bohm KD, Leonhardt G. Stroke following chiropractic manipulation of the cervical spine. J Neurol. 1999 Aug 23;246(8):683-8.

7. Kranenburg HA (Rik), Schmitt MA, Puentedura EJ, Van Der Schans CP, Heneghan NR, Hutting N. Manual therapists’ beliefs and use of spinal thrust joint manipulation. Eur J Physiother. 2022 Sep 3;24(5):262–9.

8. Lee WT, Chu EC, Cheng, WK. Chiropractor perceptions of non-registered practitioners utilizing spinal manipulative therapy in Hong Kong: A cross-sectional survey. Cureus. 2024 Oct;16(10):e70734

9. Paanalahti K, Holm LW, Nordin M, Asker M, Lyander J, Skillgate E. Adverse events after manual therapy among patients seeking care for neck and/or back pain: a randomized controlled trial. BMC Musculoskelet Disord. 2014 Dec;15(1):77.

10. Tabell V, Tarkka IM, Holm LW, Skillgate E. Do adverse events after manual therapy for back and/or neck pain have an impact on the chance to recover? A cohort study. Chiropr Man Therap. 2019 Dec;27(1):27.

***Not Original Research (36 studies)***

1. Alcantara J, Ohm J, Kunz D. Treatment‐related aggravations, complications and improvements attributed to chiropractic spinal manipulative therapy of paediatric patients: a practice‐based survey of practitioners. Focus Altern Complement Ther. 2007 Dec;12(s1):3–3.

2. Amorin-Woods LG, Parkin-Smith GF. Clinical decision-making to facilitate appropriate patient management in chiropractic practice: “the 3-questions model.” Chiropr Man Therap. 2012 Dec;20(1):6.

3. Anders J, March J, Perez A. Safety of cervical manipulation: are adverse events preventable and are manipulations being performed appropriately? 2011 [cited 2024 Oct 20]; Available from: <https://digitalscholarship.unlv.edu/thesesdissertations/1303>

4. Boggs S. Informed consent: the patient’s right to choose. J Chiropr. 1992;29:36–42.

5. Burgess M. Chiropractic informed consent. J Can Chiropr Assoc. 1990;34(1):24–6.

6. Bussières A, Côté P, French S, Godwin M, Gotlib A, Graham ID, et al. Creating a Chiropractic Practice-Based Research Network (PBRN): enhancing the management of musculoskeletal care. J Can Chiropr Assoc. 2014 Mar;58(1):8–15.

7. Dalgaard J. Medico-legal aspects of manipulatory treatment in Denmark. J Man Med. 1991;6:114–6.

8. DiGiorgi D, Cerf JL, Bowerman DS. Outcomes indicators and a risk classification system for spinal manipulation under anesthesia: a narrative review and proposal. Chiropr Man Therap. 2018 Dec;26(1):9.

9. Dougherty PE, Hawk C, Weiner DK, Gleberzon B, Andrew K, Killinger L. The role of chiropractic care in older adults. Chiropr Man Therap. 2012 Feb 21;20(1):3.

10. Doyle M. Chiropractic care for infants and children-there is no evidence for it and it is dangerous?: a selective review of the literature and commentary. Chiropr J Aust. 2016;44:222–33.

11. Finch R, Heale G, Jay T. Culture of safety among UK chiropractors before and after the launch of online patient safety incident reporting and learning. Clin Chiropr. 2010 Jun;13(2):172–3.

12. Fuller S. Evaluating chiropractic students hand washing practices using GloGermTM as a surrogate for microbial pathogens. University of Johannesburg; 2019.

13. Funabashi M, Pohlman KA, Gorrell LM, Salsbury SA, Bergna A, Heneghan NR. Expert consensus on a standardised definition and severity classification for adverse events associated with spinal and peripheral joint manipulation and mobilisation: protocol for an international e-Delphi study. BMJ Open. 2021 Nov;11(11):e050219.

14. Ganesh N. Clinical experiences of first-time registered Master’s chiropractic students during their clinical practicum [Internet] [Master’s Degree in Technology: Chiropractic]. [Durban, South Africa]: Durban University of Technology; 2017 [cited 2024 Oct 20]. Available from: <http://hdl.handle.net/10321/2896>

15. Innes SI, Leboeuf-Yde C, Walker BF. The accreditation role of Councils on Chiropractic Education as part of the profession’s journey from craft to allied health profession: a commentary. Chiropr Man Therap. 2020 Dec;28(1):40.

16. Johnson C, Green B. Chiropractic care. In: Complementary and integrative therapies for mental health and aging. New York: Oxford University Press; 2009. p. 125–38.

17. Johnson C, Baird R, Dougherty PE, Globe G, Green BN, Haneline M, et al. Chiropractic and public health: current state and future vision. J Manipulative Physiol Ther. 2008 Jul;31(6):397–410.

18. Johnson CD, Green BN. Looking back at the lawsuit that transformed the chiropractic profession: authors’ introduction. J Chiropr Educ. 2021 Sep 1;35(S1):5–8.

19. Johnson C, Rubinstein SM, Côté P, Hestbaek L, Injeyan HS, Puhl A, et al. Chiropractic care and public health: answering difficult questions about safety, care through the lifespan, and community action. J Manipulative Physiol Ther. 2012 Sep;35(7):493–513.

20. Kerry R, Young KJ, Evans DW, Lee E, Georgopoulos V, Meakins A, et al. A modern way to teach and practice manual therapy. Chiropr Man Therap. 2024 May 21;32(1):17.

21. Killinger L. Chiropractic student’s knowledge, attitudes, and interdisciplinary perceptions on caring for older patients. J Chiropr Educ. 2002;16(1):23.

22. Killinger L. Chiropractic adjusting and the “aging” patient. J Amer Chiropr Assoc. 2003;40(11):26–8.

23. Kinsinger S. A proposed bioethics curriculum for chiropractic colleges. Chiropr J Aust. 2016;44:290–302.

24. Leboeuf-Yde C, Hestbæk L. Chiropractic and children: is more research enough? Chiropr Osteopat. 2010 Jun 2;18:11.

25. Lehman JJ, Conwell TD, Sherman PR. Should the chiropractic profession embrace the doctrine of informed consent? J Chiropr Med. 2009 Jun;8(2):91–2.

26. Lyons L, Hang L, Ross MWV, Fredericks J, Fredericks M. Chiropractic Physicians: A functional conceptualization of electronic medical records technology and the society, culture, and personality model in the delivery of healthcare. J Chiropr Humanit. 2008 Jan;15:10–8.

27. Moore MP. Chiropractic patient safety education experiences: a phenomenological study. [Lynchburg, VA]: Liberty University; 2024.

28. Moreau W, Stoos W. Communication central to informed consent. J Amer Chiropr Assoc. 2006;43(3):15–8.

29. Pohlman KA, Carroll L, Tsuyuki RT, Hartling L, Vohra S. Active versus passive adverse event reporting after pediatric chiropractic manual therapy: study protocol for a cluster randomized controlled trial. Trials. 2017 Dec;18(1):575.

30. Pohlman K, O’Beirne M, Thiel H, Cassidy JD, Mior S, Hurwitz E, et al. Development and validation of instruments to evaluate adverse events after spinal manipulation therapy (abstract only). J Altern Complement Med. 2014 May;20(5):A49–A49.

31. Simpson JK, Innes S. Informed consent, duty of disclosure and chiropractic: where are we? Chiropr Man Therap. 2020 Dec;28(1):60.

32. Thiel H. Clinical governance, clinical risk management and the chiropractic profession. Clin Chiropr. 2003 Jun;6(2):45–8.

33. Thiel H. Incident reporting and learning systems for chiropractors - developments in Europe. J Can Chiropr Assoc. 2011 Sep;55(3):155–8.

34. Thiel H, Wangler M. 1.A.3 Reaching clinical quality management at a European level. Clin Chiropr. 2010 Mar;13(1):46–51.

35. Wangler M, Fujikawa R, Hestbæk L, Michielsen T, Raven TJ, Thiel HW, et al. Creating European guidelines for Chiropractic Incident Reporting and Learning Systems (CIRLS): relevance and structure. Chiropr Man Therap. 2011 Dec;19(1):9.

36. Chiropractic spinal manipulation of children under 12: Safer Care Victoria’s independent review. Victoria: Victorian Government; 2019 Oct.
