# Supplementary material for: Patient safety culture research within the chiropractic profession: a scoping review
Source: Chiropr Man Therap. 2025 Oct 21;33:46. doi: 10.1186/s12998-025-00605-z (PMC12538883; doi:10.1186/s12998-025-00605-z)
Supplement: Supplementary file 3 — Supplementary Material 3 [file 12998_2025_605_MOESM3_ESM.docx]

**Additional File 4 – Consultation Process**

**Executive Summary Sent to Stakeholders:**

**Patient Safety Culture Research within the Chiropractic Profession: A Scoping Review Executive Summary**

This scoping review synthesizes the extent, range, and nature of patient safety culture research activities in the chiropractic profession. Additionally, our findings are mapped against the Patient Safety Pyramid and WHO Global Patient Safety Action Plan (GPSAP) to identify areas of alignment and gaps in patient safety culture interventions or strategies. These insights aim to inform the development of a tailored guide for future chiropractic-specific patient safety research, emphasizing the evaluation of patient safety attitudes, beliefs, performance measurements, and strategic interventions to foster a robust patient safety culture within the profession. This scoping review follows the multi-stage process outlined in the Arksey and O’Malley framework, which includes the following stages: 1) research objective identification, 2) relevant study identification, 3) study selection, 4) data charting, 5) data synthesis and reporting, and 6) consultation (which is this process). This process has been reviewed by Parker University’s Institutional Review Board (IRB) and deemed exempt and approved (IRB #PUIRB-2025-2 ).

**Request for Feedback**

Scoping review methods encourage researchers to review their results with key stakeholder groups. Your expertise is instrumental in enhancing the quality and relevance of this work. We would greatly value your insights and suggestions on the following:

1. Whether we have comprehensively captured the relevant studies on patient safety culture in chiropractic and if there are any key articles or studies that we may have overlooked.
2. Feedback on the themes and frameworks used to interpret our findings, particularly in relation to the Patient Safety Pyramid and the WHO Global Patient Safety Action Plan.
3. Input on the clinical implications and future research recommendations outlined for the discussion of this review.

Below is an executive summary of the study objectives, methods, and specific items for your input. **Please return your feedback by February 14, 2025, to dwright02@parker.edu and/or kpohlman@parker.edu. If you'd prefer a discussion, we can arrange a video conference at your convenience.** Your time and input are invaluable in enhancing this study, and we sincerely appreciate your contributions.

A response to this email will imply consent to participate in the study.

**Also, please let us know if you give us permission to acknowledge your participation in this stage of our manuscript.**

**Sincerely,**

**Dr. Katherine A Pohlman and Dr. Debbie S Wright**

**Study Objective**

This scoping review explores and maps the extent, range, and nature of patient safety culture research carried out in the chiropractic profession.

**Methods**

The review included a systematic search of MEDLINE (via OVID), Index to Chiropractic Literature, AMED, CINAHL, and Google Scholar from database inception to December 13, 2024. The search strategy, developed in collaboration with a medical librarian, incorporated Medical Subject Heading (MeSH) terms and keywords for "patient safety," "adverse events," and "chiropractic." Peer reviewed, English-language studies focusing on patient safety culture in chiropractic care were included, while single case reports, opinion pieces, conference abstracts, and best practice documents were excluded. Screening was conducted in Covidence software, where 2 independent reviewers assessed titles, abstracts, and full-texts against eligibility criteria, resolving conflicts through consensus or a third-party referee. Data extraction, using a standardized form, captured study characteristics, methodology, and safety culture findings. Evidence tables summarized data, which were then analyzed quantitatively and qualitatively, identifying thematic clusters and mapping findings to the Patient Safety Pyramid (15) and WHO Global Patient Safety Action Plan (GPSAP) frameworks (1).

**Specific Items to review:**

1. ***Whether we have comprehensively captured the relevant studies and if there are any key articles, studies that we may have overlooked.***

Please reference:

**Table 1** (separate document) – Informational table of all included studies.

**Appendix A2** (separate document) – List of full-text excluded references and reasons for exclusions.

**Feedback requested:**

- 1. **Are there other studies of patient safety culture research in chiropractic that we should include in this scoping review?**
     1. **Please include reference and reason for each paper here.**
  2. **Are there any studies of patient safety culture research in chiropractic that we excluded that should be included in this scoping review?**
     1. **Please identify the paper and rationale for inclusion.**
  3. **Please include any additional feedback on included, excluded, or missed studies on patient safety culture research in chiropractic here.**

1. ***Feedback on the themes and frameworks used to interpret our findings, particularly in relation to the Patient Safety Pyramid and the WHO Global Patient Safety Action Plan.***

**Patient Safety Pyramid**

**Figure 1** – The Patient Safety Pyramid and Reported Percentages


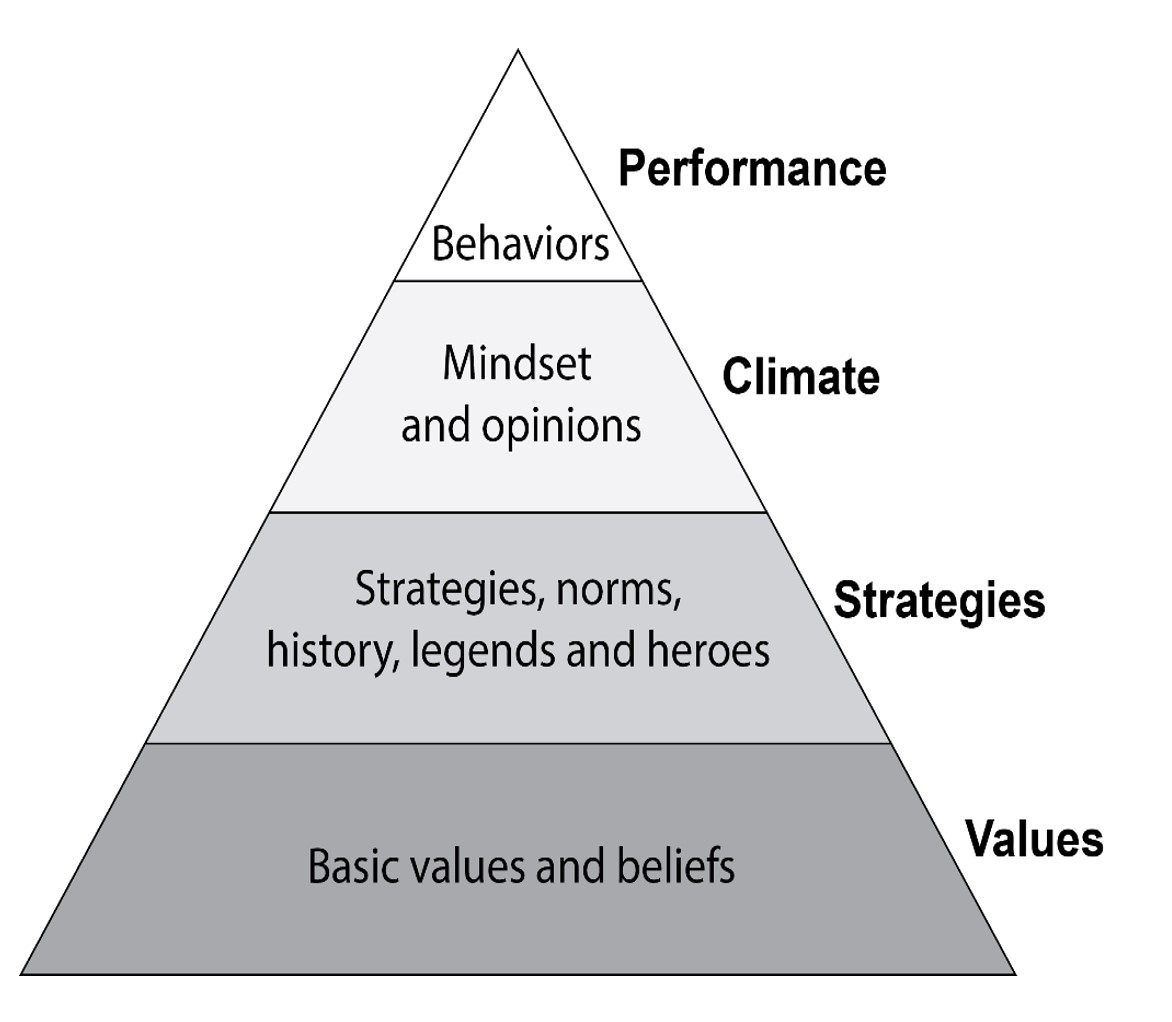


The Patient Safety Pyramid represents the dynamic nature of safety culture and incorporates core values and underlying assumptions as its base. Building on this foundation are organizational elements such as strategies, leadership, and policies. Higher up, the safety climate is shaped by the attitudes and opinions of the organization’s members, leading to safety performance at the peak, defined by behaviors and outcomes (15). Quantitative analysis of included studies identified the percentage of studies that addressed each level of the pyramid.

Figure 1 illustrates the distribution of study publications across the levels of the Patient Safety Pyramid (15). Most studies (83%) addressed multiple levels of the pyramid, highlighting a multifaceted approach to patient safety culture. Performance was discussed in 95% of the studies, while patient safety climate was covered in 48%, and strategies in 66%. However, only 23% of studies investigated core values or unquestioned assumptions associated with patient safety culture. Safety values are the foundation and critical for patient safety culture because they shape the attitudes, behaviors, and decisions of healthcare professionals, directly impacting the quality of care (15).

Half of all studies (48%) investigated safety climate, which includes attitudes and opinions of patients and providers towards patient safety. Surveys measuring safety climate were developed, validated, and evaluated primarily by the SafetyNet team (6,66,70). Their recommendations to address this existing gap include more regular inclusion of safety climate surveys in research initiatives and the subsequent development of a patient safety culture database for SMT providers to help with more advanced quality improvement initiatives and to measure their impact. Safety values and safety climate are the underpinnings of a robust chiropractic patient safety culture, and addressing these levels of the pyramid will directly influence the behaviors of chiropractors and their outcomes (15).

| **Feedback requested** | **:** |
| --- | --- |
| **Please offer any feedback about our findings as they related to the Patient Safety Pyramid here.** | |

# The [WHO Global Patient Safety Action Plan](https://www.who.int/teams/integrated-health-services/patient-safety/policy/global-patient-safety-action-plan) (GPSAP)

The GPSAP is a strategic framework developed by the WHO and consists of 7 strategic objectives to guide stakeholders in improving patient safety and reducing preventable harm in healthcare settings worldwide. We mapped patient safety culture studies to the 7 strategic objectives to determine the number studies that addressed each objective either directly or indirectly. A study was classified as direct if the reviewing committee determined that it explicitly addressed 1 of the WHO GPSAP objectives and investigated its principles as a primary focus. Alternatively, a study was considered indirect if it provided information relevant to a WHO GPSAP objective but did so incidentally or as part of a broader discussion. While not the central focus, indirect studies still offered valuable insights that contributed to understanding or addressing the objective.

Figure 2 (next page) illustrates a mapping of included studies to 7 strategic objectives of the WHO GPSAP (1). The WHO GPSAP objectives are supported by a nearly equal number of studies that directly (n=69) and indirectly (n=66) address them along with identified action items and recommendations for future research. While many studies had direct implications for enhancing the safety of clinical processes, notable gaps were observed in areas such as policy, leadership, administrative support, and the synergy, partnerships, and solidarity objective. Although some studies directly addressed patient engagement, the majority explored this objective through an indirect lens.

As shown in Figure 2, significant gaps related to the chiropractic profession were identified in alignment with the WHO GPSAP. Few studies addressed patient safety policies, and none did so directly. Indirectly, some studies provided recommendations for regulatory or accreditation policies that were found to be related to sanitization, informed consent, and international competency standards for patient safety and practice (19,33,57). To date, minimal progress has been made in advancing patient safety culture through foundational policy reforms governing chiropractic practice. Targeted efforts are essential to achieve the WHO GPSAP’s goal of zero avoidable harm across all aspects of healthcare planning and delivery.

Gaps were also identified in objectives related to building high-reliability systems and fostering synergy and partnerships for patient safety. Only 2 studies directly addressed each objective, with some overlap in action items. For high-reliability systems, action items highlighted the need for leadership to strengthen patient safety culture (7,22) and promote intra-organizational collaboration for reporting and learning from safety data (71). However, critical aspects such as human factors, ergonomics, and good governance principles remained unaddressed. Similarly, action items for synergy and partnership emphasized the importance of interprofessional collaboration (21,39,60,71,73) and education initiatives (28,61,65). However, they lacked attention to developing patient safety networks that span regions and professions.

Notably, only about 1/3 of studies addressing patient and family engagement involved direct contact with patients. The remainder discussed patient engagement conceptually without gathering input directly from patients. Patient perspectives were evaluated concerning adverse event (AE) definitions (40), AE mitigation (66), and informed consent (55). Future research recommendations consistently emphasized the need for greater exploration of the patient perspective to enhance all facets of patient safety culture (58,69)

**Feedback requested:**

**Please offer any feedback about our findings as they related to the WHO Global Patient Safety Action Plan here.**

**Figure 2** – Mapping of Studies to WHO GPSAP Strategic Objectives

| **Strategic Objectives** | **Studies Addressing the Objective (Directly)** | **Action Items** | **Future Research Recommendations** |
| --- | --- | --- | --- |
| 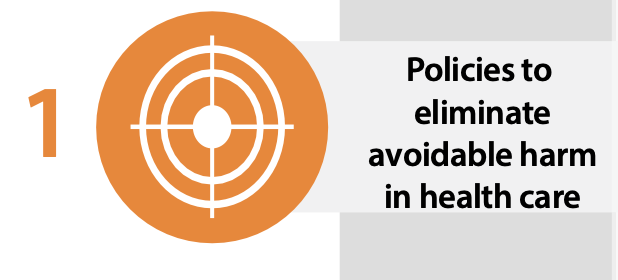 | 3 (0) | 1. Regulatory policies regarding informed consent | None provided |
| 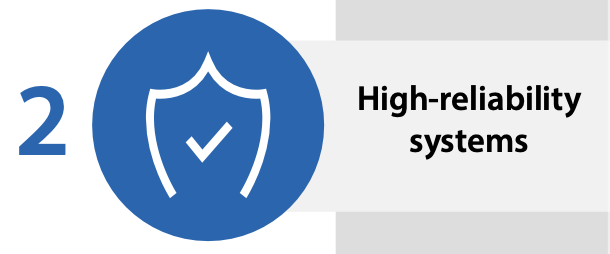 | 9 (2) | 1. Need for leadership to support patient safety culture 2. Collaboration around, reporting of, and learning from safety information | None provided |
| 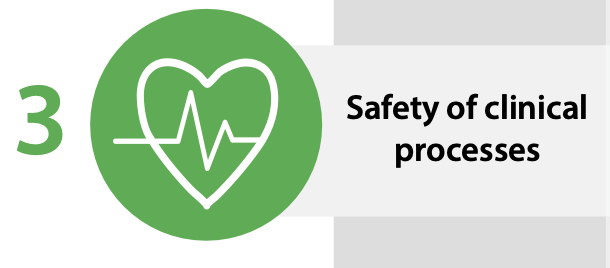 | 45 (30) | 1. Protocols for sanitization 2. Education, office practices, and clinical decision making to enhance safety 3. Appropriate informed consent procedures and patient communication about expectations | 1. Sanitization and associated risks 2. Safety of special populations (older, pediatric) 3. Various adverse event related factors including incidence, predictors, causation, and mitigation |
| 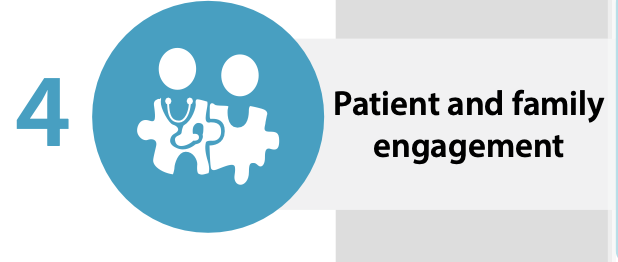 | 18 (6) | 1. Patient input into adverse event classification systems is needed 2. Excellence in patient communication and engagement to enhance patient safety culture | 1. Perspectives of patients who have experienced adverse events and strategies for mitigation 2. Patient perspective needs to be included in future reporting system research 3. Patient perspectives on informed consent |
| 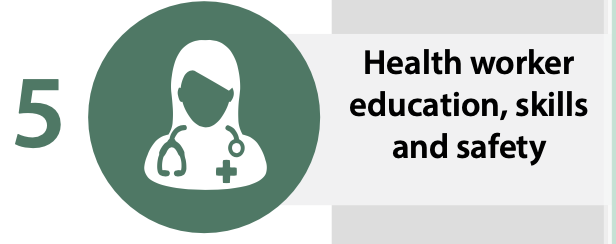 | 19 (12) | 1. Enhance graduate training to improve safety 2. Enhance collaboration, knowledge exchange and learning opportunities for clinicians 3. Incorporate safety into accreditation/regulation | 1. Patient safety culture surveys in future trials 2. Ideal communication strategies for practitioners 3. Evaluate creation and enforcement of Council on Chiropractic Education competency standards |
| 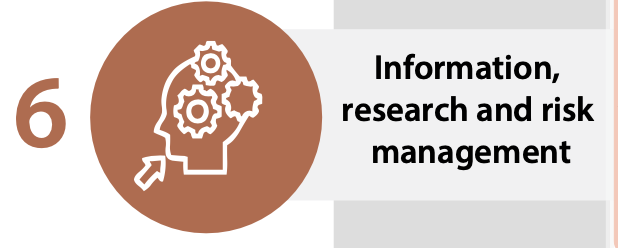 | 32 (21) | 1. Adopt a standardized approach to classifying/reporting adverse events in clinical and research settings 2. Adopt incidence reporting and learning systems for the chiropractic profession | 1. Standardize reporting of adverse events in trials 2. Collect better risk data to inform clinicians with the aim of improving consent processes 3. Explore reporting and learning systems to make them more effective |
| 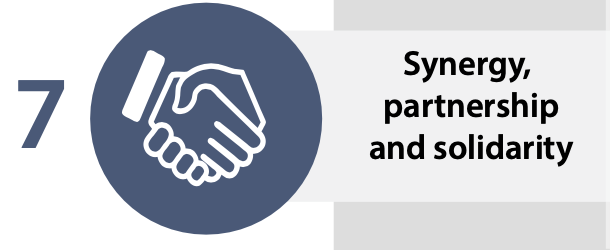 | 13 (2) | 1. Interprofessional collaboration to improve patient safety culture and practice 2. Interprofessional education regarding patient safety culture | 1. Evaluate attitudes towards interprofessional co-management of patients, and ways to enhance coordination of care |

***3. Input on the clinical implications and additional research recommendations outlined for the discussion of this review.***

Advancing patient safety culture in chiropractic care requires coordinated efforts across policy development, research priorities, and actionable clinical strategies. At the policy level, there is an urgent need to establish regulatory frameworks and international competency standards addressing key gaps, such as standardized informed consent processes, active surveillance systems for adverse events (AEs), and patient safety training in both educational and professional settings. Policymakers should also prioritize implementing reporting and learning systems that align with the WHO GPSAP objectives, such as fostering high-reliability systems and promoting partnerships that transcend regional and professional boundaries.

Research efforts must focus on addressing the lack of standardized terms, definitions, and classification systems for AEs, as inconsistencies in these areas hinder effective AE reporting and data pooling. Future research should emphasize evaluating adherence to frameworks like the CONSORT guidelines for trial reporting and developing validated tools for AE data collection in clinical settings. Moreover, incorporating patient perspectives into safety research—such as gathering input on AE definitions and mitigation strategies—can ensure that findings are both patient-centered and practical.

Clinically, chiropractors should enhance their informed consent practices to include comprehensive discussions of risks, benefits, and alternatives, moving beyond formal documentation to foster shared decision-making. History-taking and clinical decision-making skills must be improved to identify risk factors for AEs and adjust treatments accordingly, particularly for high-risk groups such as pediatric and older patients. For example, studies suggest that most AEs occur early in treatment, highlighting the importance of carefully monitoring and adapting care during initial sessions.

Implementing active surveillance systems that build upon the existing Chiropractic Patient Incident Reporting and Learning System (CPiRLS), is critical. These systems provide high-quality data on AEs, allowing for actionable insights into risk factors and the development of targeted mitigation strategies. Workplace measures, such as reducing provider burnout and fostering interprofessional collaboration, are equally vital for creating a safer environment for both providers and patients.

By addressing these interconnected gaps through targeted policies, rigorous research, and clinical interventions, the chiropractic profession can strengthen its patient safety culture, align with global patient safety goals, and reduce avoidable harm in practice.

**Feedback requested:**

**Please offer any feedback about our findings as they related to the clinical implications and future research on patient safety culture in chiropractic here.**

# Study and Team Information

**OSF Study Protocol link:** [**https://osf.io/5drsv**](https://osf.io/5drsv)

**Review Authorship Team Members**

Debbie S. Wright Parker University, Private practice

Maranda Kleppe Palmer College of Chiropractic

Brian C. Coleman Yale University

Martha Funabashi Canadian Memorial Chiropractic College

Amy G. Ferguson Parker University

Richard Brown World Federation of Chiropractic

Sidney M. Rubinstein Vrije Universiteit Amsterdam

Katherine A. Pohlman (Corresponding Author) Parker University

Stacie A. Salsbury Palmer College of Chiropractic

**References**

1. Global Patient Safety Action Plan 2021-2030 [Internet]. Geneva: World Health Organization; 2021 [cited 2024 Oct 10]. Available from: https://www.who.int/teams/integrated-health-services/patient-safety/policy/globalpatient-safety-action-plan

1. Kohn LT, Corrigan J, Donaldson MS, editors. To err is human: building a safer health system. Washington, D.C: National Academy Press; 2000. 287 p.

1. The conceptual framework for the international classification for patient safety

[Internet]. Geneva: World Health Organization; 2009 [cited 2024 Oct 10]. Available from:

https://www.who.int/publications/i/item/WHO-IER-PSP-2010.2

1. Singer SJ, Gaba DM, Falwell A, Lin S, Hayes J, Baker L. Patient safety climate in 92 US hospitals: differences by work area and discipline. Med Care. 2009 Jan;47(1):23–31.

1. Nahin RL, Rhee A, Stussman B. Use of complementary health approaches overall and for pain management by US adults. JAMA. 2024 Feb 20;331(7):613.

1. Funabashi M, Pohlman KA, Mior S, O’Beirne M, Westaway M, De Carvalho D, et al. SafetyNET Community-based patient safety initiatives: development and application of a Patient Safety and Quality Improvement Survey. J Can Chiropr Assoc. 2018 Dec;62(3):130–42.

1. Pohlman KA, Funabashi M, Ndetan H, Hogg-Johnson S, Bodnar P, Kawchuk G. Assessing adverse events after chiropractic care at a chiropractic teaching clinic: an active- surveillance pilot study. J Manipulative Physiol Ther. 2020 Nov;43(9):845–54.

1. Thomas M, Swait G, Finch R. Ten years of online incident reporting and learning using CPiRLS: implications for improved patient safety. Chiropr Man Ther. 2023 Feb 15;31(1):9.

1. Arksey H, O’Malley L. Scoping studies: towards a methodological framework. Int J Soc Res Methodol. 2005 Feb;8(1):19–32.

1. Levac D, Colquhoun H, O’Brien KK. Scoping studies: advancing the methodology. Implement Sci. 2010 Dec;5(1):69.

1. Tricco AC, Lillie E, Zarin W, O’Brien KK, Colquhoun H, Levac D, et al. PRISMA extension for scoping reviews (PRISMA-ScR): checklist and explanation. Ann Intern Med. 2018 Oct 2;169(7):467–73.

1. Open Science Framework Registry [Internet]. [cited 2024 Oct 2]. Patient safety culture research within the chiropractic profession: a scoping review protocol. Available from: <https://osf.io/bcrkm/>
2. McGowan J, Sampson M, Salzwedel DM, Cogo E, Foerster V, Lefebvre C. PRESS Peer Review of Electronic Search Strategies: 2015 guideline statement. J Clin Epidemiol. 2016 Jul;75:40–6.

1. Covidence systematic review software [Internet]. Melbourne, Australia: Veritas Health Innovation; Available from: www.covidence.org

1. Patankar MS, editor. Safety culture: building and sustaining a cultural change in aviation and healthcare. Aldershot: Ashgate; 2012. 243 p.

1. Pokras R, Iler L. Bacterial load on the chiropractic adjusting table. J Aust Chiropr Assoc. 1990;20:85–90.

1. Terret A, Kleynhans A. Complications from manipulation of the low back. Chiropr J Aust. 1992;22:129–40.

1. Jamison JR. Informed consent: an Australian case study. J Manipulative Physiol Ther. 1998 Jun;21(5):348–55.

1. Langworthy JM, le Fleming C. Consent or submission? The practice of consent within UK chiropractic. J Manipulative Physiol Ther. 2005 Jan;28(1):15–24.

1. Bifero A, Prakash J, Bergin J. The role of chiropractic adjusting tables as reservoirs for microbial diseases. Am J Infect Control. 2006 Apr;34(3):155–7.

1. Smith M, Greene BR, Haas M, Allareddy V. Intra-professional and inter-professional referral patterns of chiropractors. Chiropr Osteopat. 2006 Dec;14(1):12.

1. Thiel H, Bolton J. The reporting of patient safety incidents—first experiences with the chiropractic reporting and learning system (CRLS): A pilot study. Clin Chiropr. 2006 Sep;9(3):139–49.

1. Evans M, Breshears J. Attitudes and behaviors of chiropractic college students on hand sanitizing and treatment table disinfection: results of initial survey and focus group. J Am Chiropr Assoc. 2007;44(4):13–23.

1. Evans MW, Breshears J, Campbell A, Husbands C, Rupert R. Assessment and risk reduction of infectious pathogens on chiropractic treatment tables. Chiropr Osteopat. 2007 Dec;15(1):8.

1. Langworthy JM, Cambron J. Consent: its practices and implications in United Kingdom and United States chiropractic practice. J Manipulative Physiol Ther. 2007 Jul;30(6):419– 31.
2. Rubin D. Triage and case presentations in a chiropractic pediatric clinic. J Chiropr Med. 2007 Sep;6(3):94–8.

1. Rubinstein SM, Leboeuf-Yde C, Knol DL, De Koekkoek TE, Pfeifle CE, Van Tulder MW. The benefits outweigh the risks for patients undergoing chiropractic care for neck pain: a prospective, multicenter, cohort study. J Manipulative Physiol Ther. 2007 Jul;30(6):408– 18.

1. Vohra S, Johnston BC, Cramer K, Humphreys K. Adverse events associated with pediatric spinal manipulation: a systematic review. Pediatrics. 2007 Jan 1;119(1):e275–83.

1. Evans MW, Campbell A, Husbands C, Breshears J, Ndetan H, Rupert R. Cloth-covered chiropractic treatment tables as a source of allergens and pathogenic microbes. J Chiropr Med. 2008 Mar;7(1):34–8.

1. Gunn SJ, Thiel HW, Bolton JE. British Chiropractic Association members’ attitudes towards the Chiropractic Reporting and Learning System: a qualitative study. Clin Chiropr. 2008 Jun;11(2):63–9.

1. Miller JE, Benfield K. Adverse effects of spinal manipulative therapy in children younger than 3 years: a retrospective study in a chiropractic teaching clinic. J Manipulative Physiol Ther. 2008 Jul;31(6):419–23.

1. Rubinstein SM, Leboeuf-Yde C, Knol DL, De Koekkoek TE, Pfeifle CE, Van Tulder MW. Predictors of adverse events following chiropractic care for patients with neck pain. J Manipulative Physiol Ther. 2008 Feb;31(2):94–103.

1. Evans MW, Ramcharan M, Floyd R, Globe G, Ndetan H, Williams R, et al. A proposed protocol for hand and table sanitizing in chiropractic clinics and education institutions. J

Chiropr Med. 2009 Mar;8(1):38–47.

1. Miller JE. Safety of chiropractic manual therapy for children: How are we doing? J Clin Chiropr Pediatr. 2009;10(2):655–60.

1. Carlesso LC, Gross AR, Santaguida PL, Burnie S, Voth S, Sadi J. Adverse events associated with the use of cervical manipulation and mobilization for the treatment of neck pain in adults: A systematic review. Man Ther. 2010 Oct;15(5):434–44.

1. Carnes D, Mullinger B, Underwood M. Defining adverse events in manual therapies: a modified Delphi consensus study. Man Ther. 2010 Feb;15(1):2–6.

1. Langworthy JM, Forrest L. Withdrawal rates as a consequence of disclosure of risk associated with manipulation of the cervical spine. Chiropr Osteopat. 2010 Dec;18(1):27.
2. Leach RA. Patients with symptoms and signs of stroke presenting to a rural chiropractic practice. J Manipulative Physiol Ther. 2010 Jan;33(1):62–9.

1. Smith M, Bero L, Carber L. Could chiropractors screen for adverse drug events in the community? Survey of US chiropractors. Chiropr Osteopat. 2010 Dec;18(1):30.

1. Carlesso LC, Cairney J, Dolovich L, Hoogenes J. Defining adverse events in manual therapy: An exploratory qualitative analysis of the patient perspective. Man Ther. 2011 Oct;16(5):440–6.

1. Gleberzon B. A narrative review of the published chiropractic literature regarding older patients from 2001–2010. J Can Chiropr Assoc. 2011;55(2):76–95.

1. Turner LA, Singh K, Garritty C, Tsertsvadze A, Manheimer E, Wieland LS, et al. An evaluation of the completeness of safety reporting in reports of complementary and alternative medicine trials. BMC Complement Altern Med. 2011 Dec;11(1):67.

1. Dagenais S, Brady O, Haldeman S. Shared decision making through informed consent in chiropractic management of low back pain. J Manipulative Physiol Ther. 2012 Mar;35(3):216–26.

1. Sadr S, Pourkiani-Allah-Abad N, Stuber KJ. The treatment experience of patients with low back pain during pregnancy and their chiropractors: a qualitative study. Chiropr Man Ther. 2012 Dec;20(1):32.

1. Walker BF, Hebert JJ, Stomski NJ, Clarke BR, Bowden RS, Losco B, et al. Outcomes of usual chiropractic. The OUCH randomized controlled trial of adverse events. Spine. 2013 Sep;38(20):1723–9.

1. Wangler M, Peterson C, Zaugg B, Thiel H, Finch R. How do chiropractors manage clinical risk? A questionnaire study. Chiropr Man Ther. 2013 Dec;21(1):18.

1. Boucher P, Robidoux S. Lumbar disc herniation and cauda equina syndrome following spinal manipulative therapy: a review of six court decisions in Canada. J Forensic Leg Med. 2014 Feb;22:159–69.

1. Jevne J, Hartvigsen J, Christensen HW. Compensation claims for chiropractic in Denmark and Norway 2004–2012. Chiropr Man Ther. 2014 Dec;22(1):37.

1. Pohlman KA, O’Beirne M, Thiel H, Cassidy JDavid, Mior S, Hurwitz EL, et al. Development and validation of providers’ and patients’ measurement instruments to evaluate adverse events after spinal manipulation therapy. Eur J Integr Med. 2014 Aug;6(4):451–66.
2. Hebert JJ, Stomski NJ, French SD, Rubinstein SM. Serious adverse events and spinal manipulative therapy of the low back region: a systematic review of cases. J Manipulative Physiol Ther. 2015 Nov;38(9):677–91.

1. Marchand AM. A literature review of pediatric spinal manipulation and chiropractic manipulative therapy: evaluation of consistent use of safety terminology. J Manipulative Physiol Ther. 2015 Nov;38(9):692–8.

1. Marchand AM. A proposed model with possible implications for safety and technique adaptations for chiropractic spinal manipulative therapy for infants and children. J Manipulative Physiol Ther. 2015 Nov;38(9):713–26.

1. Puentedura EJ, O’Grady WH. Safety of thrust joint manipulation in the thoracic spine: a systematic review. J Man Manip Ther. 2015 Jul;23(3):154–61.

1. Todd AJ, Carroll MT, Robinson A, Mitchell EKL. Adverse events due to chiropractic and other manual therapies for infants and children: a review of the literature. J

Manipulative Physiol Ther. 2015 Nov;38(9):699–712.

1. Winterbottom M, Boon H, Mior S, Facey M. Informed consent for chiropractic care:

comparing patients’ perceptions to the legal perspective. Man Ther. 2015 Jun;20(3):463–

8.

1. Gorrell LM, Engel RM, Brown B, Lystad RP. The reporting of adverse events following spinal manipulation in randomized clinical trials—a systematic review. Spine J. 2016 Sep;16(9):1143–51.

1. Innes SI, Leboeuf-Yde C, Walker BF. Similarities and differences of graduate entry-level competencies of chiropractic councils on education: a systematic review. Chiropr Man Ther. 2016 Dec;24(1):1.

1. Pohlman KA, Carroll L, Hartling L, Tsuyuki R, Vohra S. Attitudes and opinions of Doctors of Chiropractic specializing in pediatric care toward patient safety: a cross-sectional survey. J Manipulative Physiol Ther. 2016 Sep;39(7):487–93.

1. Pohlman KA, Carroll L, Hartling L, Tsuyuki RT, Vohra S. Barriers to implementing a reporting and learning patient safety system: pediatric chiropractic perspective. J Evid- Based Complement Altern Med. 2016 Apr;21(2):105–9.

1. Rozmovits L, Mior S, Boon H. Exploring approaches to patient safety: the case of spinal manipulation therapy. BMC Complement Altern Med. 2016 Dec;16(1):164.

1. Porcino A, Solomonian L, Zylich S, Gluvic B, Doucet C, Vohra S. Pediatric training and practice of Canadian chiropractic and naturopathic doctors: a 2004–2014 comparative study. BMC Complement Altern Med. 2017 Dec;17(1):512.

1. Swait G, Finch R. What are the risks of manual treatment of the spine? A scoping review for clinicians. Chiropr Man Ther. 2017 Dec;25(1):37.

1. Innes SI, Leboeuf-Yde C, Walker BF. Chiropractic student choices in relation to indications, non-indications and contra-indications of continued care. Chiropr Man Ther. 2018 Dec;26(1):3.

1. Zorzela L, Mior S, Boon H, Gross A, Yager J, Carter R, et al. Tool to assess causality of direct and indirect adverse events associated with therapeutic interventions. Curr Med Res Opin. 2018 Mar 4;34(3):407–14.

1. Salsbury SA, Vining RD, Hondras MA, Wallace RB, Lyons KJ, Killinger LZ, et al. Interprofessional attitudes and interdisciplinary practices for older adults with back pain among Doctors of Chiropractic: a descriptive survey. J Manipulative Physiol Ther. 2019 May;42(4):295–305.

1. Funabashi M, Pohlman KA, Goldsworthy R, Lee A, Tibbles A, Mior S, et al. Beliefs, perceptions and practices of chiropractors and patients about mitigation strategies for benign adverse events after spinal manipulation therapy. Chiropr Man Ther. 2020 Dec;28(1):46.

1. Pohlman KA, Carroll L, Tsuyuki RT, Hartling L, Vohra S. Comparison of active versus passive surveillance adverse event reporting in a paediatric ambulatory chiropractic care setting: a cluster randomised controlled trial. BMJ Open Qual. 2020 Nov;9(4):e000972.

1. Pohlman KA, Salsbury SA, Funabashi M, Holmes MM, Mior S. Patient safety in chiropractic teaching programs: a mixed methods study. Chiropr Man Ther. 2020 Dec;28(1):50.

1. To D, Tibbles A, Funabashi M. Lessons learned from cases of rib fractures after manual therapy: a case series to increase patient safety. J Can Chiropr Assoc. 2020 Apr;64(1):7– 15.
2. Alcantara J, Whetten A, Alcantara J. Towards a safety culture in chiropractic: the use of the safety, communication, operational reliability, and engagement (SCORE) questionnaire. Complement Ther Clin Pract. 2021 Feb;42:101266.

1. Funabashi M, Holmes MM, Pohlman KA, Salsbury S, O’Beirne M, Vohra S, et al. “Doing our best for patient safety”: an international and interprofessional qualitative study with spinal manipulative therapy providers in community-based settings. Musculoskelet Sci Pract. 2021 Dec;56:102470.

1. Weis C, Stuber K, Murnaghan K. Adverse events from spinal manipulations in the pregnant and postpartum periods: a systematic review and update. J Can Chiropr Assoc. 2021;65(1):32–49.

1. Funabashi M, Gorrell LM, Pohlman KA, Bergna A, Heneghan NR. Definition and classification for adverse events following spinal and peripheral joint manipulation and mobilization: a scoping review. Chen TH, editor. PLOS ONE. 2022 Jul 15;17(7):e0270671.

1. Stickler K, Kearns G. Spinal manipulation and adverse event reporting in the pregnant patient limits estimation of relative risk: a narrative review. J Man Manip Ther. 2023 May 4;31(3):162–73.

1. Dolbec A, Doucet C, Pohlman KA, Sobczak S, Pagé I. Assessing adverse events associated with chiropractic care in preschool pediatric population: a feasibility study. Chiropr Man Ther. 2024 Mar 13;32(1):9.

1. Pohlman KA, Funabashi M, O’Beirne M, Cassidy JD, Hill MD, Hurwitz EL, et al. What’s the harm? Results of an active surveillance adverse event reporting system for chiropractors and physiotherapists. Mohsenifar H, editor. PLOS ONE. 2024 Aug 19;19(8):e0309069.

1. Schulz KF, Altman DG, Moher D, for the CONSORT Group. CONSORT 2010 Statement:

updated guidelines for reporting parallel group randomised trials. BMJ. 2010 Mar 23;340(mar23 1):c332–c332.

1. Murray J, Sorra J, Gale B, Mossburg S. Ensuring patient and workforce safety culture in healthcare [Internet]. Rockville, MD: Agency for Healthcare Research and Quality (US); 2024. Available from: https://psnet.ahrq.gov/perspective/ensuring-patient-and- workforce-safety-culture-healthcare

1. Vohra S, Kawchuk GN, Boon H, Caulfield T, Pohlman KA, O’Beirne M. SafetyNET: An interdisciplinary research program to support a safety culture for spinal manipulation therapy. Eur J Integr Med. 2014 Aug;6(4):473–7.

1. Leboeuf-Yde C, Hennius B, Rudberg E, Leufvenmark P, Thunman M. Side effects of chiropractic treatment: a prospective study. J Manipulative Physiol Ther. 1997 Oct;20(8):511–5.

1. Cagnie B, Vinck E, Beernaert A, Cambier D. How common are side effects of spinal manipulation and can these side effects be predicted? Man Ther. 2004 Aug;9(3):151–6.
2. Ryan AT, Too LS, Bismark MM. Complaints about chiropractors, osteopaths, and physiotherapists: a retrospective cohort study of health, performance, and conduct concerns. Chiropr Man Ther. 2018 Dec;26(1):12.

1. Toth E, Lawson D, Nykoliation J. Chiropractic complaints and disciplinary cases in Canada. J Can Chiropr Assoc. 1998;42(4):229–42.
2. Maiers MJ, Foshee WK, Henson Dunlap H. Culturally sensitive chiropractic care of the transgender community: a narrative review of the literature. J Chiropr Humanit. 2017 Dec;24(1):24–30.

1. Coulter ID, Singh BB, Riley D, Der-Martirosian C. Interprofessional referral patterns in an integrated medical system. J Manipulative Physiol Ther. 2005 Mar;28(3):170–4.

**Summary of Feedback Received:**

**Scoping Review Consultation**

**Invited:**

WFC Research Committee (n=15)

CAA Patient Safety Roundtable (n=26)

Royal College of Chiropractors (n=4)

**Declined Due to Authorship:**

WFC Research Committee (n=4)

CAA Patient Safety Roundtable (n=1)

Royal College of Chiropractors (n=0)

**Feedback Received:**

WFC Research Committee (n=0)

CAA Patient Safety Roundtable (n=3)

Royal College of Chiropractors (n=1)

**Feedback Comments with proposed remarks in blue**

*From CAA Roundtable Participant 1:*

1. Whether we have comprehensively captured the relevant studies on patient safety culture in chiropractic and if there are any key articles or studies that we may have overlooked.
    -I reviewed your sample against what I could find and there was no omissions. Excellent and thorough review of the area.
   No changes needed.
2. Feedback on the themes and frameworks used to interpret our findings, particularly in relation to the Patient Safety Pyramid and the WHO Global Patient Safety Action Plan.
    - I thought the approach, themes and frameworks were sound and appropriate given the Pyramid and the action plan. It was well written and thorough.
   No changes needed.
3. Would we like to provide an example of how inconsistency in AE definitions has led to difficulties in research or policy implementation? We had discussed how previous attempts at consensus on AE definitions have close but "no cigar" and this has lead to our current situation. Katie, you discussed this with the CAA during one of our meetings and found it very helpful. Might it be helpful here?
   This has been elaborated on in the manuscript.
4. I liked the final sentence but thought this might have more impact with the following change: From: “By addressing these interconnected gaps through targeted policies, rigorous research, and clinical interventions, the chiropractic profession can strengthen its patient safety culture, align with global patient safety goals, and reduce avoidable harm in practice.” To: "By addressing these interconnected gaps through targeted policies, rigorous research, and clinical interventions, the chiropractic profession can foster a robust patient safety culture, align with global standards, and reduce preventable harm in daily practice."
   This text is from the executive summary only, not in the manuscript. No change to make

*From CAA Roundtable Participant 2:*

1. After a quick review of the articles, I have no suggestions about other articles to add. My clinical comment on the research paper and the scoping study is that I do not see anything that directly covers the diversity and complexity of patients that chiropractors may encounter in a daily practice. While we know that pediatrics and elderly are higher risk initially, there is also other patient characteristics that also increase risk. For example, patients from a rural location often present with very complex and high-risk factors because there are very little alternatives available to them. There is a shortage of primary health care practitioners and a very weak referral system. Frequently, patients come to a chiropractor first because they have an existing relationship with them that they trust more than with a locum doctor or a family physician that they cannot get to see for months.
   No changes made, but perspective noted.
2. Reporting adverse effects may not happen as frequently if doctors feel that there will be negative or punitive consequences from their professional colleges.
   No changes made, but perspective noted.
3. I think it will be difficult to establish standardized criteria because of the complexity of patients seen and the diversity of techniques among chiropractors.
   No changes needed.
4. Interprofessional collaboration will be challenging because of existing stereotypes and perceptions about the chiropractic profession from medical doctors, pharmacists, and other health providers. We need to educate as well as to collaborate.
   No changes made, but perspective noted.
5. How and who sets the criteria to measure safety in a chiropractic setting? This will be hard even among chiropractors, let alone other health care professionals and patients. For example, there is not a standardized, universally accepted definition for something as common as a concussion. If we can’t agree on a definition for a problem we commonly treat, how can we measure and define risk factors or measure AEs that may be associated with treating that problem.
   No changes made, but perspective noted.
6. The CCPA has been a doing a consistently good job on informed consent, and how we should explain it to our patients. They have regularly provided updates over time to help keep informed consent current for patients and practitioners.
   No changes needed.
7. The hidden friction point is that a discussion of patient safety means that some control over the scope of practice for chiropractors will have to be shared with patients and other stakeholders.

No changes needed.

*From CAA Roundtable Participant 3:*

1. Methodology discussed in detail, resulting in no suggested changes to manuscript.

No changes needed.

*From RCC Member 1:*

1. This might be better titled and explained as the Patient Safety CULTURE pyramid. i.e. adapted from the Safety Culture Pyramid that originated in the aviation industry.

Wording changed in the manuscript to add “Culture”.

1. I'd quite like to know whether studies referenced these levels? i.e. is the safety culture pyramid explicitly used to guide the research identified. As probably not part of the prior aims though, this might be a point for 'discussion' Similarly (perhaps for discussion) it might also be pertinent to track publications and their focus over time - is there evidence of development of focus on safety culture? A quick scroll down of the included studies, as they are organised by date in the Excel file, suggests earlier studies were focussed on 'behaviours' and also on research or reporting of AEs. SI reporting first occurs in 2008, and then increases following your 2014 work. Later studies have much more focus on attitudes and opinions to patient safety and on SI reporting.
   No changes made, but perspective noted.
2. The point about lack of patient safety culture research evaluating undergraduate training (curricula, accreditation, knowledge and skills acquisition by learners) stood out to me, so I agree this point. however, it doesn’t really come across in the results presented earlier. Perhaps because there are 19 studies mapping to the GPSAP item 'health worker education...' but most of these are around knowledge and skills among practitioners, or in some cases students, but not evaluating teaching and learning (i.e. the education to develop safety culture).

Reviewed manuscript to ensure patient safety education appropriately discussed.
