# Supplementary material for: Patient safety culture research within the chiropractic profession: a scoping review
Source: Chiropr Man Therap. 2025 Oct 21;33:46. doi: 10.1186/s12998-025-00605-z (PMC12538883; doi:10.1186/s12998-025-00605-z)

**Additional File 3- Preferred Reporting Items for Systematic Reviews and Meta-Analyses Extension for Scoping Reviews (PRISMA-ScR) Checklist**

**
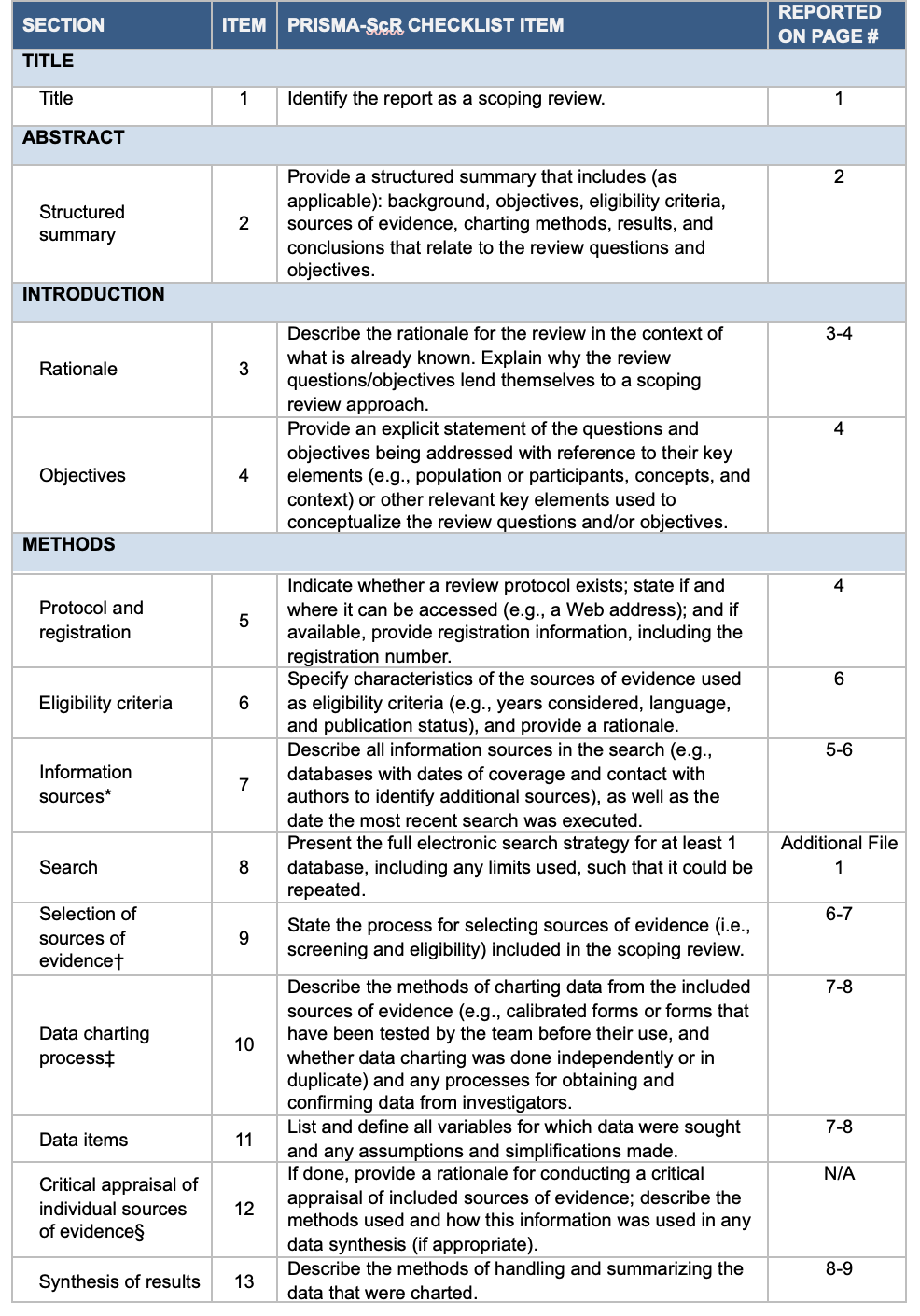
**


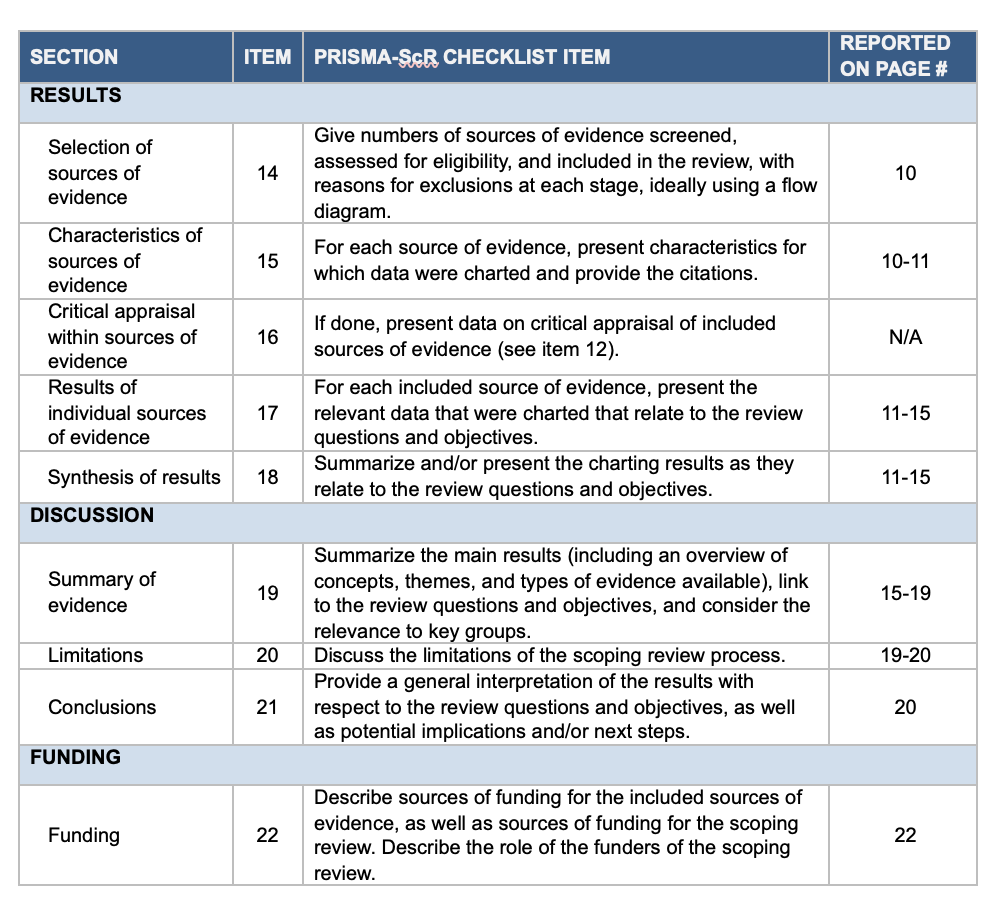

Supplement: Supplementary file 4 — Supplementary Material 4 [file 12998_2025_605_MOESM4_ESM.docx]
